# Cell penetration of oxadiazole-containing macrocycles†

**DOI:** 10.1039/d3cb00201b

**Published:** 2024-01-15

**Authors:** Sungjoon Huh, Nefeli Batistatou, Jing Wang, George J. Saunders, Joshua A. Kritzer, Andrei K. Yudin

**Affiliations:** a Davenport Research Laboratories, University of Toronto 80 St. George St Toronto Ontario M5S 3H6 Canada andrei.yudin@utoronto.ca; b Department of Chemistry, Tufts University 62 Talbot Ave Medford MA 02155 USA johua.kritzer@tufts.edu

## Abstract

Passive membrane permeability is an important property in drug discovery and biological probe design. To elucidate the cell-penetrating ability of oxadiazole-containing (Odz) peptides, we employed the Chloroalkane Penetration Assay. The present study demonstrates that Odz cyclic peptides can be highly cell-penetrant depending on the position of specific side chains and the chloroalkane tag. Solution NMR shows that Odz cyclic peptides adopt a β-turn conformation. However, despite observing high cell penetration, we observed low passive permeability in experiments with artificial membranes. These findings highlight the complexity of controlling cell penetration for conformationally sensitive macrocycles and suggest that Odz cyclic peptides may provide a framework for designing cell-penetrant cyclic peptides.

## Abstract 2

Cellular permeability is an important property in drug discovery and biological probe design. Here we investigate the effect of oxadiazole grafts on cellular permeability using Chloroalkane Penetration Assay.

## Graphical abstract

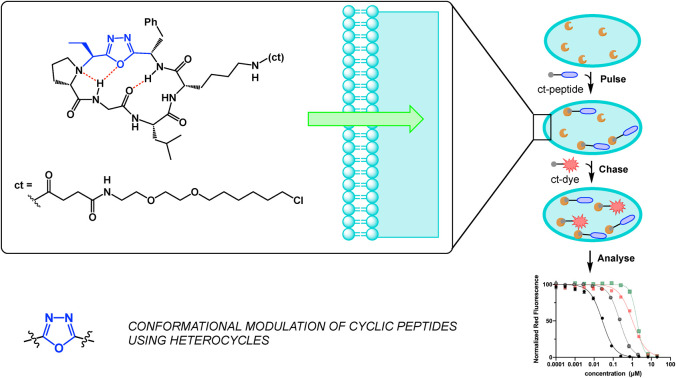

## Introduction

Cyclic peptides are a valuable class of molecules in drug discovery because of their ability to resist proteolytic degradation, pre-organize binding epitopes, and block protein–protein interactions.^1–3^ However, despite these advantages, one of the major hurdles that cyclic peptides face is their poor bioavailability, which limits their translation into therapeutics.^4^ Most cyclic peptides are traditionally viewed as unlikely to be bioavailable^5,6^ owing to their high molecular weight and high number of hydrogen bond donors and acceptors, which increase their overall 3-dimensional polar surface area.^7,8^ This prevents passive penetration through the lipophilic and low-dielectric environment within the plasma membrane's lipid bilayer.^1,9^ Many current efforts are focused on designing cyclic peptides with increased bioavailability for drug development.

**Fig. 1:**
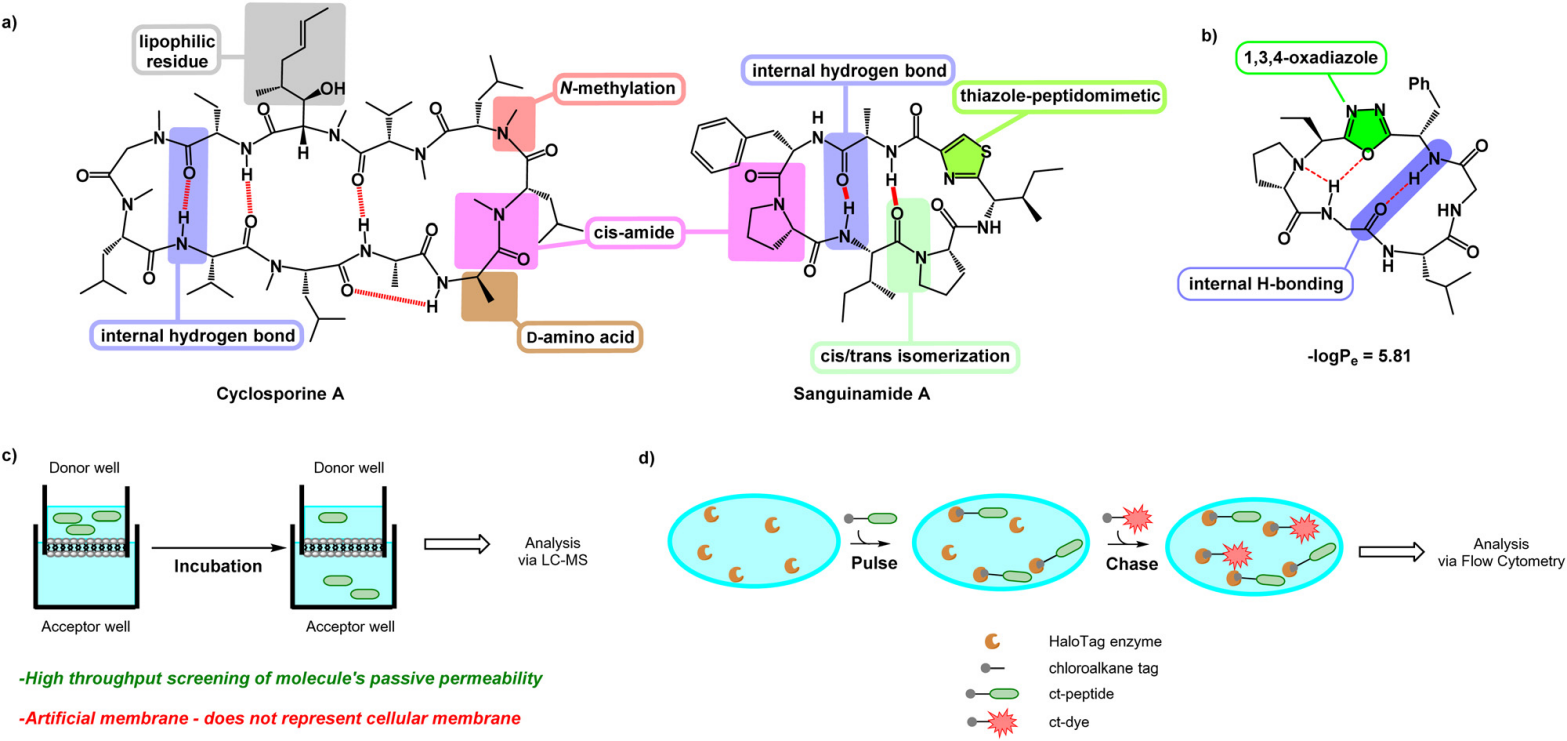
(a) Cyclosporine A and sanguinamide A. Various features enable these naturally occurring cyclic peptides to have high bioavailability. (b) Features of the oxadiazole-grafted (Odz) macrocycle may promote cell penetration. (c) Schematic showing PAMPA setup. A solution of peptide in donor well is separated from the acceptor well by an artificial membrane. After incubation, the degree to which a peptide can permeate through the artificial membrane and accumulate in the acceptor well is measured using LC-MS. (d) Schematic showing setup of the chloroalkane penetration assay (CAPA). Cells expressing HaloTag enzyme are pulsed with chloroalkane-tagged peptide (ct-peptide). Then, cells are chased with chloroalkane-tagged dye (ct-dye) which reacts with any unreacted HaloTag present after the pulse step. After washing, the cells are analysed using flow cytometry to measure the extent of labelling by the ct-dye, which is inversely proportional to the amount of ct-peptide that penetrated to the cytosol during the pulse step.

Despite their shortcomings, several naturally occurring cyclic peptides are known to be cell-penetrant, such as cyclosporine A and sanguinamide A (Fig. 1a).^10,11^ Although these molecules have a high number of polar residues and amide bonds, additional features such as *N*-methylation,^12^d-amino acids,^13^ lipophilic residues,^14^ and the ability to undergo internal hydrogen bonding^15^ contribute to their bioavailability. Cyclic peptides with heterocyclic functionalities embedded in the backbone, such as sanguinamide A, can also be unusually cell-penetrant. The replacement of an amide bond with a heterocyclic element may act similarly to *N*-methylation, decreasing the number of hydrogen bond donors and acceptors and thus lowering the polar surface area of the molecule.^11^ Incorporation of these features can make individual cyclic peptides or entire libraries more likely to be cell-penetrant.^16^

Yudin *et al.* investigated the use of (*N*-isocyanimino) triphenylphosphorane (PINC) multicomponent reaction in peptide macrocyclization to efficiently graft a 1,3,4-oxadiazole and an endocyclic amine in the cyclic backbone (Fig. 1b). This generates oxadiazole-grafted (Odz) macrocycles with high passive permeability as measured using the parallel artificial membrane permeability assay (PAMPA) (Fig. 1c). Additionally, Yudin *et al.* demonstrated increased lipophilicity with increased number of heterocycles in the macrocyclic backbone.^17^

Although PAMPA is an effective, reproducible, and high-throughput methodology for investigating passive permeation,^13,15,17–21^ it does not replicate the complexity of cellular membranes. Cell-based assays such as the Caco-2 assay and Madin–Darby canine kidney cell line (MDCK) permeability assay have been employed to offer a fuller assessment of transport *in vitro*.^22,23^ The chloroalkane penetration assay (CAPA) is a relatively recent methodology for measuring cell penetration in live cells (Fig. 1d).^24^ This assay uses cells that express a modified haloalkane dehalogenase known as HaloTag that reacts rapidly, selectively, and irreversibly with small chloroalkane ligands.^25,26^ CAPA measures cytosolic penetration by pulsing cells with a chloroalkane-tagged compound (ct-compound), then chasing with a chloroaklane-tagged dye (ct-dye). If the ct-compound penetrates the cell and escapes from endosomes, it reacts with HaloTag and blocks the reaction with the ct-dye in the subsequent chase step. Thus, in CAPA, cell penetration of the ct-molecule is inversely proportional to the signal from the ct-dye retained in the cells. The dose dependence of this signal can be fit to a sigmoidal curve, whose midpoint value (called the CP_50_) can be compared among ct-compounds as a quantitative comparison of cell penetration.^27,28^ CAPA is a cost-effective and high-throughput method for measuring the cell penetration of cyclic peptides and other molecules, and it establishes penetration to the cytosol without interference from material trapped in endosomes. In this work, we use CAPA to investigate the cell penetration of Odz macrocycles. We compare these results to PAMPA data, and use structural insights from NMR spectroscopy to better understand the cell penetration properties and mechanisms of this privileged macrocycle class.

## Results and discussion

### Design and synthesis of chloroalkane-tagged Odz macrocycles

**Fig. 2:**
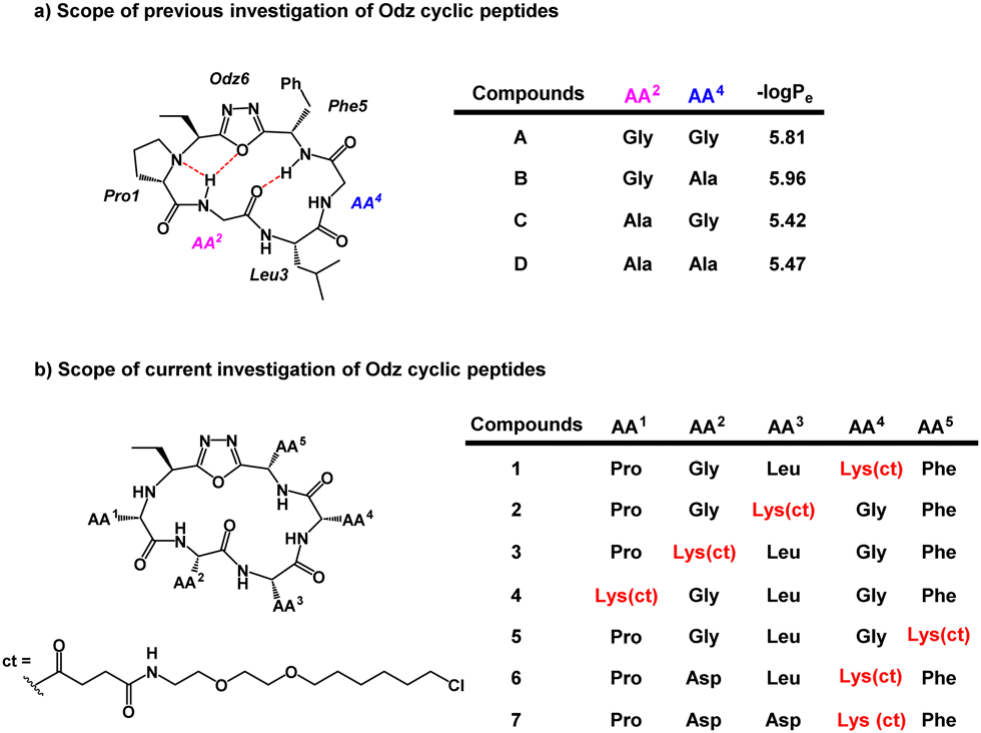
(a) Previous scope of the Odz cyclic peptides. (b) Scope of the chloroalkane-tagged Odz macrocycles. ct represents the chloroalkane carboxylic acid which underwent amide coupling with the lysine side chain. Lys(ct) refers to the lysine side chain that has chloroalkane-tag coupled to it.

Previous PAMPA investigation of Odz macrocycles (compounds A to D) reported high passive permeability (Fig. 2a).^29^ Upon changing the amino acid residue at the R^4^ site, little effect was observed on their permeability. This was correlated with negligible changes in permeability coefficient −log *P*_e_ between compounds A and B (−log *P*_e_ = 5.81 and 5.96, respectively) as well as between compounds C and D (−log *P*_e_ = 5.42 and 5.47, respectively). To install the chloroalkane tag on this Odz scaffold, a chloroalkane carboxylic acid functionality was coupled to an orthogonally deprotected lysine residue (Fig. 2b). To investigate how the position of the chloroalkane tag would affect cell penetration, the lysine to which the tag was attached was positioned at different sites around the macrocycle (compounds 1–5). Finally, to investigate how polar residues would affect cell penetration, aspartic acid (Asp) residues were added at the R^2^ and R^3^ sites (compounds 6 and 7). To synthesize the linear precursors, Fmoc-solid phase peptide synthesis on 2-chlorotrityl chloride resin was performed. Linear sequences were cyclized using the reagent PINC to afford the desired Odz macrocycles as previously described.^29,30^

### Cell penetration as measured by CAPA

**Fig. 3:**
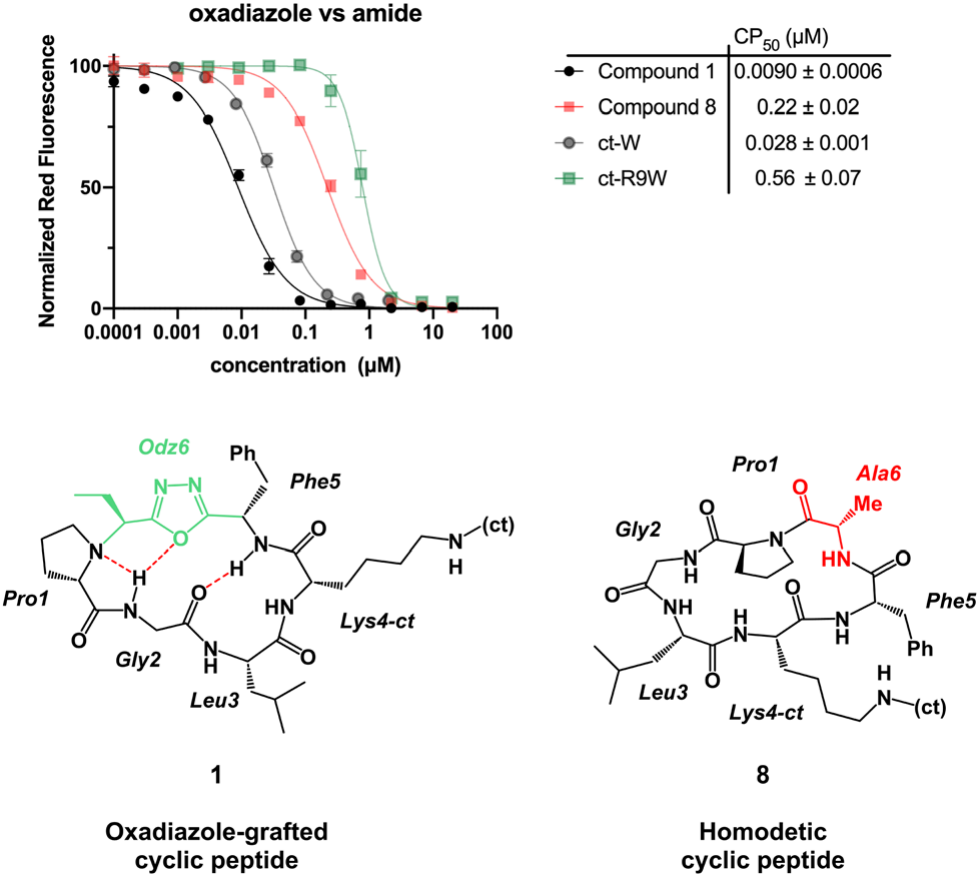
CAPA results comparing the Odz cyclic peptide (1), its homodetic counterpart (8), and control small molecule ct-W. CP_50_ value shown is after 4 hours of incubation; data for 30 min incubation are shown in Supporting Information. The structures highlight the key differences between cyclic peptides 1 and 8 and show the extra amide bond in the homodetic cyclic peptide. Depiction of compound 8 does not represent its solution conformation. Error bars show standard error of the mean from three independent trials. CP_50_ values and standard errors of the mean are derived from three independent curve fits from three independent trials.

With the macrocycles in hand, we sought to evaluate their cell penetration using CAPA (Table 1). Satisfyingly, compound 1 showed high cell penetration (CP_50_ after 30 min = 0.028 μM; CP_50_ after 4 h = 0.0090 μM) and outperformed both the small molecule control (ct-W: CP_50_ after 30 min = 0.25 μM; CP_50_ after 4 h = 0.028 μM) and a known polycationic cell-penetrating peptide (ct-R9W: CP_50_ after 30 min = 2.18 μM; CP_50_ after 4 h = 0.56 μM).^24,31^ Compound 8 was then prepared as a homodetic counterpart to 1, to examine the effects of the Odz group on cell penetration. Cell penetration of compound 8 was observed to be more than 20-fold worse than 1 (CP_50_ after 30 min = 1.0 μM; CP_50_ after 4 h = 0.22 μM, Fig. 3). These results match the PAMPA results for compound A and its homodetic counterpart, where the homodetic macrocycle had less passive permeability than the Odz cyclic peptide.^29^ In the prior study, it was concluded that the homodetic macrocycle had higher percentage of polar surface area because it had an additional amide bond. Additionally, the rigid β-turn conformation maintained by intramolecular H-bonding may be promoting higher passive permeability. For the CAPA data, a similar hypothesis can be used to rationalize the difference in cell penetration between the Odz macrocycle (1) and the homodetic control (8).

**Table 1:** Cell penetration and passive membrane permeability of Odz macrocycles as measured by CAPA and PAMPA, and relative hydrophobicity as measured by reverse-phase HPLC

Compounds	CP_50_ after 30 min incubation^a^ (μM)	CP_50_ after 4 h incubation^a^ (μM)	−log *P*_e_^b^	*T*_ret_ c (min)
1	0.028 ± 0.002	0.0090 ± 0.0006	7.77 ± 0.02	8.87
2	12.8 ± 2.4	2.2 ± 0.1	7.92 ± 0.07	8.00
3	0.48 ± 0.02	0.16 ± 0.01	7.84 ± 0.04	8.92
4	0.085 ± 0.005	0.028 ± 0.001	7.85 ± 0.052	8.63
5	0.51 ± 0.04	0.12 ± 0.003	>8	7.82
6	4.5 ± 0.6	1.3 ± 0.1	>8	8.70
7	9.0 ± 1.2	2.3 ± 0.2	>8	7.88
8	1.0 ± 0.03	0.22 ± 0.02	>8	7.95
ct-W	0.25 ± 0.01	0.028 ± 0.001	—	—
ct-R9W	2.18 ± 0.45	0.56 ± 0.07	—	—

a CAPA results were determined by incubating ct-peptides with cells in Opti-MEM for 30 min or 4 h prior to chasing with ct-dye.

b −log *P*_app_ values were determined using PAMPA with carbamazepine as an internal standard.

c Retention time values were determined using C18 LC-MS, gradient 95%/5% MeCN to 100% MeCN over 15 min. Errors represent standard error of the mean from three independent trials.

**Fig. 4:**
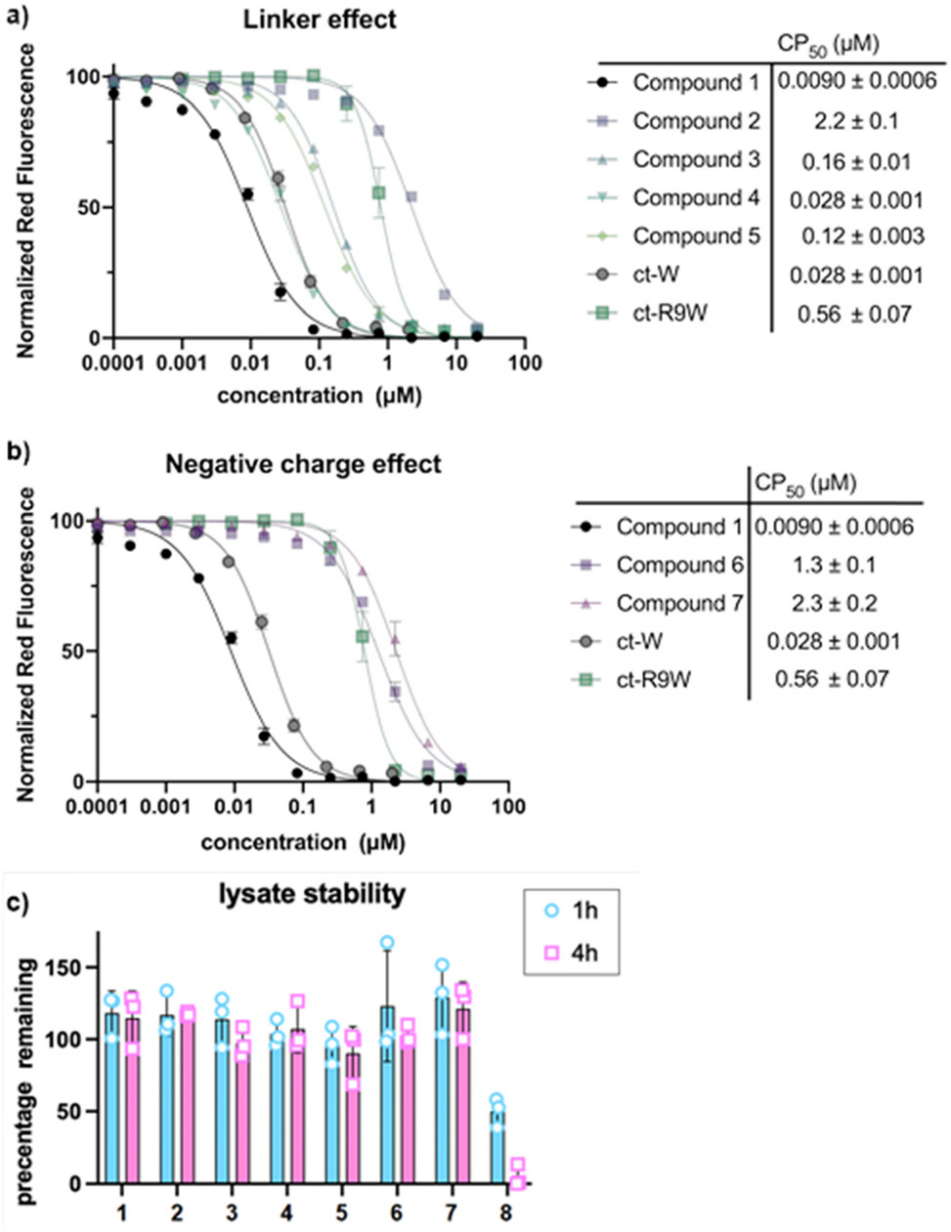
(a) CAPA results comparing the Odz macrocycle 1 and compounds which change the location of the chloroalkane tag (compounds 2, 3, and 5). CP_50_ value shown is after 4 hours of incubation; data for 30 min incubation are shown in Supporting Information. (b) CAPA results comparing the Odz cyclic peptide 1 and analogs with one or two Asp residues (6, 7). CP_50_ value shown is after 4 hours of incubation. (c) Lysate stability results showing no degradation for compounds 1–7 in a HeLa cell lysate after 4 hours of incubation. Error bars show standard error of the mean from three independent trials. CP_50_ values and standard errors of the mean are derived from three independent curve fits from three independent trials.

We then investigated how the position of the lysine and chloroalkane tag affected cell penetration (Fig. 4a). Previous investigation showed that Odz cyclic peptides maintained their rigid β-turn conformation despite changing the amino acid sequence, but different side chains led to different passive membrane permeabilities. More specifically, addition of phenylalanine (Phe) and leucine (Leu) at sites R^3^ and R^5^ increased the passive permeability of the cyclic peptides. This observation was explained by the ability of lipophilic residues to reduce solvent exposure of backbone amides. Incorporating Lys(ct) at the R^3^ or R^5^ position showed that the replacement of lipophilic residues such as Leu and Phe resulted in lower cell penetration (Compound 2: CP_50_ after 30 min = 12.8 μM; CP_50_ after 4 h = 2.2 μM. Compound 5: CP_50_ after 30 min = 0.51 μM; CP_50_ after 4 h = 0.12 μM). This drastic change in cell penetration upon removal of the lipophilic residues suggests that these sites are essential for efficient cell penetration, consistent with a role in binding the membrane, promoting cellular uptake, and/or promoting endosomal escape.

To provide additional evidence for this hypothesis, we used the retention time on reverse phase HPLC as a measure of the overall relative lipophilicity. A higher retention time suggests higher lipophilicity due to longer retention on the lipophilic C-18 stationary phase.^32^ Compounds 1 (*T*_ret_ = 8.87 min) and 4 (*T*_ret_ = 8.63 min) appear to be more lipophilic than compounds 2 (*T*_ret_ = 8.00 min) and 5 (*T*_ret_ = 7.82 min), suggesting that the removal of the hydrophobic residue changed the overall polar surface area of the cyclic peptide. Overall, the more lipophilic cyclic peptides had more efficient cell penetration than the more hydrophilic ones. However, compound 3 (*T*_ret_ = 8.92 min), which has a similar retention time to compound 1, does not exhibit similar cell penetrating ability (CP_50_ after 30 min = 0.48 μM; CP_50_ after 4 h = 0.16 μM).

An important control when performing CAPA is to check for degradation of chloroalkane-tagged compounds.^28^ Since it is known that exogeneously added material often becomes trapped in endosomes or in the lysosome, testing for degradation by cellular proteases ensures that the peptide is not degraded in an intracellular compartment, which would release a free chloroalkane and produce a false positive signal in CAPA. To measure susceptibility to degradation, ct-compounds were incubated in freshly prepared HeLa cell lysate and tested by HPLC after 1 and 4 hours to determine how much intact ct-compound remained. Previous work has shown this to be a stringent test for degradation.^33–35^ We observed that all the Odz macrocycles (compounds 1–7) had no significant degradation after 4 hours in HeLa lysate (Fig. 4c). This was a sharp contrast to the homodetic macrocycle 8, which had roughly 50% remaining after 1 hour and almost no intact macrocycle after 4 hours. This provides an important control for CAPA while also supporting that the Odz modification makes cyclic peptides more resistant to degradation by proteases and other enzymes.

Next, we incorporated polar residues to investigate the effect of increased polarity on cell penetration (Fig. 4b). Polar, hydrophilic amino acids are found in common receptor binding motifs like RGD^36^ and LDT.^37^ However, the addition of polar amino acids has shown to decrease the cell penetration of cyclic peptides.^38^ To show the wider applicability of using Odz macrocycles, we decided to incorporate Asp into the sequence. Unsurprisingly, compound 6 (CP_50_ after 30 min = 4.5 μM; CP_50_ after 4 h = 1.3 μM) had worse cell penetration than the initial hydrophobic compound 1. Adding a second Asp residue decreased cell penetration (compound 7, CP_50_ after 30 min = 9.0 μM; CP_50_ after 4 h = 2.3 μM). Poorer cell permeability was attributed to the negative charge and increased polar surface area.

### Passive membrane permeability as measured by PAMPA

While similar Odz macrocycles had previously been analyzed using PAMPA,^29^ we wanted to make a direct comparison to CAPA data by measuring passive membrane permeation of chloroalkane-tagged compounds 1–7. Analyzing the ct-compounds with PAMPA provided an interesting insight into the mechanism of cell uptake. PAMPA showed that all the chloroalkane-tagged Odz cyclic peptides had poor passive permeability (Table 1). Even compound 1, which had excellent cell penetration as measured by CAPA, had a −log *P*_e_ of 7.77 as measured by PAMPA. For comparison, the passively permeable macrocyclic standard CSA has a −log *P*_e_ value of 5.01.^11^ Comparing compound 1 and analogous compound A, which had much better passive permeation, it is likely that the incorporation of the chloroalkane tag affected the passive permeability due to the introduction of two additional exocyclic amide bonds.^39^ Since PAMPA measures passive permeation through an artificial membrane,^22^ the discrepancy between PAMPA and CAPA for compound 1 suggests that the cell penetration mechanism of 1 may not be through passive permeation but possibly through active transport.^13^ Overall, these results demonstrate how small changes in peptide structure can lead to a switch in cell penetration mechanism, even if the overall degree penetration does not appear to change much. These results also underscore CAPA's usefulness for investigating cyclic peptides that undergo cell penetration by means other than passive membrane permeation.

### Conformational analysis of the Odz macrocycles

**Fig. 5:**
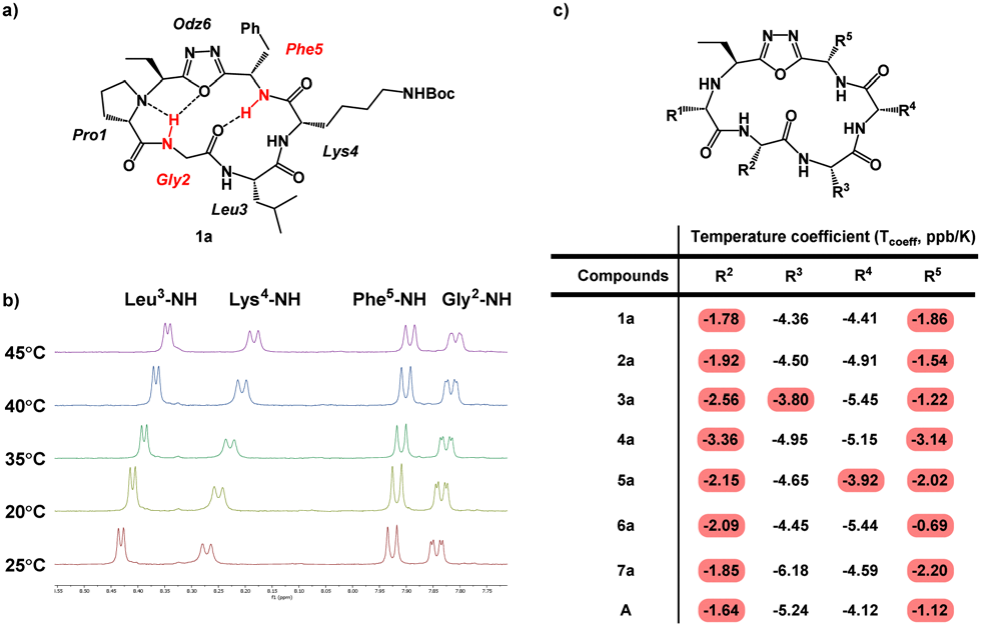
Variable-temperature NMR (VT NMR) of Odz macrocycles. (a) Structure of compound 1a is shown with specific backbone amide-NH that had low temperature coefficients (*T*_coeff_) shown in red. Based on their *T*_coeff_, the backbone amide NH groups of Phe^5^ and Gly^2^ are likely to be involved in intramolecular hydrogen bonding. (b) NMR spectra of compound 1a in DMSO-d_6_ showing the change in chemical shift as the sample is heated form 25 °C to 45 °C. (c) *T*_coeff_ values of compounds 1a to 7a and compound A. Backbone amides with *T*_coeff_ above −4 ppb K^−1^ were interpreted as participating in intramolecular hydrogen bonding, and backbone amides with *T*_coeff_ below −4 ppb K^−1^ were interpreted as being relatively solvent-exposed.^41^

Conformation is a prominent factor that affects relative exposure of hydrogen bond donors and acceptors, overall polar surface area, and ultimately extent of cell penetration. In prior work, the presence of a β-turn in hydrophobic peptides was shown to increase transcellular permeability as measured by Caco-2 assay.^40^ Representative compounds 1a–7a, in which the Lys residues are either protected by Boc or Dde, were used due to their ease of synthesis to perform NMR investigation for their respective ct-compounds 1 to 7 (Fig. 5). Compounds 1a–7a were dissolved in DMSO-d_6_ and COSY, TOCSY, ROESY, HSQC, and HMBC experiments were performed. These experiments were sufficient to fully assign spectra and to produce ROE-derived constraints for calculating solution structures. Additionally, variable temperature NMR (VT-NMR) was performed to elucidate which backbone amide protons were more solvent-exposed and which were likely to be internally hydrogen-bonded.

For all seven cyclic peptides, we observed similar patterns in the VT-NMR data (Fig. 5c). Specifically, the amide protons of residues 2 and 5 showed little change in the chemical shift as the sample was heated (Fig. 5). This suggests that these amide NH groups are participating in intramolecular hydrogen bonding. Compounds 1a and 4a showed temperature coefficient (*T*_coeff_) values at R^3^ and R^4^ sites (3a: *T*_coeff_ R^3^ = −3.80; 5a: *T*_coeff_ R^3^ = −3.92) that are close to the cut off value of 4 ppb K^−1^.^41^ Relative solvent exposure of these amide-NH groups is ambiguous. The consistency of the R^2^–NH and R^5^–NH having low *T*_coeff_ values would suggest that the cyclic peptides may all have similar conformations. Additionally, the backbone amides that have low *T*_coeff_ are consistent with the β-turn structure from previously reported Odz macrocycle A.^29^ This suggests that the Odz cyclic peptides maintain their rigid β-turn structure even with the incorporation of the chloroalkane tag and that the placement of the tag around the backbone has little to no effect on the turn structure of the Odz cyclic peptides.

**Fig. 6:**
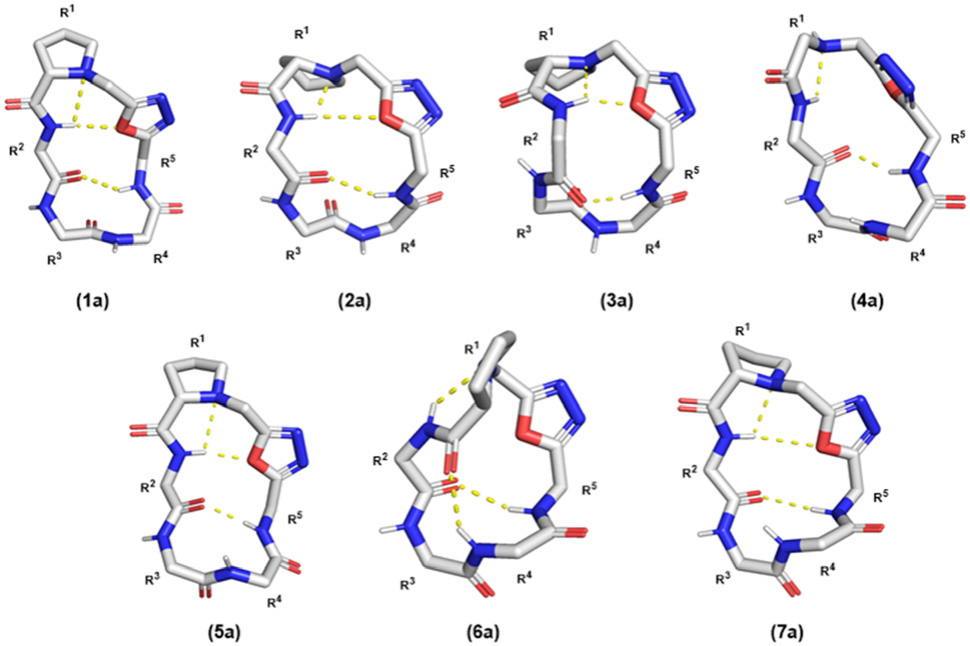
Solution structures of compound 1a to 7a in DMSO-d_6_. Side chains are omitted for clarity.

Solution structures calculated based on ROE-derived constraints provided additional evidence that the β-turn structure originally observed for compound A was maintained between R^5^–NH and R^2^–C=O for all Odz cyclic peptides (Fig. 6).^29,30^ The structures also revealed hydrogen bonding between R^2^–NH and the endocyclic amine of Pro^1^ for all seven macrocycles, again consistent with previous observations for macrocycle A. This conclusion is in accordance with the VT-NMR results. Interestingly, different conformations were observed for 4a and 6a, characterized by a lack of H-bonding interactions between the R^2^–NH and the oxygen of the oxadiazole. For compound 4a, which lacks proline at position 1, it is likely that diminished rigidity around the turn structure increases overall conformational freedom. Yet, for compound 6a, the lack of interaction between R^2^–NH and oxadiazole was observed despite the presence of proline in position 1. We noted that the calculated solution structure of compound 6a had many violations of ROE-derived distance restraints, suggesting that multiple interconverting conformations are present. This result suggests that substitution of an Asp in position 2 not only introduces polarity, but also significantly decreases the overall degree of rigidity for compound 6a. Compound 7a, which has an additional Asp residue in position 3, has a greater degree of structure compared to compound 6a, with fewer restraint violations and an overall structure more similar to the other Odz macrocycles. These non-additive effects are common for small macrocycles, and further highlight the complexities of interpreting data for simple substitution series within systems that are so sensitive to conformation.

The aforementioned structural insights are highly useful, but ultimately do not provide definitive answers as to how specific conformations control passive *versus* active cell penetration mechanisms for Odz macrocycles. Several investigations have focused on the correlation between passive permeability and conformation of cyclic peptides.^20,40,42^ However, the unique observations among passively penetrant compound A, cell-penetrant but not passively penetrant compound 1, and less penetrant compounds 2–7 are striking and provide useful guidelines for further development of Odz macrocycles and similar cyclic peptides. Further investigation is underway to understand how transport mechanism is related to the properties and conformations of these and related cyclic peptides.

## Conclusions

We have demonstrated cell-penetrating ability of Odz macrocycles using CAPA. We have also shown that changing the site of the choloralkane tag results in differences in cell penetration. The addition of negatively charged residues impeded cell penetration. PAMPA, which measures passive permeability, showed poor passive permeability for chloroalkane-tagged Odz cyclic peptides. On the other hand, CAPA, which measures cell penetration in living cells, showed high penetration for 1 and moderate penetration for other Odz macrocycles. We conclude that an active transport mechanism may be important for cell penetration of some Odz macrocycles, especially those with larger polar surface area and/or solvent-exposed hydrogen bond donors. Conformational study demonstrated that Odz cyclic peptides maintain the unique β-turn structure observed for related macrocycles, and that individual substitutions had hard-to-predict, non-additive effects on conformation and cell penetration. These findings underscore the complicated factors involved in designing cyclic peptides for cell penetration. Moreover, a focus on passive permeability at early stages of drug design may result in overlooking compounds with considerable cell penetration through other mechanisms.

## Conflicts of interest

There are no conflicts to declare.

## Supplementary Material

CB-005-D3CB00201B-s001
