# Supplementary material for: Cell penetration of oxadiazole-containing macrocycles
Source: RSC Chem Biol. 2024 Jan 15;5(4):328–34. doi: 10.1039/d3cb00201b (PMC10989506; doi:10.1039/d3cb00201b)
Supplement: CB-005-D3CB00201B-s001 [file CB-005-D3CB00201B-s001.pdf]

Electronic Supplementary Material (ESI) for RSC Chemical Biology.
This journal is © The Royal Society of Chemistry 2023

Supporting information for:

Cell penetration of oxadiazole-containing macrocycles

Sungjoon Huh,[1,] Nefeli Batistatou,[2] Jing Wang,[2] Joshua Kritzer,[2,*] and Andrei Yudin[1,*]

1. Davenport Research Laboratories, University of Toronto, 80 St. George St, Toronto, Ontario, M5S 3H6, Canada

2. Department of Chemistry, Tufts University, 62 Talbot Ave, Medford, MA 02155, USA

†These authors contributed equally to this work.

Table of Contents

1 General Information 3

2 Preparation of chloroalkane oxadiazole-grafted macrocycles 4

2.1 General procedure for solid phase peptide synthesis 4

2.1.1 General preparation A: synthesis of oxadiazole-grafted macrocycle 5

2.1.2 General preparation B: Incorporation of chloroalkane tag 15

3 Chloroalkane Penetration Assay (CAPA) 21

4 Lysate stability assay method 21

5 Parallel Artificial Membrane Permeability Assay (PAMPA) 22

6 Variable temperature NMR 23

7 Structure Determination 26

7.1 Conformation and distance restraint 27

8 NMR spectra 31

9 Variable NMR spectra 61

1 General Information

All reagents were utilized as received from commercial sources unless otherwise noted. All solvents were of reagent grade quality and freshly distilled prior to use. Dichloromethane (DCM) was distilled over CaH_2 under an atmosphere of nitrogen. Tetrahydrofuran was distilled over Na with benzophenone indicator under an atmosphere of nitrogen. **Chromatography:** Flash-column chromatography was performed using Merck silica gel 60 (40-63 µm). Thin-layer chromatography (TLC) was performed using Merck silica gel 60 F 254 plates, with UV (254 nm) detection followed by $KMnO_4$ or ninhydrin stain. **NMR Spectrometry:** All NMR spectra were recorded on either a Bruker DPX300, Bruker AV 300, Bruker AV 400 at 300 K or a Varian 600 Unity spectrometer at 299 K. ^1H NMR spectra chemical shifts (δ) are reported in parts per million (ppm) referenced to residual protonated solvent peak (DMSO-d_6 δ = 2.50, $CDCl_3$ δ = 7.26). Spectral data is reported as follows: chemical shift, multiplicity (s = singlet, d = doublet, t = triplet, q = quartet, dd = doublet of doublets, dt = doublet of triplets, ddt = doublet of doublet of triplets, dtd = doublet of triplet of doublets, m = multiplet, br = broad, h = heptet, dddd = doublet of doublet of doublet of doublets, qd = quartet of doublets, td = triplet of doublets, tt = triplet of triplets), coupling constant (J) in Hertz (Hz), and integration. ^{13}C NMR spectra chemical shifts (δ) are reported in parts per million (ppm) and were referenced to carbon resonances in the NMR solvent (DMSO-d_6 δ = 39.5, $CDCl_3$ δ = 77.2). **Mass Spectrometry:** High-resolution mass spectrometry (HRMS) ESI (m/z) spectra were recorded on a Bruker MicroTof or an Orbitrap LTQ XL (Nanospray) of Thermo Scientific. At the University of Toronto high resolution mass spectra were obtained on a VG 70-250S (double focusing) mass spectrometer at 70 eV or on an ABI/Sciex Qstar mass spectrometer with ESI source, MS/MS and accurate mass capabilities or on JEOL AccuTOF-DART instrument. **RP-HPLC/MS:** Low-resolution mass spectra (ESI) were collected on an Agilent Technologies 1200 series HPLC paired to a 6130 Mass Spectrometer. Compounds were resolved on Phenomenex's Kinetex 2.6u C18 50x4.6mm column at room temperature with a flow of 1 mL/min. The gradient consisted of eluents A (0.1% formic acid in double distilled water) and B (0.1% formic acid in HPLC-grade acetonitrile). *Method A*: A linear gradient starting from 5% of B to 95% over 4 min at a flow rate of 1.0 mL/min. Stays constant at 95% for 1 min and then returns to 5% over 0.5 min. *Method B*: Stays constant at 5% of B for 0 min at a flow rate of 1.5 mL/min, followed by a linear gradient to 95% over 4.0 min. Stays constant at 95% of B for 3.0 min and then returns to 5% B over 0.5 min. *Method C*: A linear gradient starting from 5% of B to 95% over 15 min at a flow rate

of 1.0 mL/min. Stays constant at 95% for 1 min and then returns to 5% over 0.5 min. **Previously Reported Compounds:** The following compounds were prepared according to literature procedures: N-(isocyanoimino)triphenylphosphorane (PINC),[1] N-α-Fmoc-N-ε-1-(dimethyl-2,6-dioxocyclohex-1-ylidene)-*L*-lysine (Fmoc-Lys(DDE)-OH),[2] 4-((2-(2-((6-chlorohexyl)oxy)ethoxy)ethyl)amino)-4-oxobutanoic acid (chloroalkane tag, ct).[3]

2 Preparation of chloroalkane oxadiazole-grafted macrocycles

2.1 General procedure for solid phase peptide synthesis

Regeneration of resin:[4] 2-chlorotrityl chloride (2-CTC) resin was loaded into a round bottom flask with no magnetic stir bar and suspended in DCM. Thionyl chloride (3 eq.) and pyridine (6 eq.) was added into the mixture and refluxed for 2 hrs. The resin was filtered after the regeneration and dried under vacuum. The dried 2-CTC resin was then stored under Ar until use for Fmoc-SPPS. **Preparation of resin**: Fmoc amino acid (1.5 eq. with respect to resin) was dissolved in DCM (10 mL/g resin). DIPEA was added (1.5 eq. with respect to the amino acid) and the solution was sonicated until fully dissolved. The 2-chlorotrityl resin was allowed to swell in DCM (5 mL/g of resin) for 15 min. The DCM was then drained. The Fmoc amino acid solution was added to the vessel containing the 2-CTC resin and the vessel was agitated for 5 min. Another 1.5 eq. of DIPEA was then added and the vessel was left to agitate for an additional 60 min. The resin was then treated with methanol (1 mL/g of resin) to endcap any remaining reactive chloro groups on the 2-CTC resin. The solution was mixed for 15 min, drained, and then rinsed with DCM (x3), DMF (x3), DCM (x2), and MeOH (x3). The resin was then used towards preparation of a linear peptide. **Fmoc deprotection:** The resin was treated with 20% piperidine in DMF twice, for 10 and 20 min respectively, with consecutive DMF and DCM washes after each addition. **Fmoc amino acid coupling:** The resin was treated with 3 eq. of Fmoc amino acid, 3 eq. of HATU and 6 eq. of DIPEA in DMF for 60 min. For difficult couplings, a second treatment with 3 eq. of Fmoc amino acid, 3 eq. of HATU and 6 eq. of DIPEA in DMF for 40 min was employed. **Cleavage from the solid support:** Once the desired linear sequence was synthesized, the resin was treated with 30% hexafluoroisopropanol (HFIP) in DCM, two times for 20 min each, to afford cleavage from the solid support. Linear precursor was then triturated with cold Et_2O (10 mL) and centrifuged down. Supernatant was removed using a Pasteur pipette and dried down under a vacuum.

2.1.1 General preparation A: synthesis of oxadiazole-grafted macrocycle

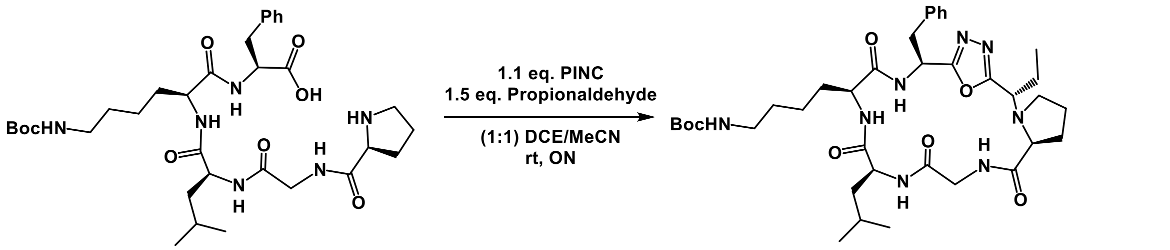

The linear peptide (1 eq.) was suspended in a mixture of (1:1) dichloroethane:acetonitrile (0.05 M). Propionaldehyde (1.5 eq.) and (N-isocyanimino)triphenylphosphorane (PINC) (1 eq.) were added to the reaction mixture. Reaction mixture was stirred overnight at room temperature. Cyclization was monitored by RP-HPLC and additional PINC (0.5 eq.) and propionaldehyde (0.5 eq.) was added if reaction is incomplete after 16 hrs. Once the starting linear precursor was consumed, the mixture was concentrated under a stream of N_2. Reverse-Phase C18 column chromatography was performed to separate the two diastereomer (*S*-ethyl and *R*-ethyl). The desired product fraction was pooled and lyophilized. MD simulations indicated the major diastereomer was the *S*-ethyl isomer.

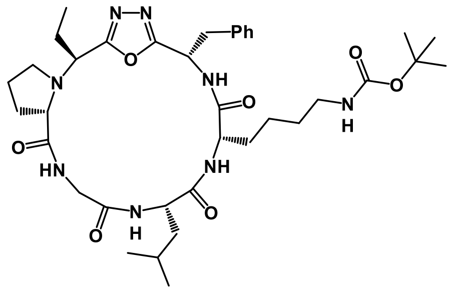

cyclo[PGLK(Boc)F] odz/ethyl (1) was prepared using general preparation A. *S*-ethyl isomer: White solid, 50 mg, 23%. *R*-ethyl isomer: White solid, 3.3 mg, 1.5%. 15:1 mixture of *S*-isomer and *R*-ethyl isomer. Retention time of *S*-ethyl = 8.869 min (Method C). Retention time of *R*-ethyl = 8.977 min (Method C). *S*-ethyl **^{1}H NMR (500 MHz, DMSO-d_6)** δ 8.43 (d, *J* = 4.8 Hz, 1H, NH-Leu), 8.27 (d, *J* = 8.0 Hz, 1H, NH-Lys), 7.93 (d, *J* = 8.5 Hz, 1H, NH-Phe), 7.84 (dd, *J* = 8.6, 2.7 Hz, 1H, NH-Gly), 7.27 – 7.16 (m, 3H, ArCH-Phe), 7.13 – 7.07 (m, 2H, ArCH-Phe), 6.75 (t, *J* = 5.6 Hz, 1H, NH-Boc), 5.41 (dt, *J* = 8.5, 6.4 Hz, 1H, αCH-Phe), 4.20 (dd, *J* = 16.9, 8.6 Hz, 1H, αCH'-Gly), 3.93 – 3.78 (m, αCH-Ethyl, αCH-Leu, αCH-Lys), 3.66 (dd, *J* = 17.0, 2.6 Hz, 1H, αCH"-Gly), 3.57 (dd, *J* = 10.1, 3.2 Hz, 1H, αCH-Pro), 3.15 (dd, *J* = 13.6, 6.4 Hz, 1H, βCH'-Phe), 2.99 (dd, *J* = 13.6, 8.5 Hz, 1H, βCH"-Phe), 2.87 (q, *J* = 6.8 Hz, 2H, εCH_2-Lys), 2.74 (t, *J* = 7.7 Hz, 1H, δCH'-Pro), 2.56 (dt, *J* = 9.0, 6.0 Hz, 1H, δCH"-Pro), 2.12 – 2.01 (m, 2H, βCH'-Pro, βCH'-Ethyl), 1.82 – 1.59 (m, 7H, γCH_2-Pro, βCH"-Pro, βCH"-Ethyl, βCH_2-Lys, γCH-Leu), 1.58 – 1.44 (m, 2H, βCH_2-Leu), 1.37 (s, 9H, $(CH_3)_3$-Boc), 1.34 – 1.28 (m, 2H, δCH_2-

Lys), 1.25 – 1.09 (m, 2H, γCH_2-Lys), 0.89 (dd, J = 33.9, 6.5 Hz, 6H, $(CH_3)_2$ -Leu), 0.63 (t, J = 7.3 Hz, 3H, γCH_3-Ethyl). *S*-ethyl **^{13}C NMR (126 MHz, DMSO-*d*$_6$)** δ 174.2, 171.9, 171.4, 170.1, 167.0, 164.6, 155.6, 136.1, 129.1, 128.3, 126.8, 77.3, 61.0, 60.1, 54.1, 53.2, 53.0, 47.1, 41.0, 30.7, 29.5, 29.0, 28.3, 24.2, 23.7, 23.2, 22.7, 22.6, 21.7, 10.7. **HRMS (DART-TOF)** $[M+H]^+$ *m/z* calculated for $C_{37}H_{57}N_8O_7$ = 725.4345, *m/z* found = 725.43371.

HPLC trace of Compound 1a-*S*-Ethyl

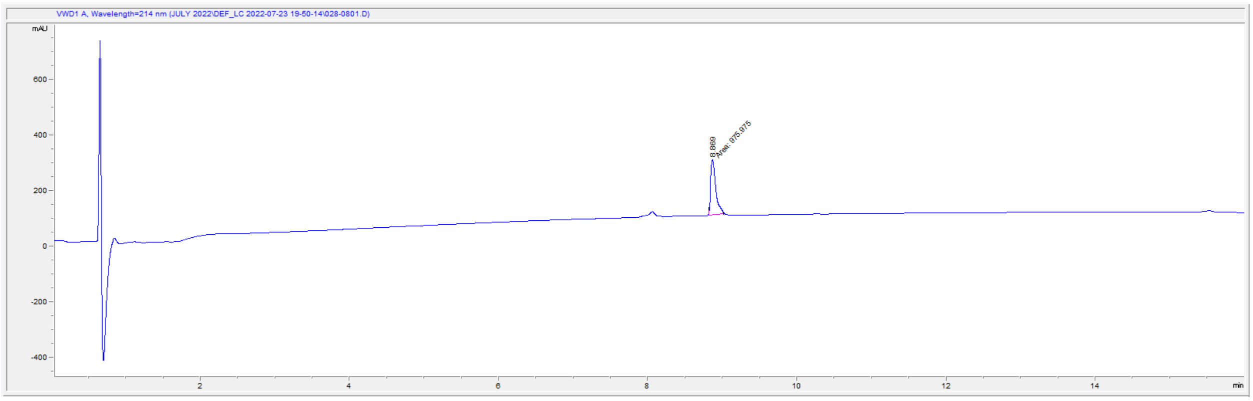

HPLC trace of Compound 1a-*R*-Ethyl

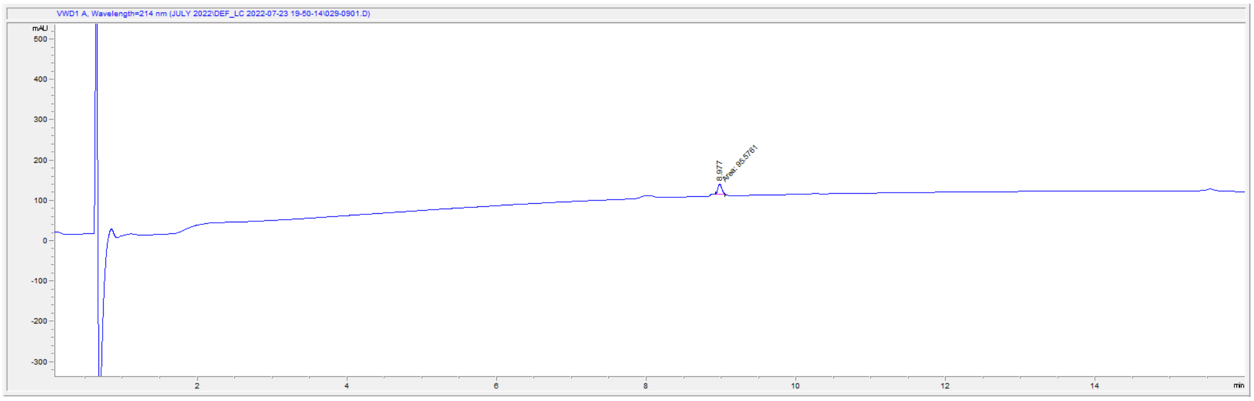

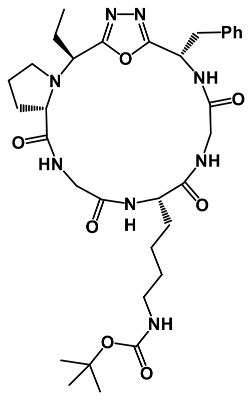

cyclo[PGK(Boc)GF] odz/ethyl (2) was prepared using general preparation A. *S*-ethyl isomer: White solid, 27 mg, 12%. *R*-ethyl isomer: White solid, 6.5 mg, 2.9%. 4.2:1 mixture of *S*-isomer and *R*-ethyl isomer. Retention time of *S*-ethyl = 8.621 min (Method C). Retention time of *R*-ethyl = 8.977 min (Method C). *S*-ethyl **^{1}H NMR (500 MHz, DMSO-*d*$_6$)** δ 8.82 (dd, *J* = 7.2, 5.2 Hz, 1H, NH-Gly4), 8.62 (d, *J* = 4.6 Hz, 1H, NH-Lys), 8.01 (d, *J* = 9.0 Hz, 1H, NH-Phe), 7.87 (dd, *J* = 7.4, 3.1 Hz, 1H, NH-Gly2), 7.26 – 7.15 (m, 3H, ArCH-Phe), 7.13 – 7.08 (m, 2H, ArCH-Phe), 6.76 (t, *J* = 5.8 Hz, 1H, NH-Boc), 5.50 (dt, *J* = 9.0, 7.2 Hz, 1H, αCH-Phe), 4.11 (dd, *J* = 17.1, 7.5 Hz, 1H, αCH'-Gly2), 3.98 (dd, *J* = 17.0, 7.3 Hz, 1H, αCH'-Gly4), 3.88 (dd, *J* = 11.2, 4.2 Hz, 1H, αCH-Ethyl), 3.79 – 3.71 (m, 1H, αCH-Lys), 3.59 (dd, *J* = 17.1, 3.0 Hz, 1H, αCH"-Gly2), 3.52 (dd, *J* = 10.1, 3.2 Hz, 1H αCH-Pro), 3.42 (dd, *J* = 17.0, 5.0 Hz, 1H, αCH"-Gly4), 3.12 – 3.00 (m, 2H, βCH_2-Phe), 2.89 (q, *J* = 6.6 Hz, 2H, εCH_2-Lys), 2.77 (t, *J* = 7.7 Hz, 1H, δCH'-Pro), 2.57 (dt, *J* = 9.0, 5.9 Hz, 1H, δCH"-Pro), 2.14 – 2.00 (m, 1H, βCH'-Pro), 1.92 – 1.81 (m, 1H, βCH'-Ethyl), 1.81 – 1.63 (m, 6H, βCH"-Pro, βCH"-Ethyl, βCH_2-Lys, γCH_2-Pro), 1.40 – 1.34 (m, 11H, $(CH_3)_3$-Boc, δCH_2-Lys), 1.32 – 1.15 (m, 2H, γCH_2-Lys), 0.63 (t, *J* = 7.3 Hz, 3H, γCH_3-Ethyl). *S*-ethyl **^{13}C NMR (126 MHz, DMSO-*d*$_6$)** δ 174.3, 172.0, 170.0, 169.1, 167.0, 164.7, 155.6, 136.0, 129.2, 128.2, 126.7, 77.3, 60.9, 59.8, 54.7, 52.8, 46.4, 42.6, 41.2, 30.9, 29.5, 29.2, 28.3, 23.7, 22.8, 22.2, 10.7. **HRMS (DART-TOF)** $[M+H]^+$ *m/z* calculated for $C_{33}H_{49}N_8O_7$ = 669.3719, *m/z* found = 669.3723.

HPLC trace of Compound 2a-*S*-Ethyl

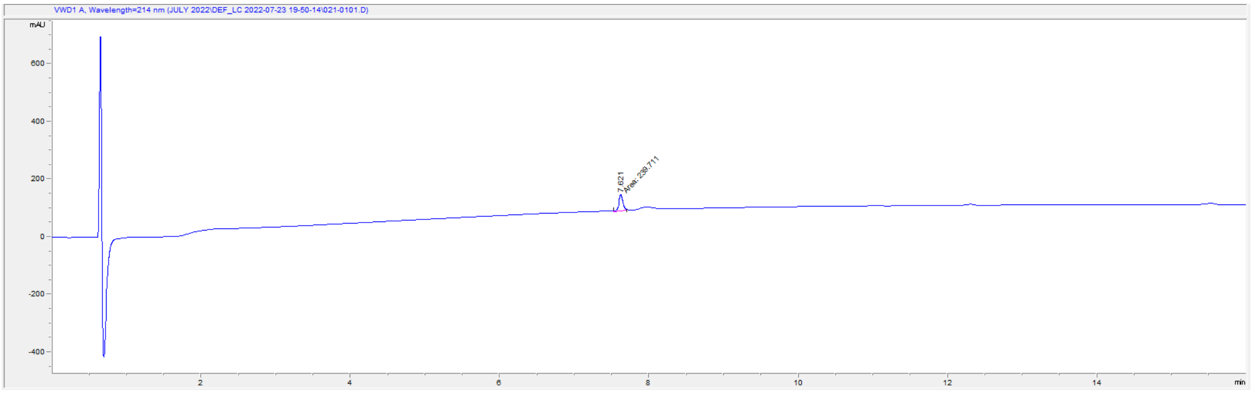

HPLC trace of Compound 2a-*R*-Ethyl

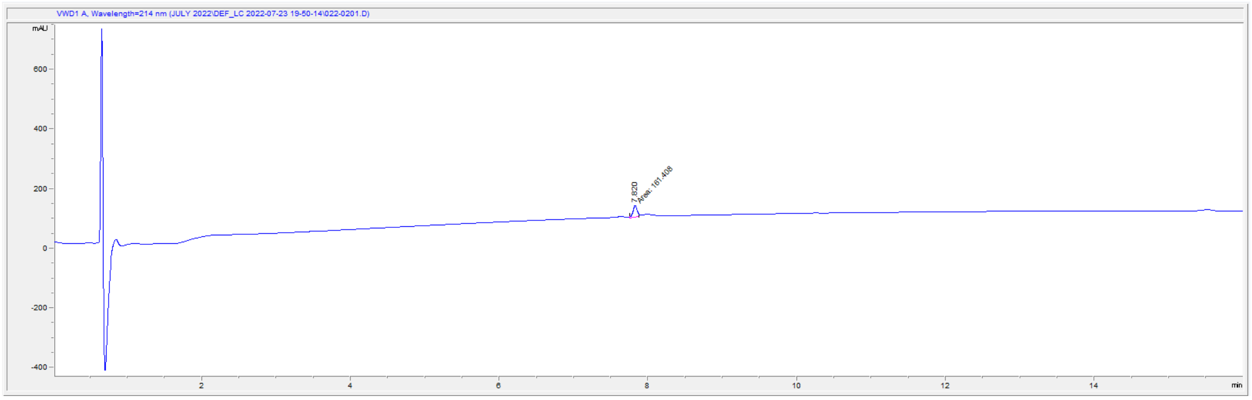

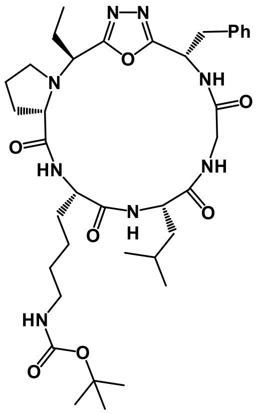

cyclo[PK(Boc)LGF] odz/ethyl (3) was prepared using general preparation A. *S*-ethyl isomer: White solid, 48 mg, 22%. *R*-ethyl isomer: White solid, 22 mg, 9.9%. 2.2:1 mixture of *S*-isomer and *R*-ethyl isomer. Retention time of *S*-ethyl = 9.081 min (Method C). Retention time of *R*-ethyl = 9.305 min (Method C). *S*-ethyl **^{1}H NMR (500 MHz, DMSO-*d*$_6$)** δ 8.76 – 8.72 (m, 1H, NH-Gly), 8.71 (d, *J* = 3.7 Hz, 1H, NH-Leu), 8.27 (d, *J* = 9.4 Hz, 1H, NH-Phe), 8.21 (d, *J* = 8.6 Hz, 1H, NH-Lys), 7.25 – 7.15 (m, 3H, ArCH-Phe), 7.11 – 7.05 (m, 2H, ArCH-Phe), 6.71 (t, *J* = 5.7 Hz, 1H, NH-Boc), 5.52 (q, *J* = 8.6 Hz, 1H, αCH-Phe), 4.48 (dt, *J* = 7.8, 4.7 Hz, 1H, αCH-Lys), 4.02 (dd, *J* = 17.1, 7.8 Hz, 1H, αCH'-Gly), 3.88 (dd, *J* = 11.5, 4.0 Hz, 1H, αCH-Ethyl), 3.84 – 3.76 (m, 1H, αCH-Leu), 3.64 (dd, *J* = 10.4, 2.6 Hz, 1H, αCH-Pro), 3.43 – 3.36 (m, 1H, αCH"-Gly), 3.07 (dd, *J* = 13.4, 7.1 Hz, 1H, βCH'-Phe), 2.97 (dd, *J* = 13.4, 8.9 Hz, 1H, βCH"-Phe), 2.90 – 2.77 (m, 2H, εCH$_2$-Lys), 2.70 (t, *J* = 7.6 Hz, 1H, δCH'-Pro), 2.60 – 2.52 (m, 1H, δCH"-Pro), 2.21 – 2.06 (m, 1H, βCH'-Pro), 1.90 – 1.49 (m, 10H, βCH"-Pro, γCH-Leu, βCH$_2$-Leu, γCH$_2$-Pro, βCH$_2$-Ethyl, βCH$_2$-Lys), 1.45 – 1.36 (m, 2H, γCH$_2$-Lys), 1.34 (s, 9H, CH$_3$)$_3$-Boc), 1.30 – 1.22 (m, 2H, δCH$_2$-Lys), 0.96 – 0.84 (m, 6H, (CH$_3$)$_2$ -Leu), 0.63 (t, *J* = 7.3 Hz, 3H, γCH$_3$-Ethyl). *S*-ethyl **^{13}C NMR (126 MHz, DMSO-*d*$_6$)** δ 174.4, 172.9, 172.4, 169.4, 167.9, 164.9, 155.9, 136.3, 129.3, 128.9, 127.3, 77.8, 61.0, 60.7, 54.0, 53.9, 51.0, 46.7, 42.9, 33.9, 31.7, 29.5, 28.6, 24.6, 24.6, 23.1, 22.9, 22.5, 22.2, 11.1. **HRMS (DART-TOF)** $[M+H]^+$ *m/z* calculated for $C_{37}H_{57}N_8O_7$ = 725.4345, *m/z* found = 725.4335.

HPLC trace of Compound 3a-*S*-Ethyl

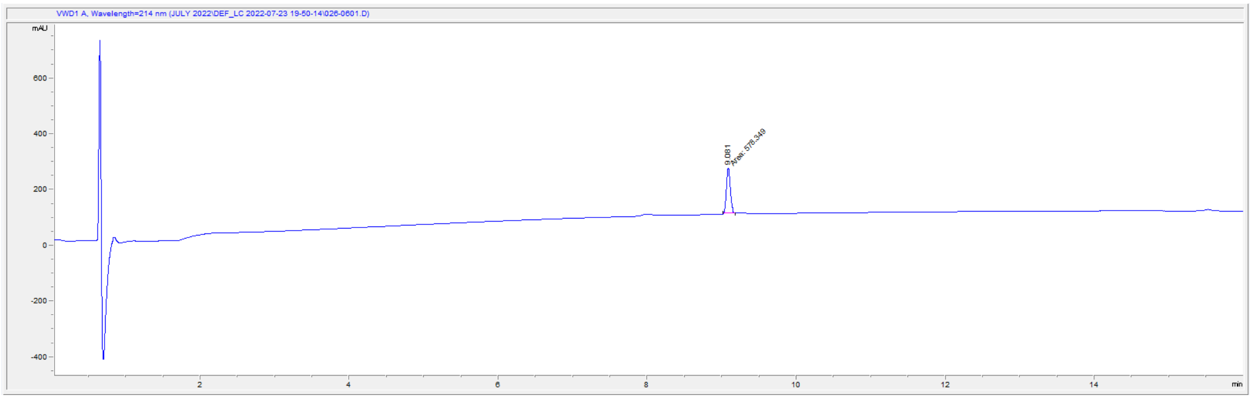

HPLC trace of Compound 3a-*R*-Ethyl

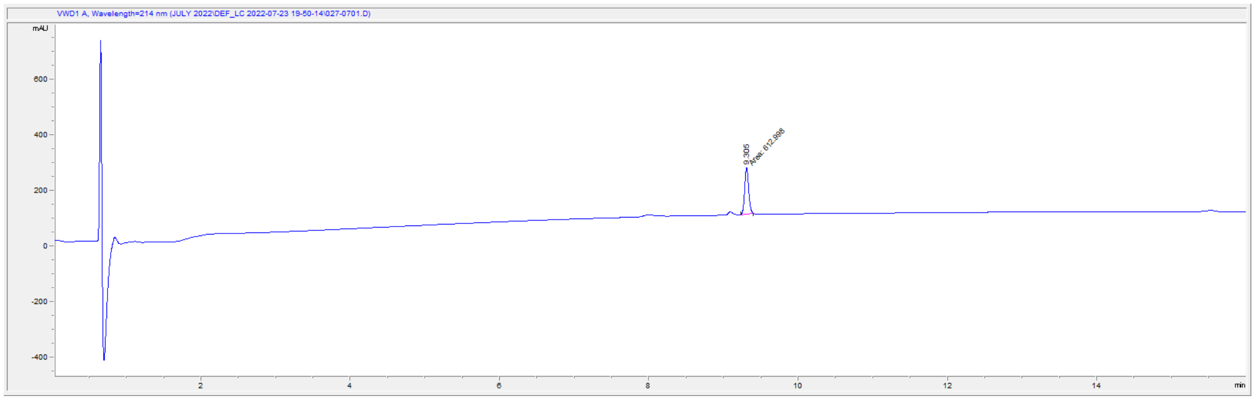

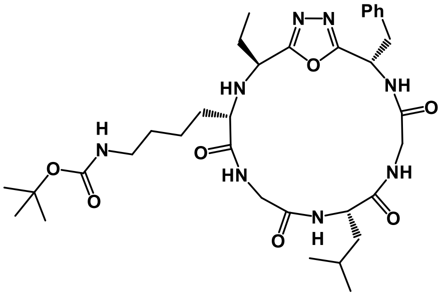

cyclo[K(Boc)GLGF] odz/ethyl (4) was prepared using general preparation A. White solid, 17 mg, 7.7%. Retention time of *S*-ethyl = 8.599 min (Method C). *S*-ethyl **^{1}H NMR (500 MHz, DMSO-d_6)** δ 8.65 (d, *J* = 4.9 Hz, 1H, NH-Leu), 8.60 (dd, *J* = 7.6, 5.0 Hz, 1H, NH-Gly4), 8.15 (d, *J* = 8.7 Hz, 1H, NH-Phe), 8.01 (dd, *J* = 7.0, 4.2 Hz, 1H, NH-Gly2), 7.29 – 7.15 (m, 5H, ArCH-Phe), 6.78 (t, *J* = 5.7 Hz, 1H, NH-Boc), 5.44 (dt, *J* = 8.7, 6.4 Hz, 1H, αCH-Phe), 4.07 – 3.95 (m, 2H, αCH'-Gly2, αCH'-Gly4), 3.85 – 3.77 (m, 1H, αCH-Leu), 3.74 – 3.70 (m, 1H, αCH-Ethyl), 3.64 (dd, *J* = 17.1, 4.1 Hz, 1H, αCH"-Gly2), 3.40 (dd, *J* = 17.0, 4.9 Hz, 1H, αCH"-Gly4), 3.12 (dd, *J* = 13.6, 8.7 Hz, 1H, βCH'-Phe), 3.03 (dd, *J* = 13.5, 6.3 Hz, 1H, βCH"-Phe), 2.99 – 2.94 (m, 1H, αCH-Lys), 2.93 – 2.87 (m, 2H, εCH$_2$-Lys), 2.73 (s, 1H, NH-Ethyl), 1.92 – 1.72 (m, 2H, βCH$_2$-Ethyl), 1.72 – 1.45 (m, 5H, βCH$_2$-Lys, βCH$_2$-Leu, γCH-Leu), 1.40 – 1.33 (m, 13H, (CH$_3$)$_3$-Boc, γCH$_2$-Lys, δCH$_2$-Lys), 0.88 (dd, *J* = 25.9, 5.9 Hz, 6H, (CH$_3$)$_2$ -Leu), 0.74

(t, J = 7.4 Hz, 3H, γCH_3-Ethyl). *S*-ethyl **^{13}C NMR (126 MHz, DMSO-*d*$_6$)** δ 173.6, 172.0, 170.2, 168.7, 167.5, 165.0, 155.6, 136.3, 129.2, 128.3, 126.6, 77.3, 60.8, 55.8, 52.9, 46.3, 42.5, 41.3, 38.6, 38.4, 32.9, 29.5, 28.3, 25.5, 24.2, 22.73 21.9. **HRMS (DART-TOF)** $[M+H]^+$ *m/z* calculated for $C_{34}H_{53}N_8O_7$ = 685.4032, *m/z* found = 685.4028.

HPLC trace of Compound 4a-*S*-Ethyl

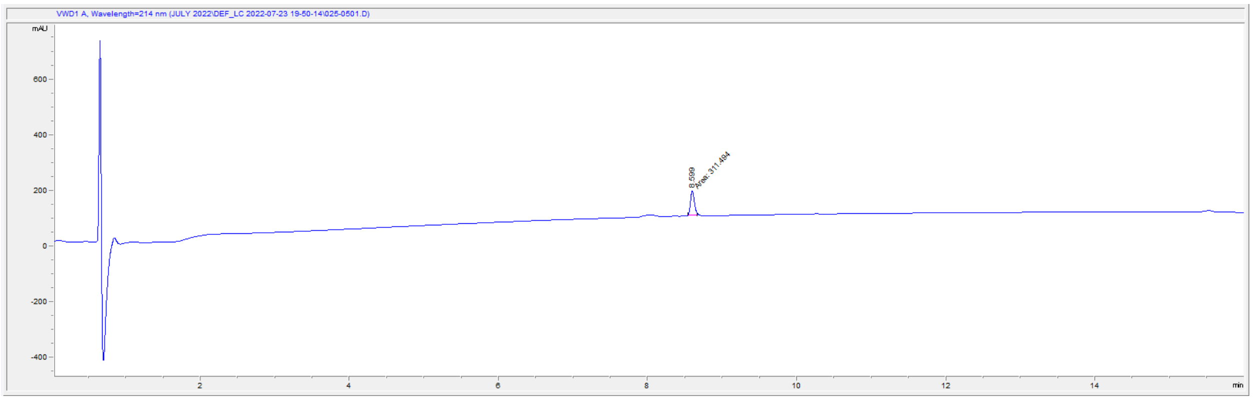

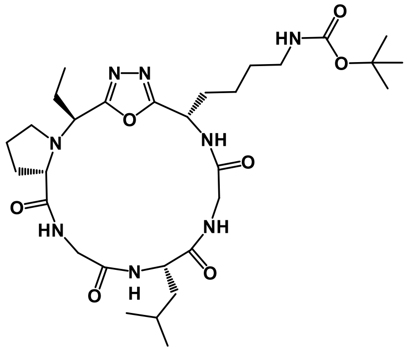

cyclo[PGLGK(Boc)] odz/ethyl (5) was prepared using general preparation A. *S*-ethyl isomer: White solid, 28 mg, 13%. *R*-ethyl isomer: White solid, 6.9 mg, 3.1%. 4.0:1 mixture of *S*-ethyl isomer and *R*-ethyl isomer. Retention time of *S*-ethyl = 7.493 min (Method C). Retention time of *R*-ethyl = 7.619 min (Method C). *S*-ethyl **^{1}H NMR (500 MHz, DMSO-*d*$_6$)** δ 8.72 (d, J = 5.4 Hz, 1H, NH-Leu), 8.59 (dd, J = 7.2, 5.3 Hz, 1H, NH-Gly4), 8.07 (t, J = 4.6 Hz, 1H, NH-Gly2), 7.93 (d, J = 9.2 Hz, 1H, NH-Lys), 6.72 (t, J = 5.7 Hz, 1H, NH-Boc), 5.21 (q, J = 8.0 Hz, 1H, αCH-Lys), 4.01 – 3.95 (m, 2H, αCH-Ethyl, αCH'-Gly2), 3.93 (d, J = 7.1 Hz, 1H, αCH'-Gly4), 3.79 (dt, J = 9.1, 5.6 Hz, 1H, αCH-Leu), 3.64 – 3.52 (m, 2H, αCH-Pro, αCH"-Gly2), 3.41 (dd, J = 17.0, 5.1 Hz, 1H, αCH"-Gly4), 2.92 – 2.81 (m, 3H, δCH'-Pro, εCH_2-Lys), 2.72 – 2.63 (m, 1H, δCH"-Pro), 2.08 – 1.99 (m, 1H, βCH'-Pro), 1.85 – 1.79 (m, 2H, βCH_2-Ethyl), 1.78 – 1.47 (m, 8H, βCH_2-Lys, βCH_2-Leu, γCH-Leu, γCH_2-Pro, βCH"-Pro), 1.36 (s, 9H, $(CH_3)_3$-Boc), 1.30 – 1.10 (m, 2H, γCH_2-Lys), 0.86 (dd, J = 19.1, 6.3 Hz, 6H, $(CH_3)_2$ -Leu), 0.69 (t, J = 7.3 Hz, 3H, γCH_3-Ethyl). *S*-ethyl **^{13}C NMR (126 MHz, DMSO-*d*$_6$)** δ 174.6, 172.0, 170.1, 168.8, 166.5, 165.7, 155.5, 77.3, 60.7, 59.3, 52.9, 52.5, 44.2, 42.8, 38.4, 32.4, 31.1, 28.9, 28.3, 24.2, 23.8, 22.9, 21.9, 21.7, 21.4, 10.7**.** **HRMS (DART-TOF)** $[M+H]^+$ *m/z* calculated for $C_{30}H_{51}N_8O_7$ = 635.3875, *m/z* found = 635.3868.

HPLC trace of Compound 5a-*S*-Ethyl

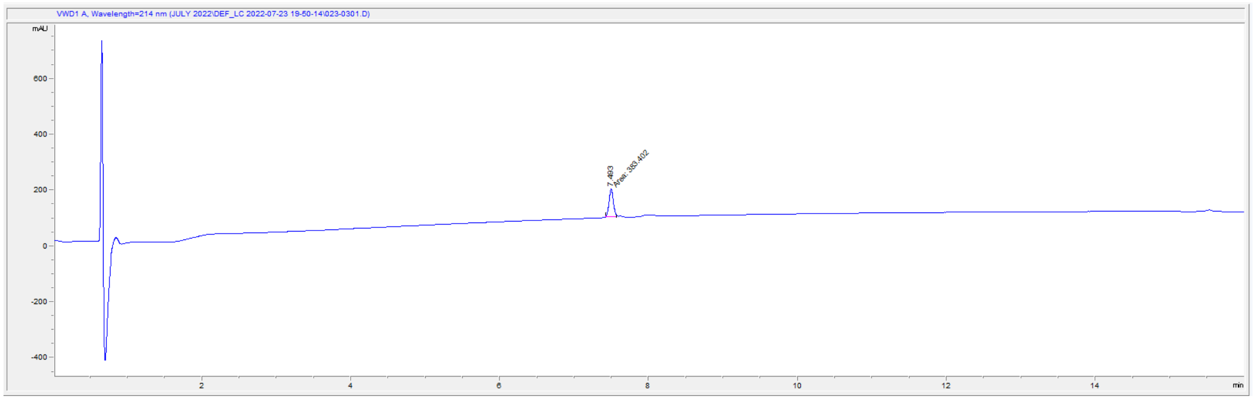

HPLC trace of Compound 5a-*R*-Ethyl

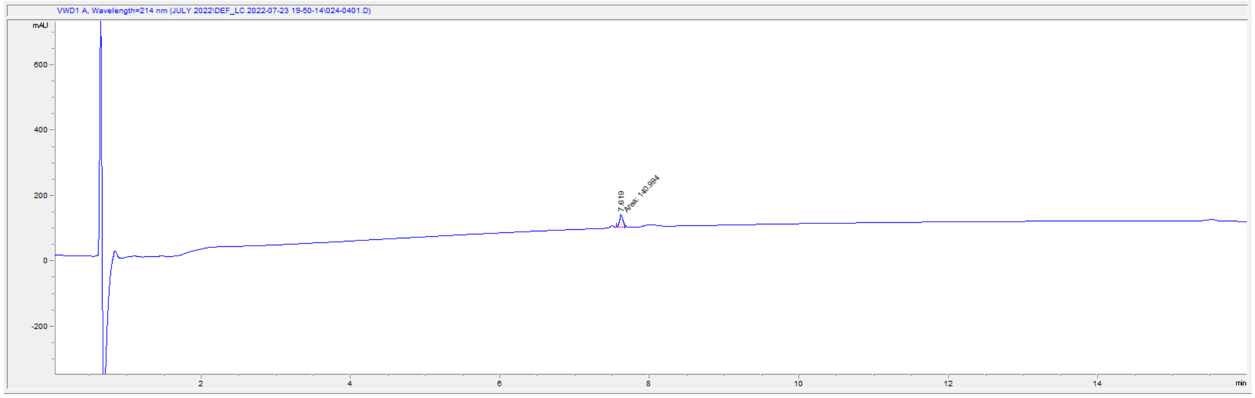

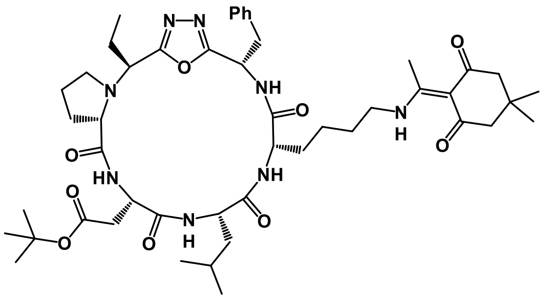

cyclo[PD(tBu)LK(Dde)F] odz/ethyl (6) was prepared using general preparation A. *S*-ethyl isomer: White solid, 14 mg, 8.4%. *R*-ethyl isomer: White solid, 2.9 mg, 1.8%. 4.7:1 mixture of *S*-isomer and *R*-ethyl isomer. Retention time of *S*-ethyl = 10.486 min (Method C). Retention time of *R*-ethyl = 10.631 min (Method C). *S*-ethyl **^{1}H NMR (500 MHz, DMSO-d_6)** δ 13.25 (t, *J* = 5.3 Hz, 1H, NH-DDE), 8.74 (d, *J* = 4.7 Hz, 1H, NH-Leu), 8.25 (d, *J* = 9.2 Hz, 1H, NH-Asp), 8.18 (d, *J* = 7.5 Hz, 1H, NH-Lys), 7.94 (d, *J* = 8.7 Hz, 1H, NH-Phe), 7.25 – 7.16 (m, 3H, ArCH-Phe), 7.14 – 7.03 (m, 2H, ArCH-Phe), 5.41 (dt, *J* = 9.3, 6.6 Hz, 1H, αCH-Phe), 4.74 (dt, *J* = 9.3, 6.9 Hz, 1H, αCH-Asp), 3.89 – 3.80 (m, 2H, αCH-Ethyl, αCH-Lys), 3.73 (dt, *J* = 10.0, 5.0 Hz, 1H, αCH-Leu), 3.63 (dd, *J* = 10.5, 2.7 Hz, 1H, αCH-Pro), 3.39 (q, *J* = 6.6 Hz, 2H, εCH_2-Lys), 3.20 (dd, *J* = 13.4, 6.5 Hz, 1H, βCH'-Phe), 3.11 (dd, *J* = 13.4, 9.9 Hz, 1H, βCH"-Phe), 2.82 (dd, *J* = 15.8, 5.3 Hz, 1H, βCH'-Asp), 2.72 – 2.65

(m, 2H, βCH”-Asp, δCH’-Pro), 2.59 – 2.51 (m, 1H, δCH”-Pro), 2.47 (s, 3H, CH_3-DDE), 2.25 (s, 4H, CH_2-DDE), 2.17 – 2.05 (m, 1H, βCH’-Ethyl), 1.99 – 1.88 (m, 1H, βCH”-Ethyl), 1.87 – 1.62 (m, 8H, βCH_2-Lys, βCH_2-Leu, βCH_2-Pro, γCH_2-Pro), 1.58 – 1.43 (m, 3H, γCH-Leu, δCH_2-Lys), 1.38 (s, 9H, $(CH_3)_3$-tBu-Asp), 1.35 – 1.22 (m, 2H, δCH_2-Lys), 0.94 – 0.83 (m, 12H, $(CH_3)_2$-DDE, $(CH_3)_2$-Leu), 0.60 (t, J = 7.3 Hz, 3H, γCH_3-Ethyl). *S*-ethyl **^{13}C NMR (126 MHz, DMSO-*d*$_6$)** δ 173.7, 172.8, 171.4, 171.3, 170.8, 169.2, 167.2, 164.4, 136.0, 128.8, 128.4, 126.9, 106.9, 80.5, 60.5, 60.2, 53.9, 53.7, 53.6, 52.3, 47.9, 47.0, 42.6, 38.5, 31.0, 29.7, 28.6, 28.0, 27.9, 27.6, 24.2, 23.8, 23.4, 23.0, 22.7, 21.5, 17.3, 10.6. **HRMS (ESI)** $[M+H]^+$ *m/z* calculated for $C_{48}H_{71}N_8O_9$ = 903.5339, *m/z* found = 903.5334.

HPLC trace of Compound 6a-*S*-Ethyl

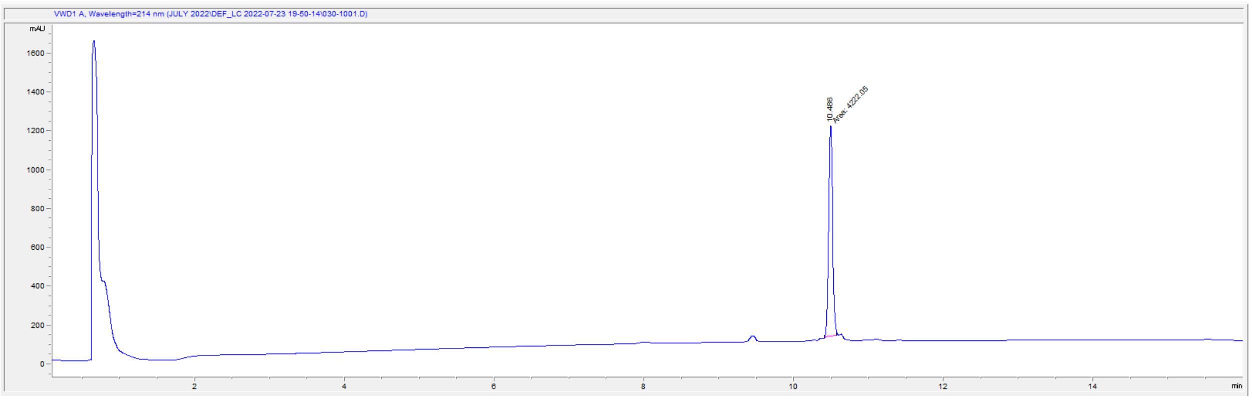

HPLC trace of Compound 6a-*R*-Ethyl

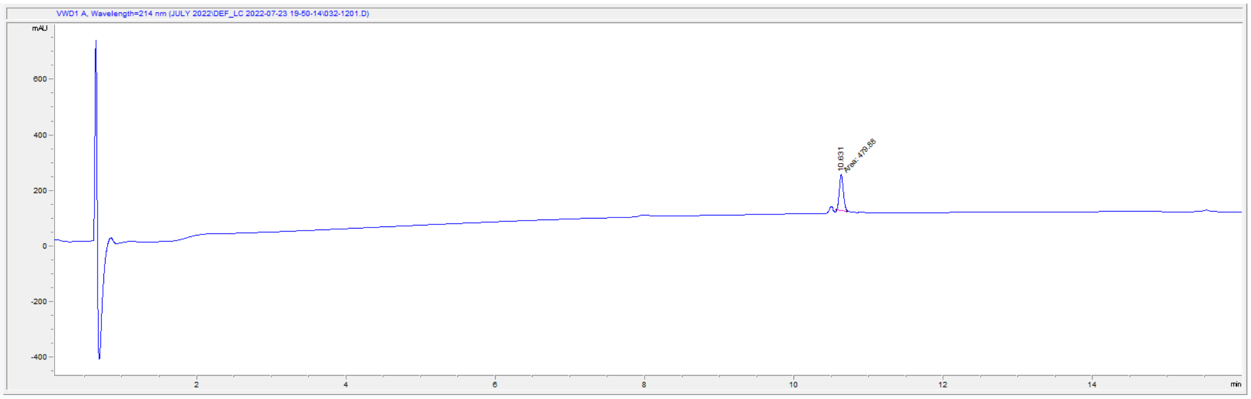

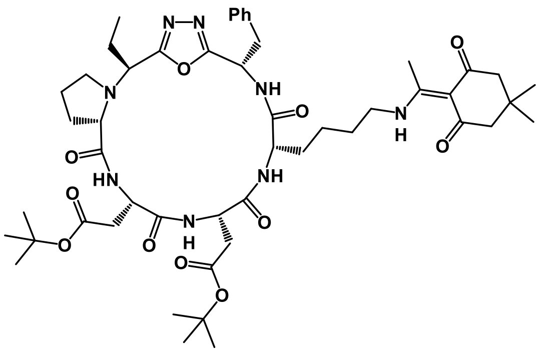

cyclo[PD(tBu)D(tBu)K(Dde)F] odz/ethyl (7) was prepared using general preparation A. *S*-ethyl isomer: White solid, 29 mg, 18%. *R*-ethyl isomer: White solid, 5.6 mg, 3.5%. 5.1:1 mixture of *S*-isomer and *R*-ethyl isomer. Retention time of *S*-ethyl = 10.973 min (Method C). Retention time of *R*-ethyl = 11.129 min (Method C). *S*-ethyl **^{1}H NMR (500 MHz, DMSO-*d*$_6$)** δ 13.25 (t, *J* = 5.2 Hz, 1H, NH-DDE), 9.15 (d, *J* = 6.1 Hz, 1H, NH-Asp3), 8.16 (dd, *J* = 7.8, 6.1 Hz, 2H, NH-Asp2, NH-Lys), 7.98 (d, *J* = 8.9 Hz, 1H, NH-Phe), 7.25 – 7.15 (m, 3H, ArCH-Phe), 7.14 – 7.10 (m, 2H, ArCH-Phe), 5.37 (dt, *J* = 9.0, 7.4 Hz, 1H, αCH-Phe), 4.64 (dt, *J* = 7.9, 5.4 Hz, 1H, αCH-Asp2), 4.04 (ddd, *J* = 10.0, 6.1, 4.4 Hz, 1H, αCH-Asp3), 3.81 (dd, *J* = 10.2, 5.4 Hz, 1H, αCH-Ethyl), 3.80 – 3.74 (m, 1H, αCH-Lys), 3.56 (dd, *J* = 10.5, 3.1 Hz, 1H, αCH-Pro), 3.37 (p, *J* = 6.5 Hz, 2H, εCH_2-Lys), 3.26 – 3.13 (m, 2H, βCH_2-Phe), 3.00 (dd, *J* = 16.8, 4.3 Hz, 1H, βCH'-Asp3), 2.78 (dd, *J* = 16.8, 9.4 Hz, 1H, δCH'-Pro), 2.71 – 2.66 (m, 2H, βCH"-Asp3, δCH"-Pro), 2.60 – 2.50 (m, 2H, βCH_2-Asp2), 2.45 (s, 3H, CH_3-DDE), 2.25 (s, 4H, CH_2-DDE), 2.16 – 2.03 (m, 1H, βCH'-Pro), 1.97 – 1.81 (m, 2H, βCH_2-Lys), 1.80 – 1.66 (m, 5H, βCH"-Pro, γCH_2-Pro, βCH_2-Ethyl), 1.60 – 1.44 (m, 2H, δCH_2-Lys), 1.42 (s, 9H, $(CH_3)_3$-tBu-Asp2), 1.36 (s, 9H, $(CH_3)_3$-tBu-Asp3), 1.34 – 1.19 (m, 2H, γCH_2-Lys), 0.92 (s, 6H, $(CH_3)_2$-DDE), 0.53 (t, *J* = 7.3 Hz, 3H, γCH_3-Ethyl). *S*-ethyl **^{13}C NMR (126 MHz, DMSO-*d*$_6$)** δ 174.0, 172.7, 170.8, 170.6, 169.8, 169.3, 168.8, 166.8, 164.9, 136.1, 129.0, 128.4, 126.9, 106.9, 80.4, 61.0, 60.3, 53.8, 53.6, 52.3, 51.4, 48.5, 46.7, 42.4, 38.7, 35.3, 31.0, 29.7, 28.1, 27.9, 27.8, 27.7, 27.6, 23.7, 23.0, 22.9, 17.3, 10.4. **HRMS (DART-TOF)** $[M+H]^+$ *m/z* calculated for $C_{50}H_{74}N_8O_{11}$ = 961.5393, *m/z* found = 961.5389.

HPLC trace of Compound 7a-*S*-Ethyl

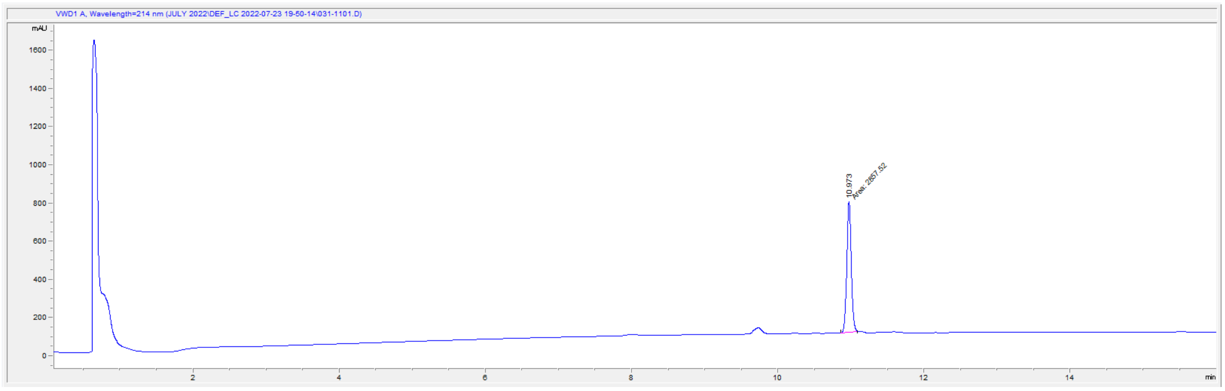

HPLC trace of Compound 7a-*R*-Ethyl

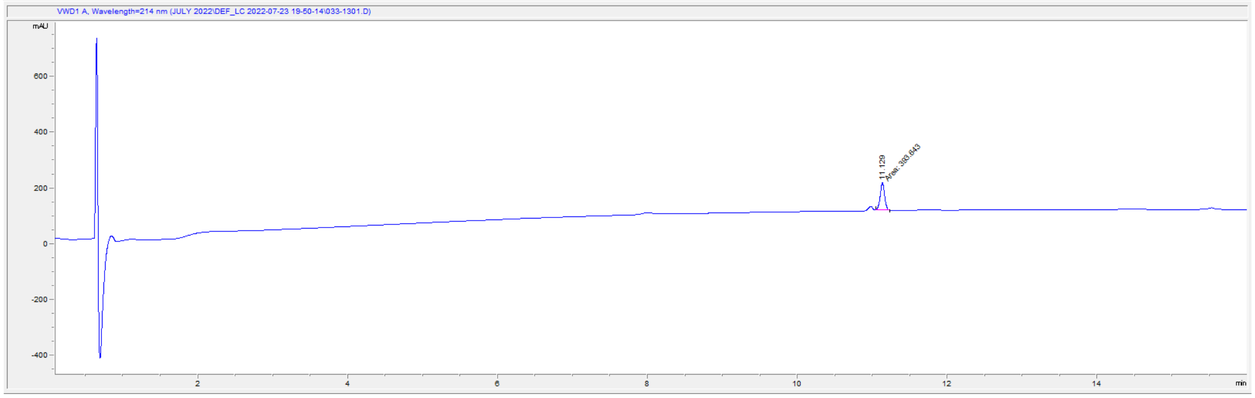

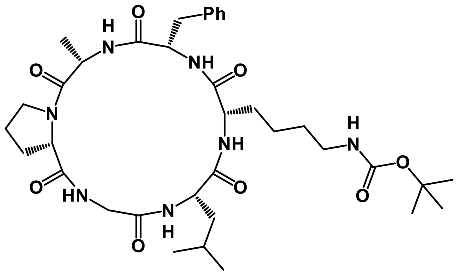

cyclo[PGLK(Boc)FA] odz/ethyl (8) In a 9-dram vial, 0.4-0.8 mmol of the linear peptide and DEPBT (1.5 eq.) were dissolved in 20-40 mL of freshly distilled THF (0.02 M). DIPEA (3 eq.) was then added to the solution. If the resulting solution was cloudy DMF was added dropwise while stirring until a homogeneous mixture persisted. The reaction mixture was left to stir overnight at room temperature (16 h). Tetraalkylammonium MP-carbonate resin (6 eq.) was then added to the reaction mixture and stirring was continued for an additional 24 h. The reaction was then filtered through a solid-phase extraction vessel and rinsed with DCM (2 mL). The crude filtrate was concentrated *in vacuo* and purified by reverse-phase C18 flash column chromatography. White solid, 20 mg, 16%. Retention time = 7.711 min (Method C). ^{1}H NMR of major conformer – NH-amide and CHα were only reported **^{1}H NMR (500 MHz, DMSO-*d*$_6$)** δ 8.87 (dd, *J* = 8.2, 5.3 Hz, 2H), 8.73 (s, 1H), 8.01 (d, *J* = 5.6 Hz, 1H), 7.93 (t, *J* = 9.0 Hz, 2H), 7.14 (d, *J* = 8.1 Hz, 1H), 6.75 (t, *J* = 5.5 Hz, 1H), 4.41 (d, *J* = 7.9 Hz, 2H), 4.32 (qd, *J* = 7.1, 2.7 Hz, 1H), 4.19 (dd, *J* = 16.5, 9.1 Hz, 1H), 3.98 (dd, *J* = 16.8, 8.8 Hz, 1H), 3.82 (dt, *J* = 9.5, 5.7 Hz, 1H), 3.66 (dd, *J* = 16.4, 3.5 Hz, 1H), 2.89 (dd, *J* = 7.6, 6.1 Hz, 1H), 2.69 (dd, *J* = 13.6, 7.4 Hz, 1H). ^{1}H NMR of minor conformer – NH-amide and CHα were only reported **^{1}H NMR (500 MHz, DMSO-*d*$_6$)** δ 4.55 (p, *J* = 6.8 Hz, 1H), 4.49 – 4.44 (m, 1H), 3.92 (dd, *J* = 17.2, 7.9 Hz, 1H), 3.86 (dd, *J* = 15.5, 7.9 Hz, 1H), 3.27 (d, *J* = 10.5 Hz, 1H), 8.87 (dd, *J* = 8.2, 5.3 Hz, 2H), 7.93 (t, *J* = 9.0 Hz, 2H), 7.85 (d, *J* = 7.3 Hz, 1H), 7.53 (d, *J* = 7.0 Hz, 1H), 6.75 (t, *J* = 5.5 Hz, 1H). **HRMS (ESI)** $[M+H]^+$ *m/z* calculated for $C_{36}H_{56}N_7O_8$ = 714.4185, *m/z* found = 714.4180.

HPLC trace of Compound 8a

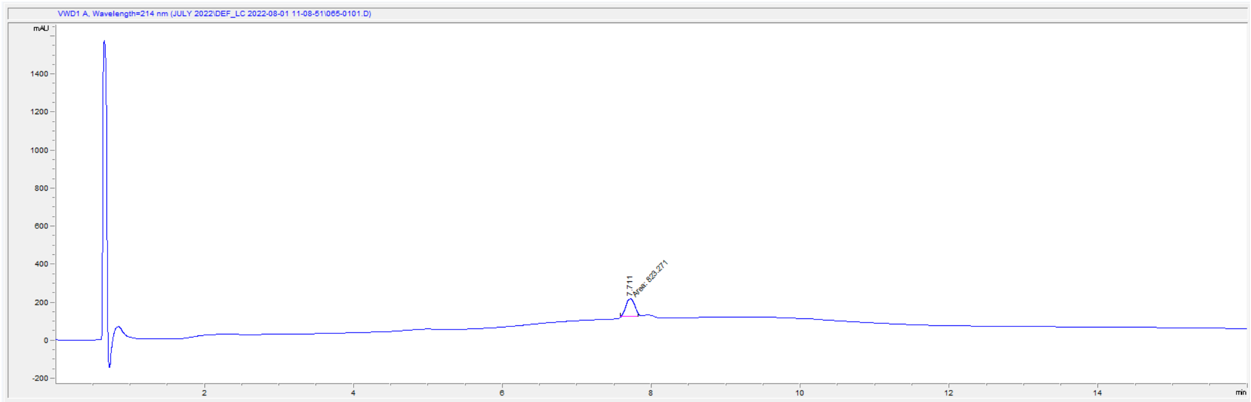

2.1.2 General preparation B: Incorporation of chloroalkane tag

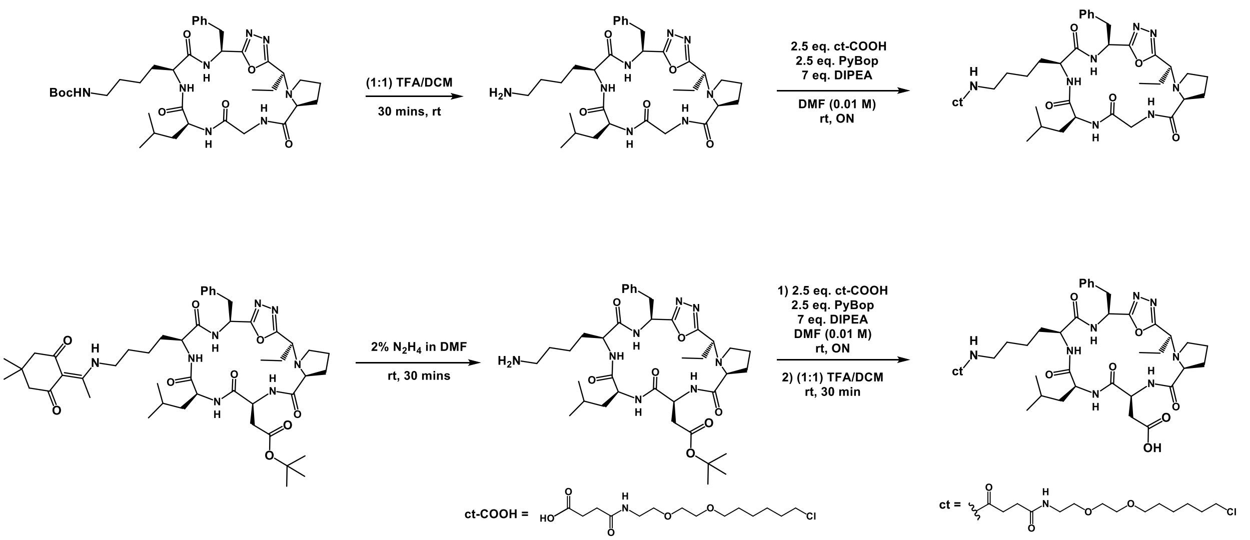

To a 10 mL scintillation vial with a magnetic stir bar, macrocycles with Boc-protected Lys (**3.1 to 3.5**) (25 μmol) was treated with (1:1) TFA/DCM (1 mL) and stirred for 30 minutes at room temperature. The reaction mixture was then concentrated down by gentle stream of N_2. Once dried, mixture was dissolved in DMF (0.01 M) and chloroalkane tag (2.5 eq.), PyBop (2.5 eq.), and DIPEA (7 eq.) was added. The mixture was stirred overnight at room temperature. The reaction mixture was then concentrated down by blowing N_2 and purified using reverse-phase C18 column chromatography. The water/acetonitrile mixture from the product fraction was lyophilized to afford the desired product.

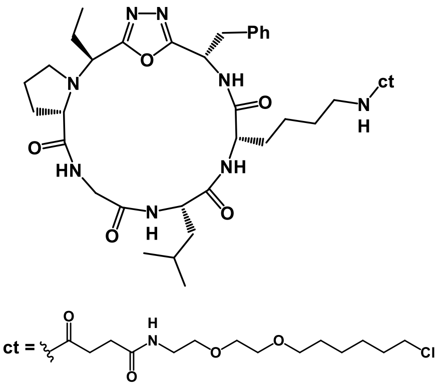

cyclo[PGLK(ct)F] odz/ethyl (1) was prepared using general preparation B to afford the desired product. Off-white solid, 11 mg, 47.8%. Retention time = 8.872 min (Method C). HRMS (ESI) $[M+H]^+$ *m/z* calculated for $C_{46}H_{73}ClN_9O_9$ = 930.5214, *m/z* found = 930.5207.

HPLC trace of Compound 1

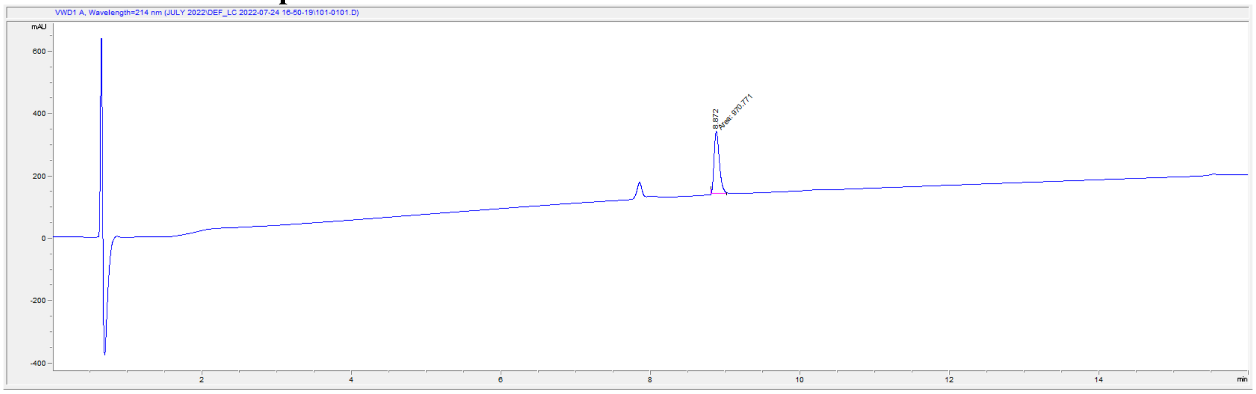

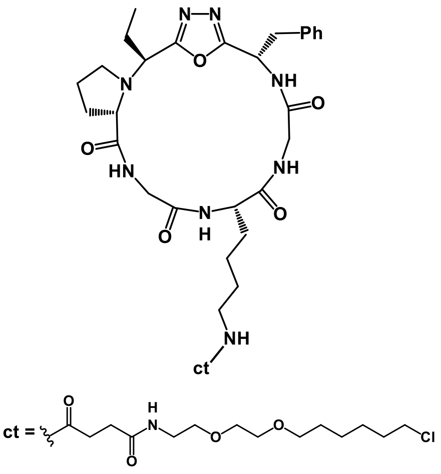

cyclo[PGK(ct)GF] odz/ethyl (2) was prepared using general preparation B to afford the desired product. Off-white solid, 14.2 mg, 65.7%. Retention time = 8.002 min (Method C). HRMS (ESI) $[M+H]^+$ *m/z* calculated for $C_{42}H_{65}ClN_9O_9$ = 874.4588, *m/z* found = 874.4587

HPLC trace of Compound 2

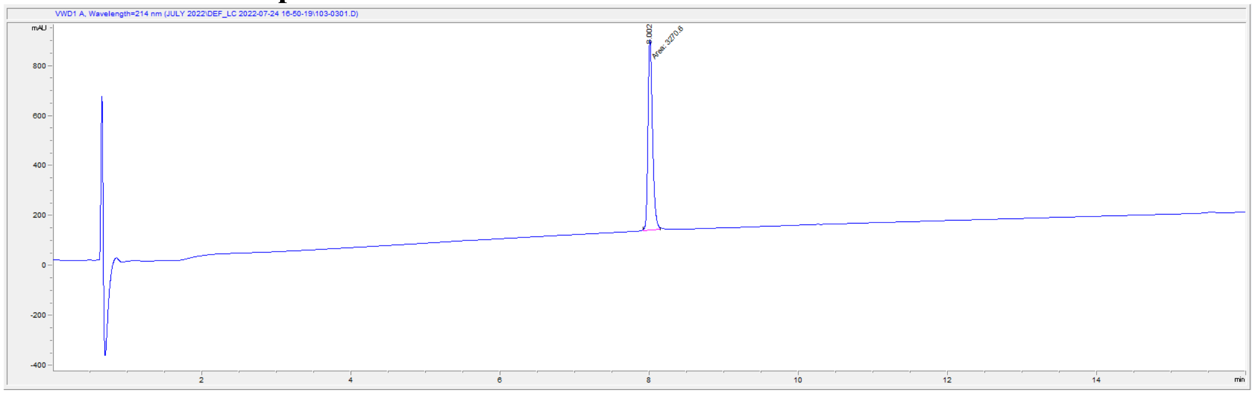

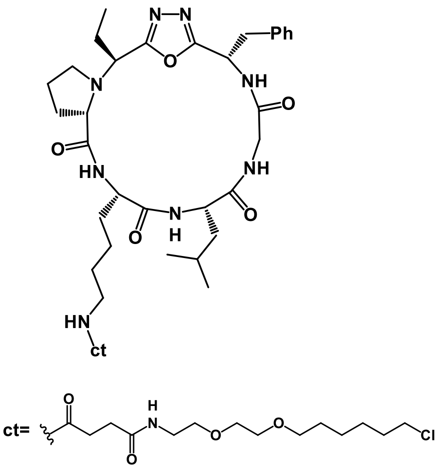

cyclo[PK(ct)LGF] odz/ethyl (3) was prepared using general preparation B to afford the desired product. Off-white solid, 15.9 mg, 69.1%. Retention time = 8.922 min (Method C). HRMS (ESI) $[M+H]^+$ *m/z* calculated for $C_{46}H_{73}ClN_9O_9$ = 930.5214, *m/z* found = 930.5221.

HPLC trace of Compound 3

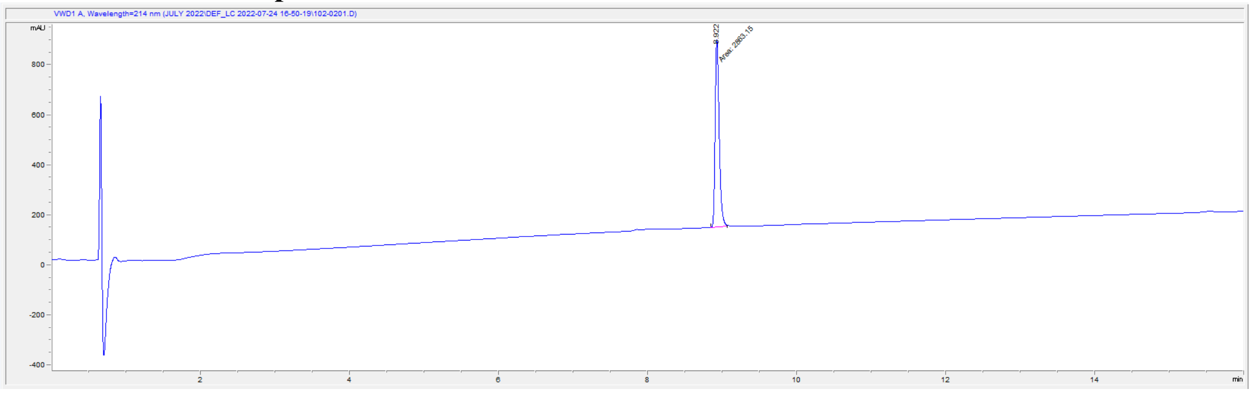

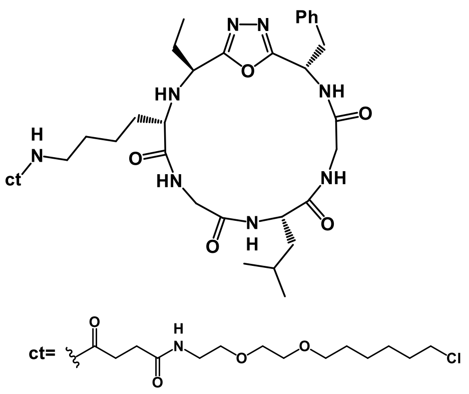

cyclo[K(ct)GLGF] odz/ethyl (4) was prepared using general prep 3.4.5 to afford the desired product. Clear colourless oil, 16.8 mg, 76.4%. Retention time = 8.633 min (Method C). HRMS (ESI) $[M+H]^+$ *m/z* calculated for $C_{43}H_{69}ClN_9O_9$ = 890.4901, *m/z* found = 890.4888.

HPLC trace of Compound 4

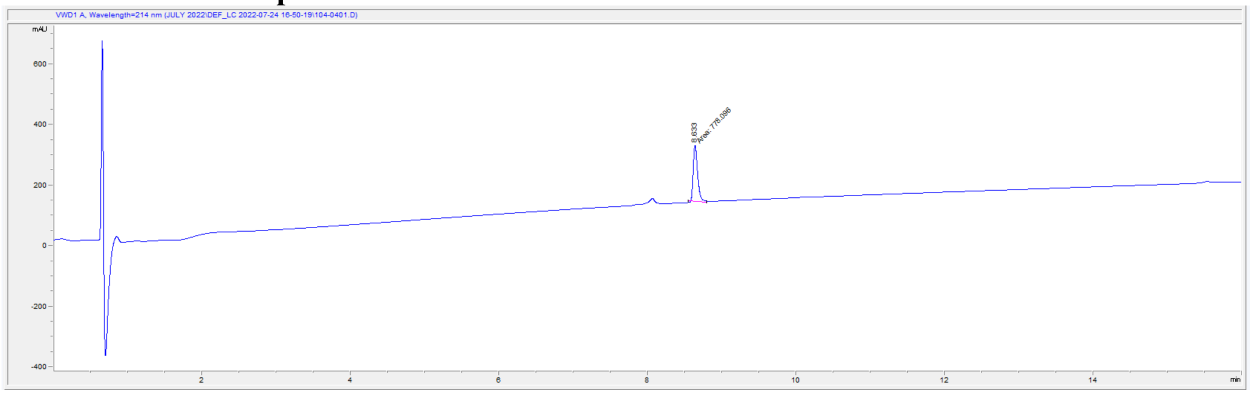

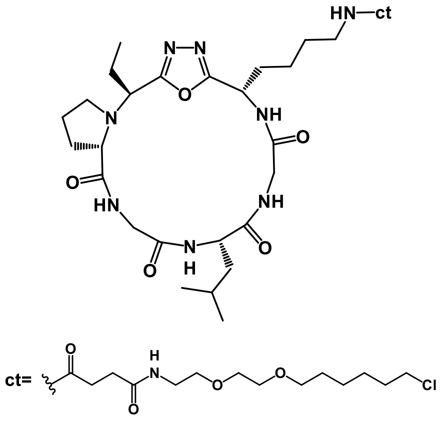

cyclo[PGLGK(ct)] odz/ethyl (5) was prepared using general preparation B to afford the desired product. White solid, 16.8 mg, 80.8%. Retention time = 7.816 min (Method C). HRMS (ESI) $[M+H]^+$ *m/z* calculated for $C_{39}H_{67}ClN_9O_9$ = 840.4745, *m/z* found = 840.4744.

HPLC trace of Compound 5

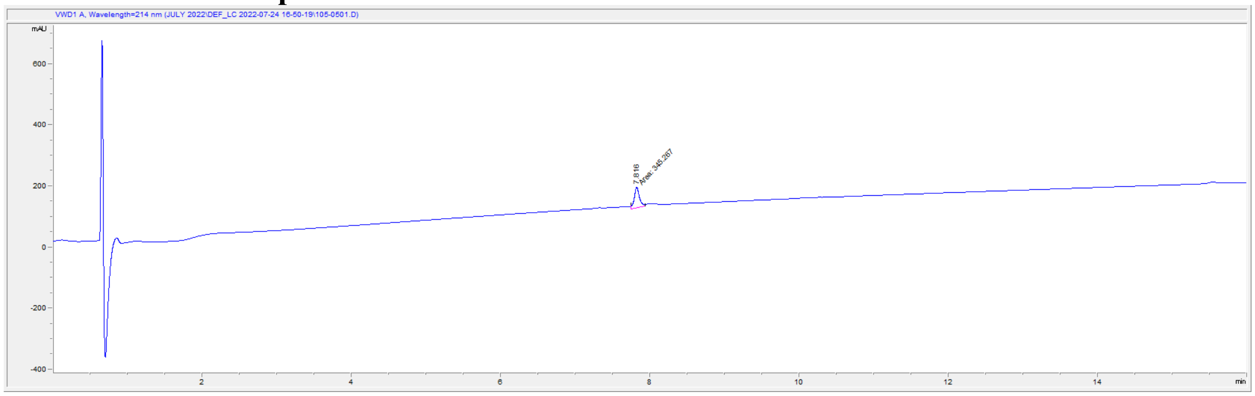

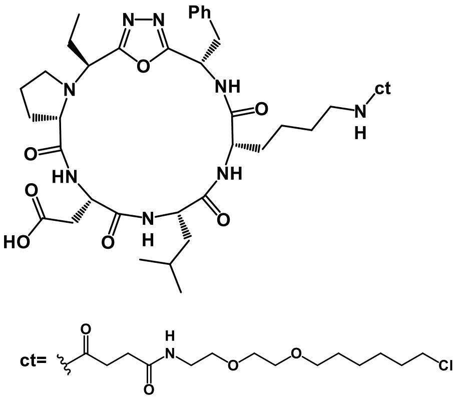

cyclo[PDLK(ct)F] odz/ethyl (6) general preparation B was modified to deprotect Dde protecting group. 2% N_2H_4 in DMF (1 mL) was added to the mixture and stirred for 30 mins. The mixture was dried down by gentle stream of N_2. Coupling of chloroalkane tag is the same as general prep 3.4.5. Further deprotection of the t-Butyl on the Asp residue were done by treating the compound with (1:4) TFA/DCM (0.01 M) and stirred at room temperature for 4 hours. The mixture was dried down by gentle stream of N_2. Reverse-phase C18 column chromatography was performed, and the product fraction was lyophilized to afford the desired product. Off-white solid, 3.5 mg, 39.3%. Retention time = 8.703 min (Method C). HRMS (ESI) $[M+H]^+$ *m/z* calculated for $C_{48}H_{75}ClN_9O_{11}$ = 988.5269, *m/z* found = 988.5258.

HPLC trace of Compound 6

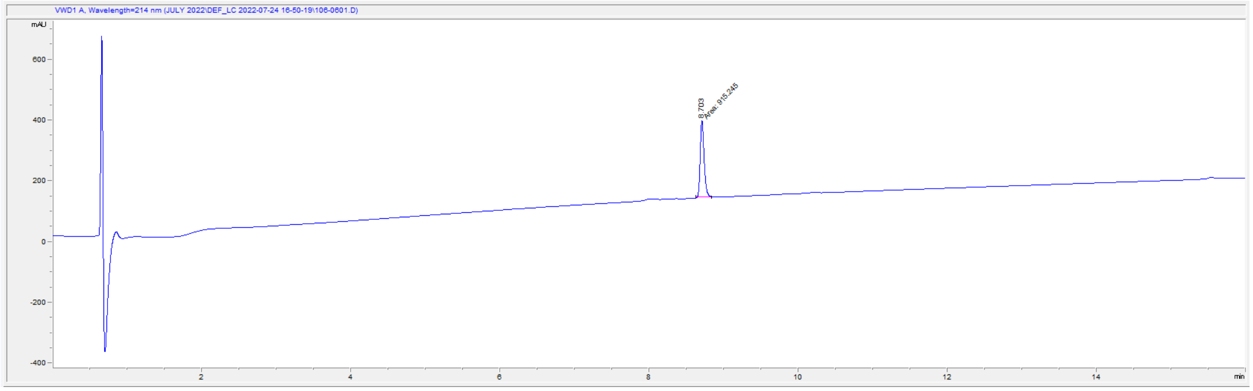

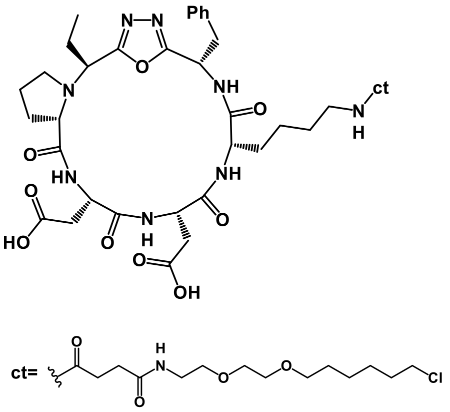

cyclo[PDDK(ct)F] odz/ethyl (7) general preparation B was modified to deprotect Dde protecting group. 2% N_2H_4 in DMF (1 mL) was added to the mixture and stirred for 30 mins. The mixture was dried down by gentle stream of N_2. Coupling of chloroalkane tag is the same as general prep 3.4.5. Further deprotection of the t-Butyl on the Asp residues were done by treating the compound with (1:4) TFA/DCM (0.01 M) and stirred at room temperature for 4 hours. The mixture was dried down by gentle stream of N_2. Reverse-phase C18 column chromatography was performed, and the product fraction was lyophilized to afford the desired product. Off-white solid, 8.6 mg, 35.1%. Retention time = 7.876 min (Method C). HRMS (ESI) $[M+H]^+$ calculated for $C_{46}H_{69}ClN_9O_{13}$ = 990.4698, *m/z* found = 990.4698.

HPLC trace of Compound 7

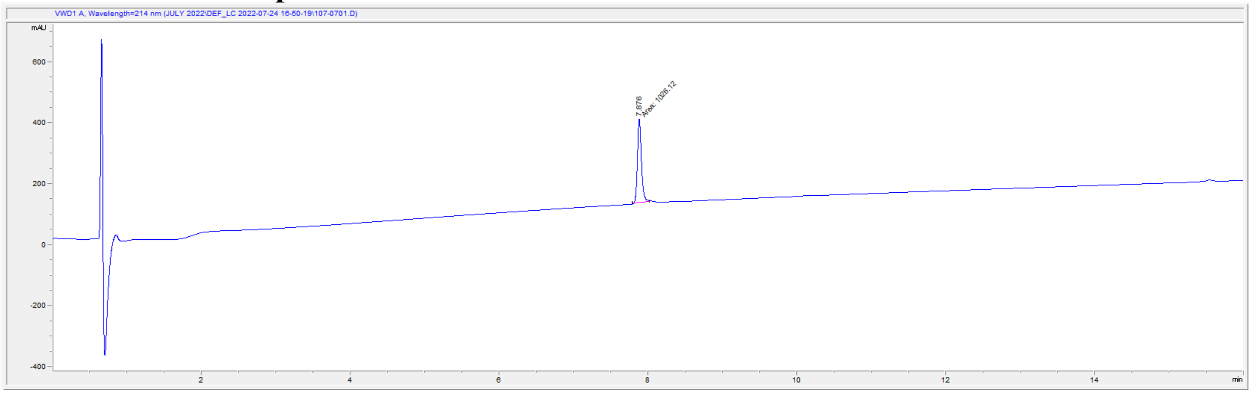

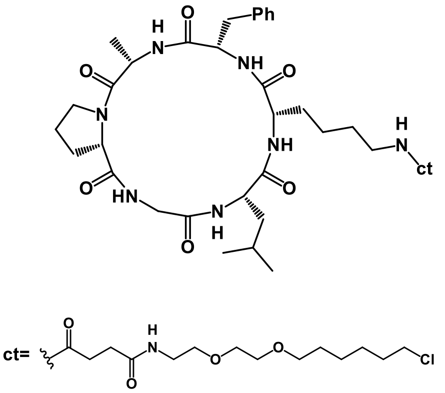

cyclo[K(ct)GLGF] odz/ethyl (8) was prepared using general prep 3.4.5 to afford the desired product. Clear colourless oil, 16.8 mg, 76.4%. Retention time = 7.945 min (Method C). HRMS (ESI) $[M+H]^+$ *m/z* calculated for $C_{45}H_{72}ClN_8O_{10}$ = 919.5054, *m/z* found = 919.5051.

HPLC trace of Compound 8

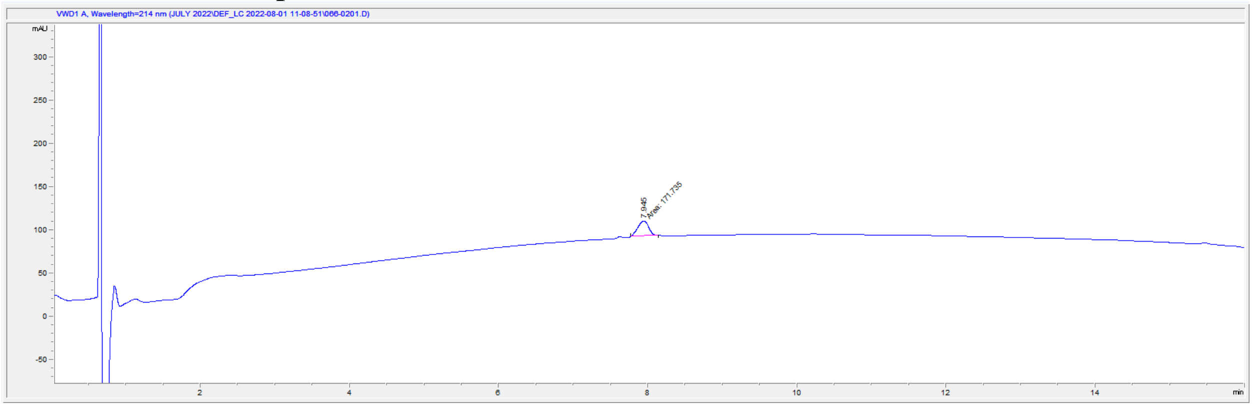

3 Chloroalkane Penetration Assay (CAPA)

CAPA was performed as previously described.[5,6] Briefly, HeLa cells stably expressing the HaloTag-GFP-Mito construct[7] were cultured in DMEM + 10% FBS + 1% Pen/Strep + 1 µg/mL puromycin. Cells were seeded in a 96-well plate the day before the experiment at a density of 3 x 10^4 cells per well. The day of the experiment, the media was aspirated, and cells were treated with serum-free media and 8-12 serial dilutions of ct-molecules, ct-R9W or ct-W control. The plates were incubated for 30 mins or 4 h at 37 °C with 5% CO_2. Media was aspirated and cells were washed with serum-free media. Cells were treated with 5 µM ct-TAMRA for 15 min, except for the no-ct-TAMRA control wells, which were incubated with serum-free media. Excess dye was washed away, and cells were trypsinized and resuspended in 180 µL phosphate-buffered saline. The cells were analyzed by flow cytometry (Guava easyCyte HT benchtop flow cytometer), gating for live, HaloTag expressing cells and measuring 5,000 cells per well. Fluorescence was normalized to a no-molecule control (no ct-molecule during incubation, chased with ct-TAMRA, to indicate value for 100% fluorescence) and a no-dye control (no ct-molecule during incubation, chased with media without ct-TAMRA, to indicate value for 0% fluorescence).

4 Lysate stability assay method

Lysate stability assays were performed essentially as described.[8] For lysate stability assays, HeLa cells were trypsinized, washed in PBS, pelleted, and treated with lysis buffer (50 mM Tris, 250 mM NaCl, 0.5 % IGEPAL CA-630 detergent, pH 8.0) on ice for 15 min. Then, lysates were centrifuged for 10 min at 4 °C and 21,000 *g*, and the clarified lysate was collected. Peptides were added to the lysate to a concentration of 150 µM and were incubated at 37 °C. Aliquots of 40 µL

were taken at each time point and quenched in 160 µL of ice-cold methanol. Samples were spun down for 10 min at 21,000 *g* prior to analysis by reverse phase analytical HPLC on a C_{18} column. Chromatogram peaks were integrated to measure amount of peptide remaining at each time point. Peak volumes for each timepoint were normalized to the zero-hour timepoint to determine percentage of peptide remaining. Data presented are the average of three biological replicates performed with different lysates on different days.

5 Parallel Artificial Membrane Permeability Assay (PAMPA)

PAMPA analysis was conducted following a procedure reported by Lokey and coworkers.[9] Each measurement was conducted at room temperature in an insulated polystyrene box to maintain a constant internal temperature. Measurements were taken in triplicate, and the reported permeability values are the average value over the three runs. Carbamazepine was included in the stock solution of each cyclic peptide to verify the integrity of the membrane as an internal standard. Measurements which did not give consistent permeability values (-Log Papp ±0.1) were repeated in triplicate. In our hands, we consistently achieved permeability values of -Log Papp = ~5.7–5.8 for carbamazepine (Literature value using this method: -Log Papp = 5.1) [6] which indicates that our reported values are at worst case underestimated.

Preparation of stock solution for donor well: Stock solutions were prepared by dissolving the cyclic peptide (0.5 mM) and carbamazepine (0.5 mM) (as an internal standard) in DMSO at a gross concentration of 1 mM. 10 µL of this stock solution was added to 990µL PBS buffer (pH 7.4) to give a 1 mL solution (10 µM, 1% DMSO v/v) for addition to the donor plate wells.

Preparation of acceptor plate: A stock solution of 5% DMSO in PBS buffer was prepared, and 300µL of this solution was added to each of the acceptor wells (96-well Teflon acceptor plate (Millipore MSSACCEPTOR)).

Preparation of donor plate: 100 mg of lethicin (90%, soybean) was suspended in n-dodecane (10 mL) and sonicated to give a homogenous, clear solution. 5 µL of this solution was carefully applied to the membrane in the 96-well donor plate (with 0.45 µm hydrophobic Immobilon-P membrane supports (Millipore MAIPNTR10)) (with care, without touching the membrane). Shortly after (~5-15 min), 150 µL of the 10 µM stock solution of cyclic peptide and carbamazepine (prepared as above) were added to the donor well. The donor plate was placed on top of the acceptor wells and

visually inspected to ensure no air bubbles had formed around the membrane in contact with the acceptor solution. The lid was placed on top of the plates and the system was moved to an insulated polystyrene box with a damp paper towel inside to prevent evaporation. The box was sealed and left overnight (16 hours) with the exact time of acceptor and donor plate separation recorded for analysis.

Analysis of donor/acceptor wells: 100μL of the solution in each of the donor and acceptor wells were taken separately and added to a 1-dram vial and sealed immediately to prevent evaporation. The solution was sealed until ready for injection to LC-MS. The solutions were analysed by LC-MS (15 μL injection) using the LC-MS method for PAMPA analysis (see Page 3). Using EIC mode, the peak area was measured for analyte in the donor and acceptor wells, and the Papp value was calculated using the method reported by Lokey and coworkers.[9]

6 Variable temperature NMR

^{1}H-NMR spectra were acquired from 298 K to 318 K in 5 K increments in DMSO-*d6*. Temperature shift coefficients below 4 ppb/K were selected to represent a strong intramolecular hydrogen bonding interaction and temperature shift coefficients above 4 ppb/K were attributed to solvent exposed protons.[10]

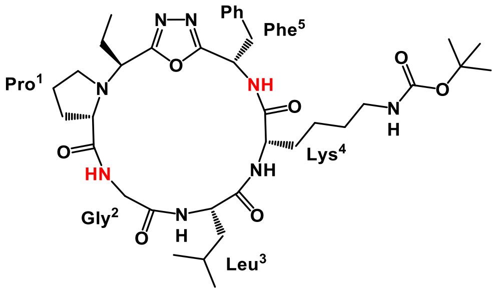

Compound 1a

Temp (K)	Gly^{2}-NH	Leu^{3}-NH	Lys^{4}-NH	Phe^{5}-NH	Boc-NH
298	7.84	8.43	8.27	7.93	6.75
303	7.83	8.41	8.25	7.92	6.72
308	7.83	8.39	8.23	7.91	6.68
313	7.82	8.37	8.21	7.90	6.65
318	7.81	8.34	8.18	7.89	6.62
T_{coeff}	-1.78	-4.36	-4.41	-1.66	-6.39

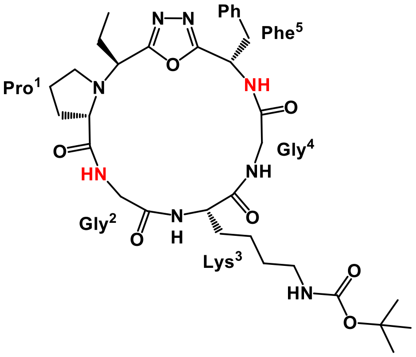

Compound 2a

Temp (K)	Gly^{2}-NH	Lys^{3}-NH	Gly^{4}-NH	Phe^{5}-NH	Boc-NH
298	7.87	8.62	8.67	8.01	6.76
303	7.87	8.60	8.65	8.00	6.73
308	7.85	8.58	8.62	7.99	6.70
313	7.84	8.56	8.60	7.99	6.66
318	7.84	8.53	8.57	7.98	6.63
T_{coeff}	-1.92	-4.50	-4.91	-1.54	-6.19

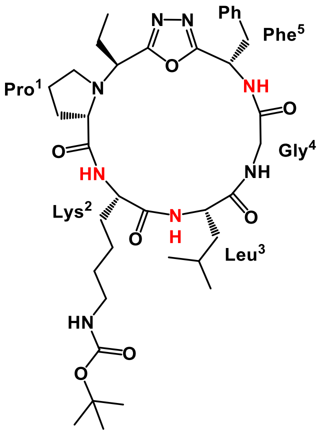

Compound 3a

Temp (K)	Lys^{2}-NH	Leu^{3}-NH	Gly^{4}-NH	Phe^{5}-NH	Boc-NH
298	8.21	8.71	8.74	8.27	6.71
303	8.20	8.69	8.72	8.27	6.68
308	8.19	8.67	8.67	8.26	6.64
313	8.17	8.65	8.65	8.26	6.61
318	8.16	8.64	8.64	8.25	6.58
T_{coeff}	-2.56	-3.8	-5.45	-1.22	-6.22

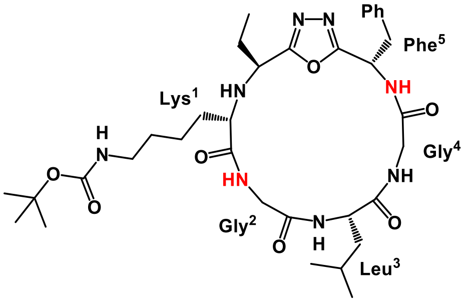

Compound 4a

Temp (K)	Gly^2-NH	Leu^3-NH	Gly^4-NH	Phe^5-NH	Boc-NH
298	7.93	8.41	8.49	8.16	6.74
303	7.92	8.39	8.47	8.15	6.71
308	7.90	8.36	8.44	8.13	6.68
313	7.88	8.34	8.41	8.12	6.65
318	7.87	8.31	8.39	8.10	6.62
T_{coeff}	-3.36	-4.95	-5.15	-3.14	-6.31

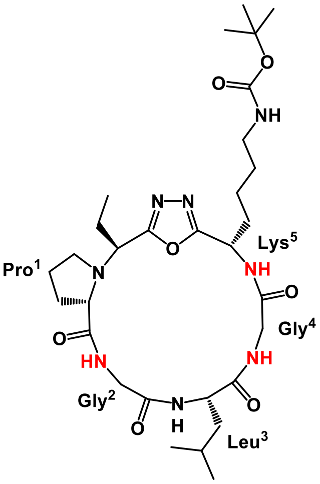

Compound 5a

Temp (K)	Gly^2-NH	Leu^3-NH	Gly^4-NH	Lys^5-NH	Boc-NH
298	8.07	8.71	8.59	7.92	6.72
303	8.05	8.69	8.57	7.91	6.69
308	8.04	8.67	8.55	7.90	6.66
313	8.03	8.65	8.53	7.89	6.63
318	8.02	8.62	8.51	7.88	6.60
T_{coeff}	-2.15	-4.65	-3.92	-2.02	-6.12

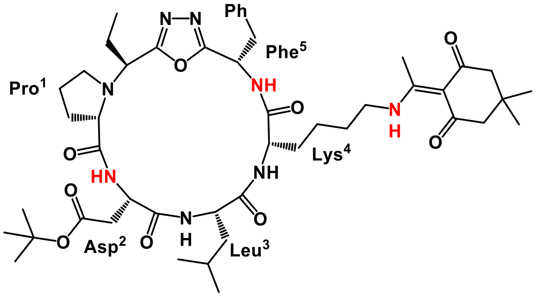

Compound 6a

Temp (K)	Asp^2-NH	Leu^3-NH	Lys^4-NH	Phe^5-NH	Dde-NH
298	8.25	8.74	8.17	7.93	13.25
303	8.24	8.72	8.15	7.93	13.25
308	8.23	8.70	8.12	7.93	13.25
313	8.22	8.67	8.09	7.92	13.25
318	8.21	8.65	8.06	7.92	13.25
T_{coeff}	-2.09	-4.45	-5.44	-0.69	-0.14

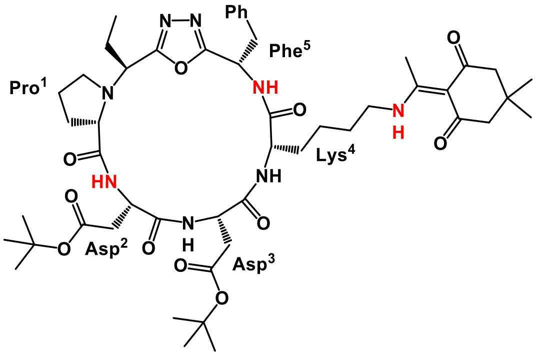

Compound 7a

Temp (K)	Asp^2-NH	Asp^3-NH	Lys^4-NH	Phe^5-NH	Boc-NH
298	8.17	9.17	8.19	8.00	13.26
303	8.16	9.14	8.16	7.98	13.26
308	8.15	9.11	8.14	7.97	13.26
313	8.14	9.08	8.12	7.96	13.26
318	8.14	9.05	8.10	7.95	13.25
T_{coeff}	-1.85	-6.18	-4.59	-2.20	-0.65

7 Structure Determination

ROE-based restraint: The NMR structures were determined by NMR derived distance information. ROESY spectra were integrated by using MestreNova (v. 10.0.2, Mestrelab Research S.L.) software. Integrated volumes of ROE cross peaks were converted to proton interatomic distances using an inverse sixth power relationship. A reference integral was calculated as the average integral between sets of geminal protons which was then set to the calculated geminal interproton distance of 1.78 Å. The calculated distances were adjusted upwards and downwards by 10% to give upper and lower bounds to account for uncertainty in interproton distances. ^{3}J coupling constants were recorded from the 1H spectrum. NH-CαH ^{3}J coupling constants of < 6 Hz were assigned phi dihedral values of -60° +/- 25°. NHCαH ^{3}J coupling constants of > 8 Hz were assigned phi values of -120° +/- 25°. Crude structures of macrocycles were generated by a restrained Monte Carlo low mode molecular mechanics conformational search with an implicit solvent model (DMSO) in Macromodel (Schrodinger LLC, v11.0). The structures were then checked for violations of the experimental distances and dihedral restraints. The lowest energy structure that satisfied these tests were passed for molecular dynamics study. **Molecular dynamics:** Solvent explicit molecular dynamics simulations were carried out with the Desmond Molecular Dynamics software module (D.E. Shaw, v4.4) running inside Maestro (Schodinger LLC, v2015-2). The OPLS3e force field was used for parameterization of the peptidomimetic macrocycle. The macrocycle representative structure was placed in an orthorhombic box solvent box (DMSO) with a minimum distance of 12 Å between solute atoms and the box boundary. The solvated box was minimized then brought to 300 K from 10 K using a restrained dynamics regime. Coulombic interactions were grouped into near- and far interactions with a near-interaction cutoff of 9 Å. Bonds were constrained with the SHAKE algorithm and an integration time step of 2 fs was used. The final MD production run was 10 ns in length with energy value recording every 1.2 ps and trajectory recording every 4.8 ps. The trajectory run was clustered using the Trajectory Clustering script within Maestro with a 0.4 Å RMSD cutoff for variation between backbone heavy atoms and a sampling frequency of 10%. The most populated cluster was taken as the "preferred" structure i.e. that conformation which the molecule spent most time in the dynamics run. The average interproton distances were measured, compared to the experimental NMR derived distances and the violations were tabulated below.

7.1 Conformation and distance restraint

Compound 1a

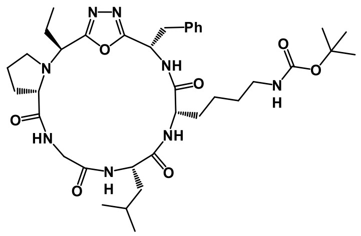

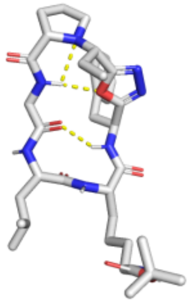

Residue 1	Atom 1	Residue 2	Atom 2	Calculated NOE (Å)	NOE Upper Bound (Å)	NOE Lower Bound (Å)	MD Average Distance (Å)	Violation (Å)
Lys4	NH	Phe5	NH	2.93	3.23	2.64	2.98	0.00
Gly2	Hα′	Leu3	NH	3.04	3.35	2.74	2.85	0.00
Gly2	Hα″	Leu3	NH	2.72	2.98	2.44	2.75	0.00
Pro1	Hα	Gly2	NH	2.77	3.05	2.50	3.45	0.40
Ethyl	Hα	Pro1	Hα	2.71	2.98	2.44	3.00	0.02
Ethyl	Hα	Pro1	Hδ’	2.21	2.44	1.99	2.22	0.00

Compound 2a

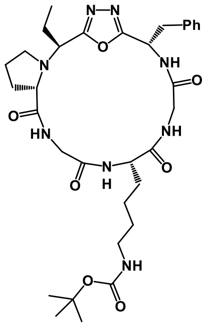

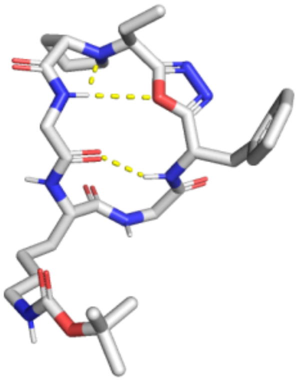

Residue 1	Atom 1	Residue 2	Atom 2	Calculated NOE (Å)	NOE Upper Bound (Å)	NOE Lower Bound (Å)	MD Average Distance (Å)	Violation (Å)
Phe5	NH	Gly4	NH	2.71	2.98	2.44	2.42	0.02
Gly4	NH	Lys3	Hα	2.19	2.41	1.97	2.95	0.02
Gly2	Hα′	Lys3	NH	3.19	3.51	2.87	3.33	0.00
Gly2	Hα″	Lys3	NH	2.77	3.05	2.50	2.23	0.27
Phe5	NH	Gly4	Hα′	3.42	3.76	3.07	3.38	0.00
Gly2	NH	Ethyl	Hα	3.83	4.22	3.45	3.76	0.00
Gly2	NH	Pro1	Hα	3.19	3.51	2.87	3.30	0.00
Ethyl	Hα	Pro1	Hα	2.84	3.13	2.56	2.95	0.00
Ethyl	Hα	Pro1	Hδ’	2.33	2.56	2.09	2.29	0.00
Ethyl	Hα	Pro1	Hδ”	3.83	4.22	3.45	3.24	0.21

Compound 3a

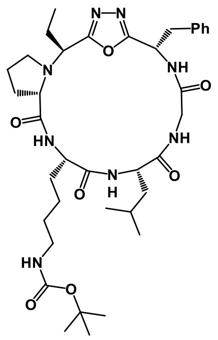

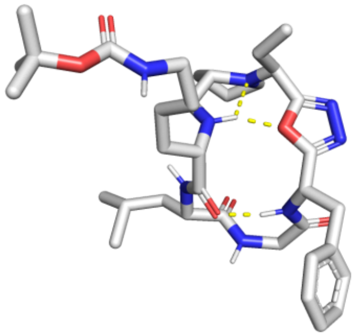

Residue 1	Atom 1	Residue 2	Atom 2	Calculated NOE (Å)	NOE Upper Bound (Å)	NOE Lower Bound (Å)	MD Average Distance (Å)	Violation (Å)
Gly4	NH	Phe5	NH	2.61	2.87	2.35	2.56	0.00
Gly4	NH	Leu3	Hα	2.04	2.25	1.84	2.24	0.00
Lys2	Hα	Leu3	NH	2.31	2.54	2.08	2.92	0.38
Phe5	NH	Gly4	Hα′	3.42	3.76	3.07	3.20	0.00
Phe5	NH	Gly4	Hα″	3.83	4.22	3.45	3.46	0.00
Phe5	NH	Leu3	Hα	3.83	4.22	3.45	3.60	0.00
Lys2	NH	Ethyl	Hα	3.83	4.22	3.45	3.59	0.00
Lys2	NH	Pro1	Hα	3.42	3.76	3.07	3.12	0.00

Compound 4a

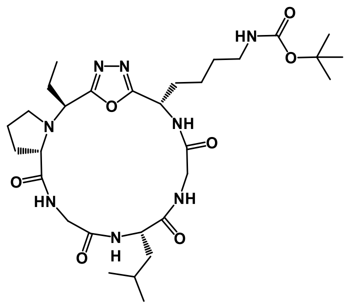

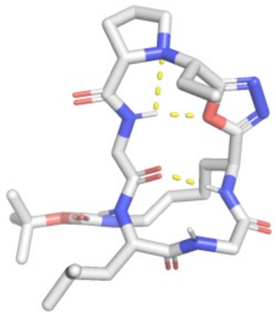

Residue 1	Atom 1	Residue 2	Atom 2	Calculated NOE (Å)	NOE Upper Bound (Å)	NOE Lower Bound (Å)	MD Average Distance (Å)	Violation (Å)
Gly4	NH	Leu3	Hα	2.31	2.54	2.08	2.03	0.05
Gly4	NH	Lys5	NH	2.53	2.79	2.28	2.50	0.00
Leu3	NH	Gly2	Hα′	2.50	2.75	2.25	2.43	0.00
Leu3	NH	Gly2	Hα″	2.84	3.13	2.56	2.65	0.00
Lys5	NH	Gly4	Hα′	3.19	3.51	2.87	3.02	0.00
Lys5	NH	Gly4	Hα″	3.42	3.76	3.07	3.54	0.00
Lys5	NH	Leu3	Hα	3.83	4.22	3.45	3.82	0.00

Compound 5a

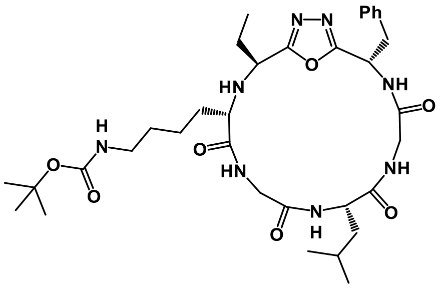

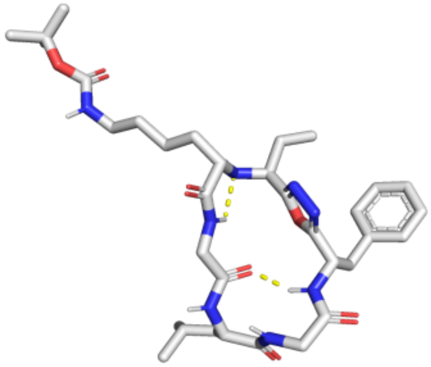

Residue 1	Atom 1	Residue 2	Atom 2	Calculated NOE (Å)	NOE Upper Bound (Å)	NOE Lower Bound (Å)	MD Average Distance (Å)	Violation (Å)
Leu3	NH	Gly2	Hα’	2.50	2.75	2.25	2.29	0.00
Leu3	NH	Gly2	Hα”	2.93	3.23	2.64	2.66	0.00
Gly4	NH	Leu3	Hα	2.33	2.56	2.09	2.58	0.02
Gly4	NH	Phe5	NH	2.77	3.05	2.50	2.49	0.01
Phe5	NH	Gly4	Hα’	3.19	3.51	2.87	3.27	0.00
Phe5	NH	Gly4	Hα”	3.42	3.76	3.07	3.38	0.00
Gly2	NH	Ethyl	Hα	3.19	3.51	2.87	3.20	0.00
Gly2	NH	Lys1	Hα	3.19	3.51	2.87	3.21	0.00
Ethyl	Hα	Lys1	Hα	2.57	2.83	2.31	3.81	0.98

Compound 6a

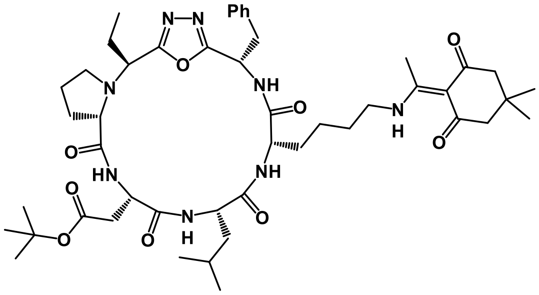

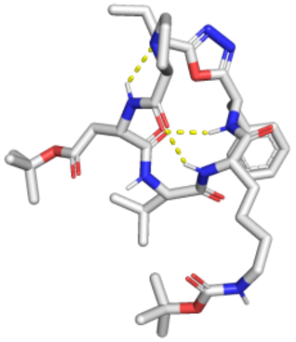

Residue 1	Atom 1	Residue 2	Atom 2	Calculated NOE (Å)	NOE Upper Bound (Å)	NOE Lower Bound (Å)	MD Average Distance (Å)	Violation (Å)
Leu3	NH	Asp2	Hα	2.42	2.66	2.17	2.46	0.00
Leu3	NH	Leu3	Hα	2.53	2.79	2.28	2.12	0.16
Asp2	NH	Asp2	Hα	2.84	3.13	2.56	2.87	0.00
Asp2	NH	Pro1	Hα	3.83	4.22	3.45	3.24	0.21
Lys4	NH	Leu3	Hα	2.47	2.72	2.22	3.20	0.48
Phe5	NH	Lys4	Hα	3.19	3.51	2.87	3.13	0.00
Ethyl	Hα	Pro1	Hδ’	2.20	2.42	1.98	3.78	1.36
Ethyl	Hα	Pro1	Hδ”	3.42	3.76	3.07	3.79	0.03

Compound 7a

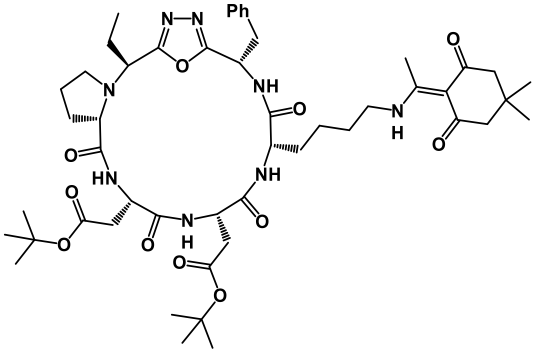

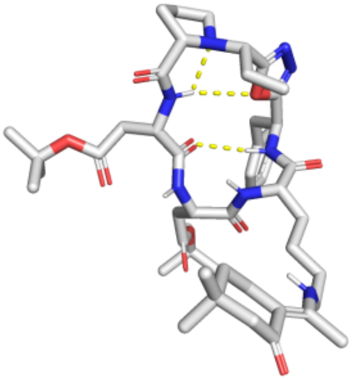

Residue 1	Atom 1	Residue 2	Atom 2	Calculated NOE (Å)	NOE Upper Bound (Å)	NOE Lower Bound (Å)	MD Average Distance (Å)	Violation (Å)
Asp3	NH	Asp2	Hα	2.39	2.63	2.15	2.24	0.00
Lys4	NH	Asp3	Hα	3.04	3.35	2.74	2.92	0.00
Asp2	NH	Pro1	Hα	3.83	4.22	3.45	3.38	0.07
Phe5	NH	Lys4	Hα	3.42	3.76	3.07	3.13	0.00
Asp2	Hα	Asp3	Hα	3.42	3.76	3.07	4.29	0.43
Ethyl	Hα	Pro1	Hδ’	2.20	2.42	1.98	2.36	0.00
Ethyl	Hα	Pro1	Hδ”	3.42	3.76	3.07	3.03	0.00

8 NMR spectra

cyclo[PGLK(Boc)F] odz/ethyl (1a)

^{1}H NMR (500 MHz, DMSO-d_6) at 25°C

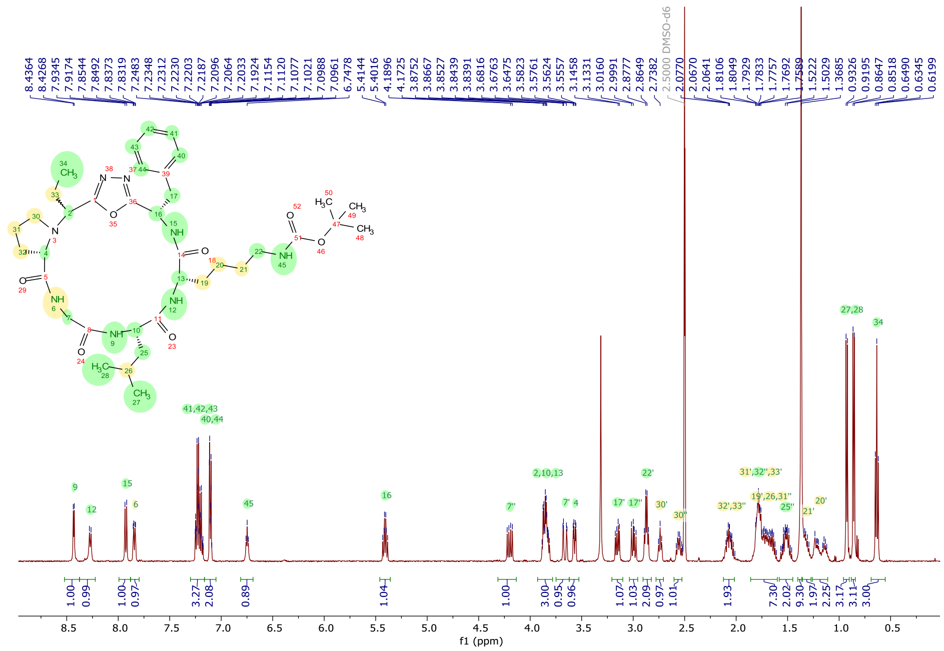

^{13}C NMR (126 MHz, DMSO-d_6) at 25°C

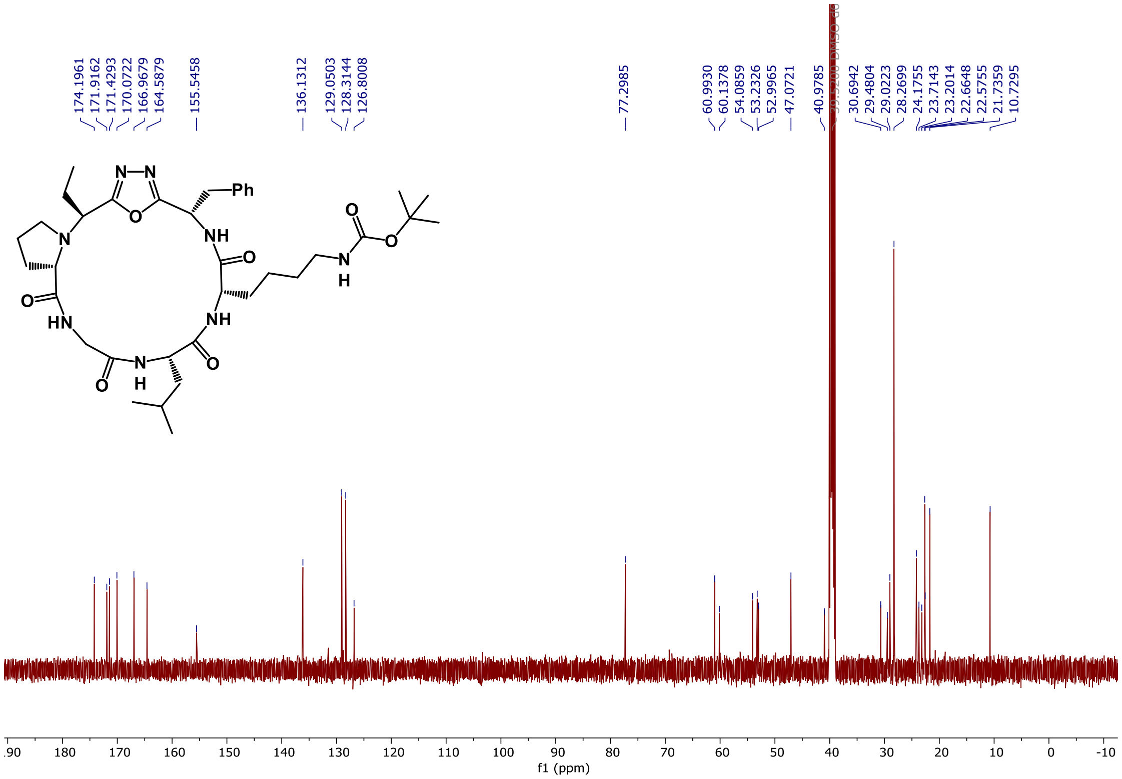

COSY at 25°C

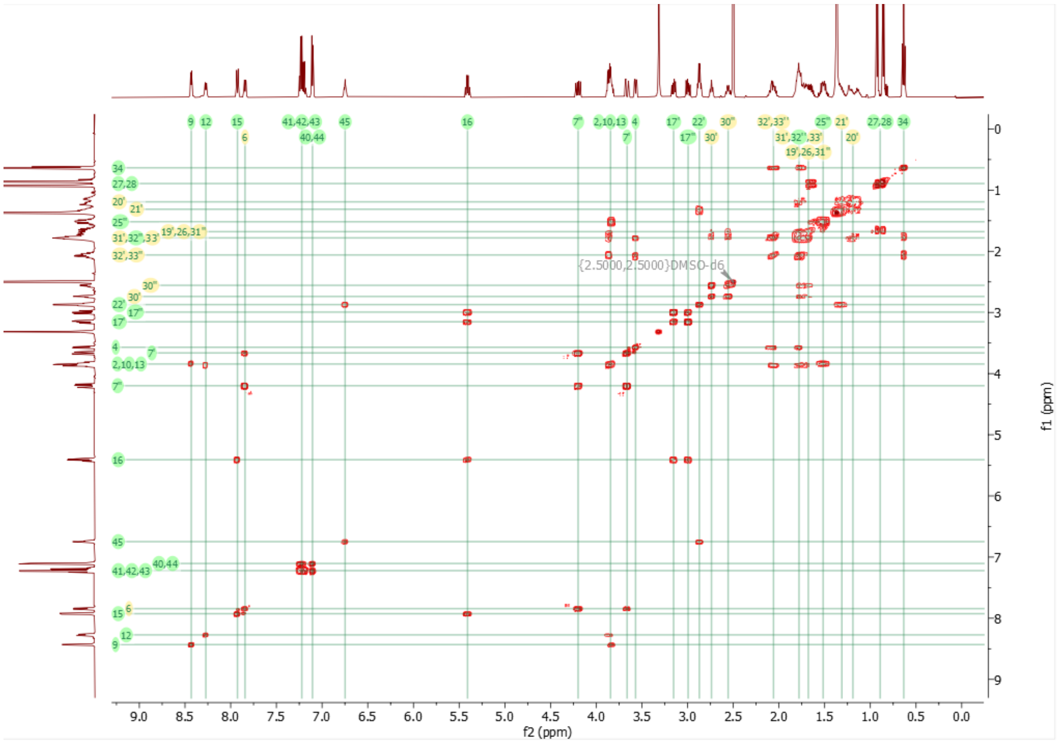

TOCSY at 25°C

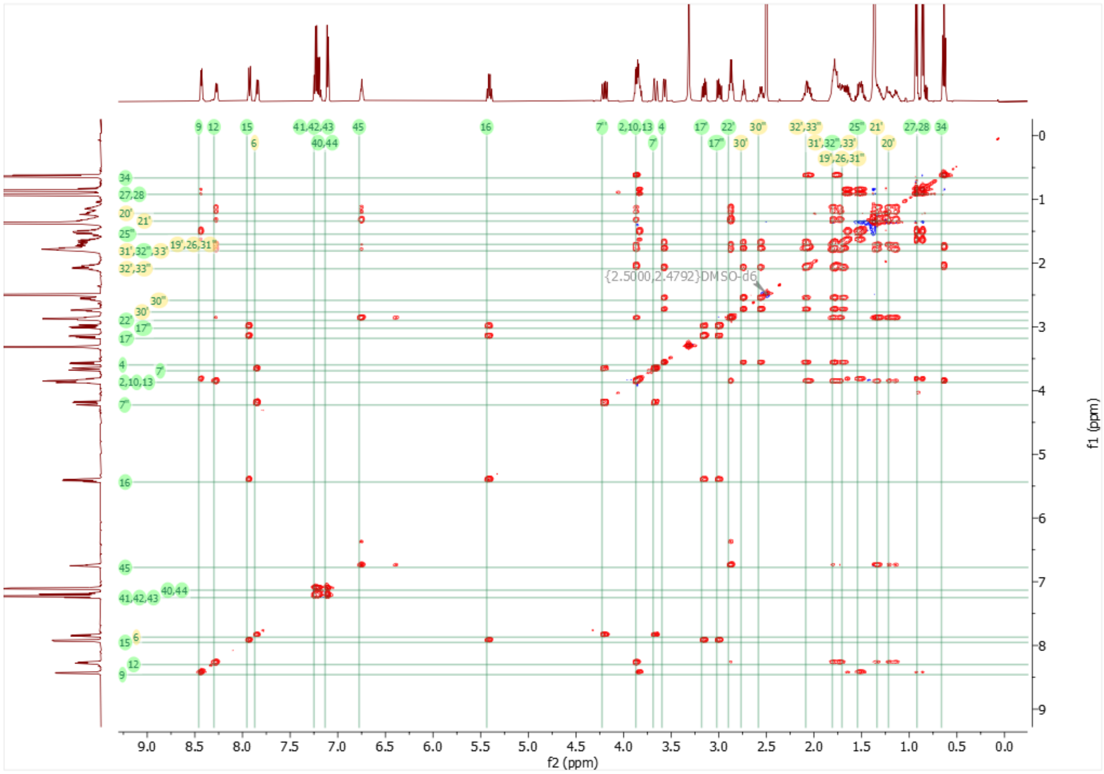

ROESY at 25°C

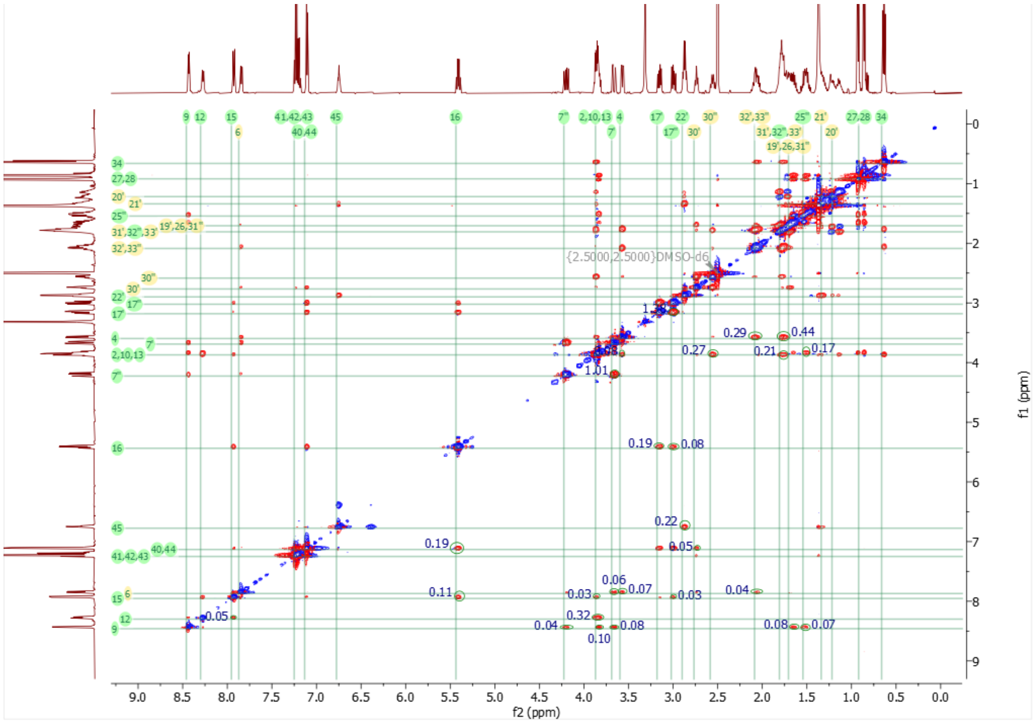

HSQC at 25°C

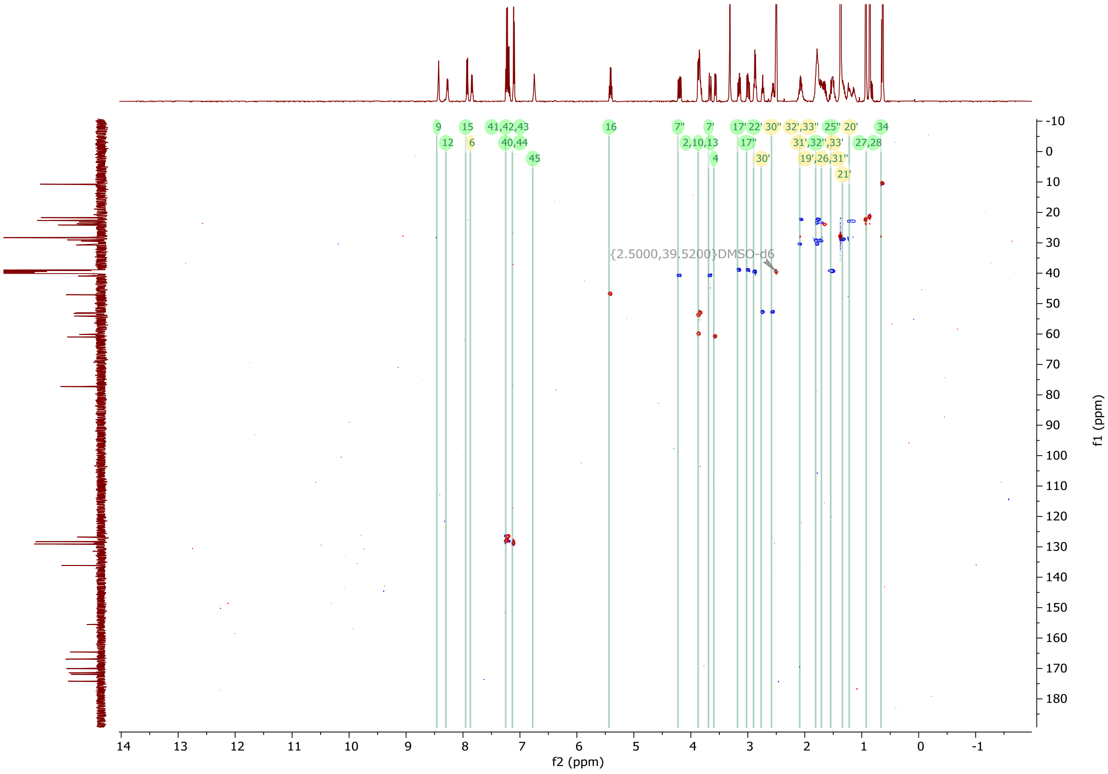

HMBC at 25°C

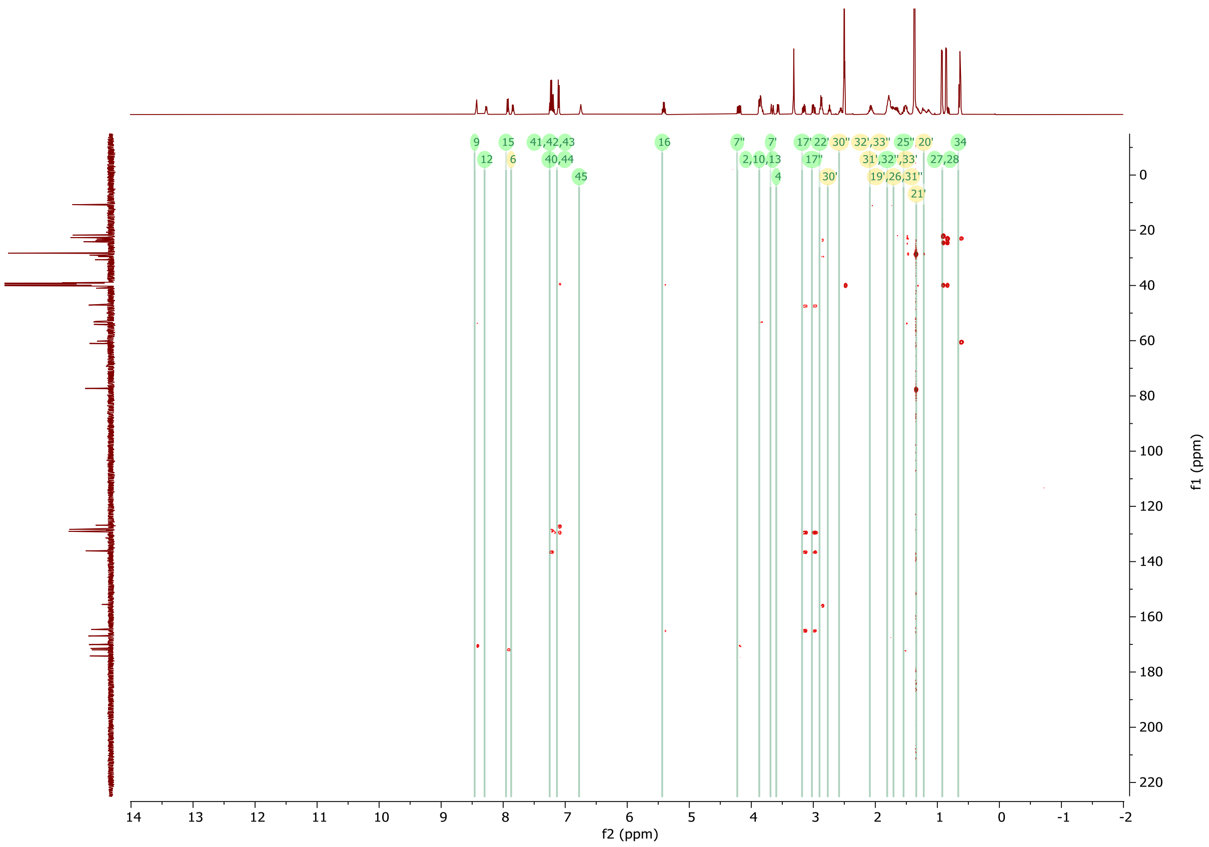

9
12
15
6
41,42,43
40,44
45
16
7''
2,10,13
7'
4
17'
17''
22'
30'
30''
32',33''
31',32'',33'
19',26,31''
25''
20'
21'
27,28
34
f2 (ppm)
f1 (ppm)

cyclo[PGK(Boc)GF] odz/ethyl (2a)

^{1}H NMR (500 MHz, DMSO-d_6) at 25°C

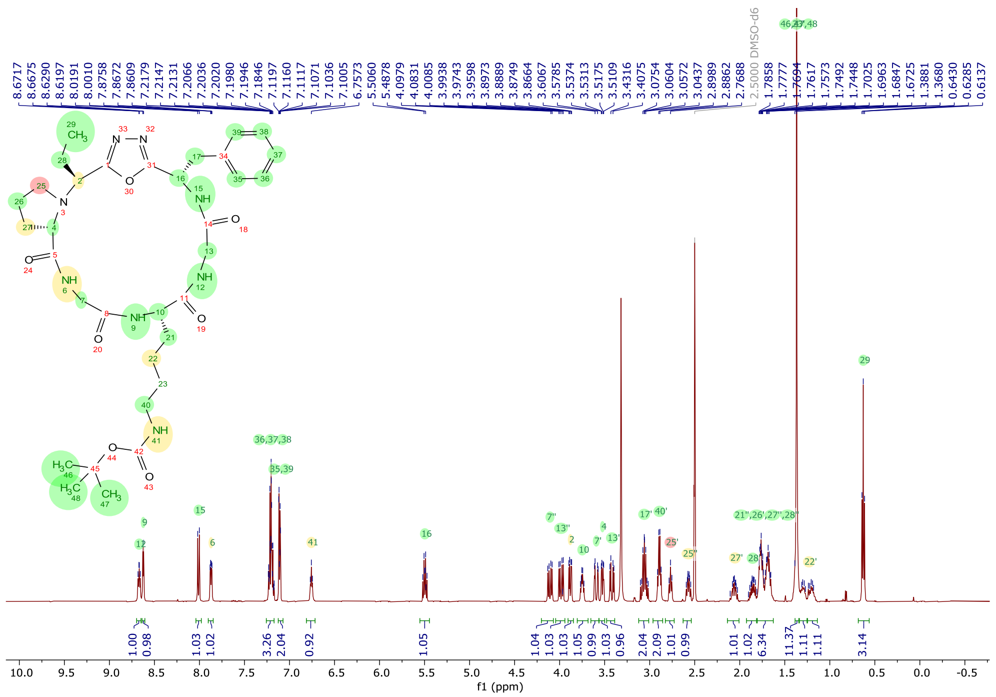

^{13}C NMR (126 MHz, DMSO-d_6) at 25°C

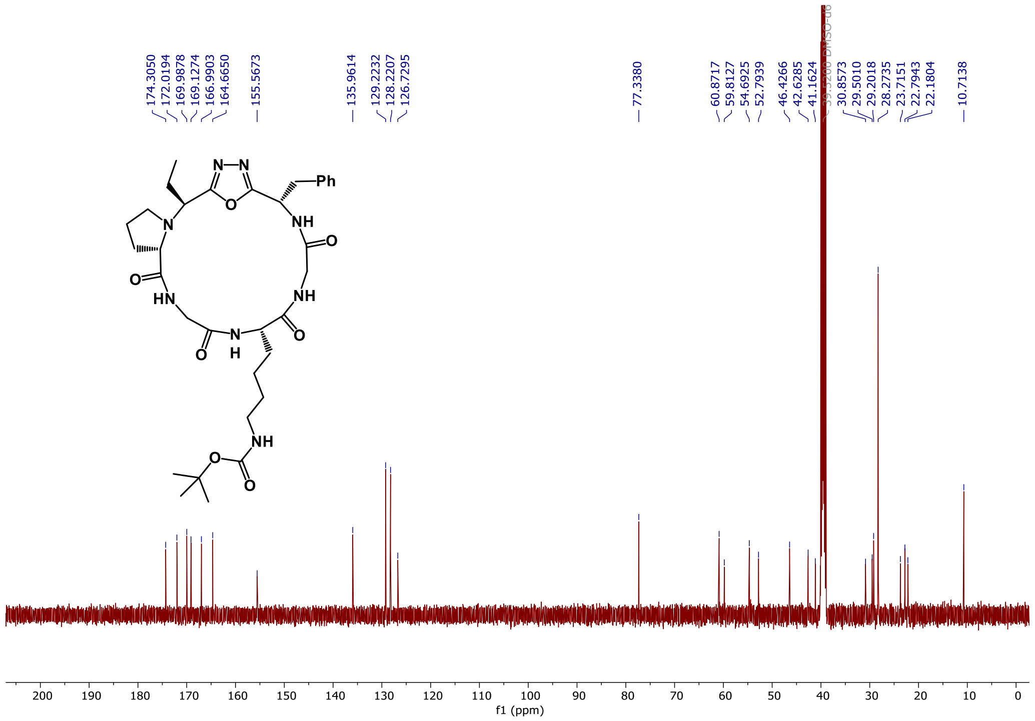

COSY at 25°C

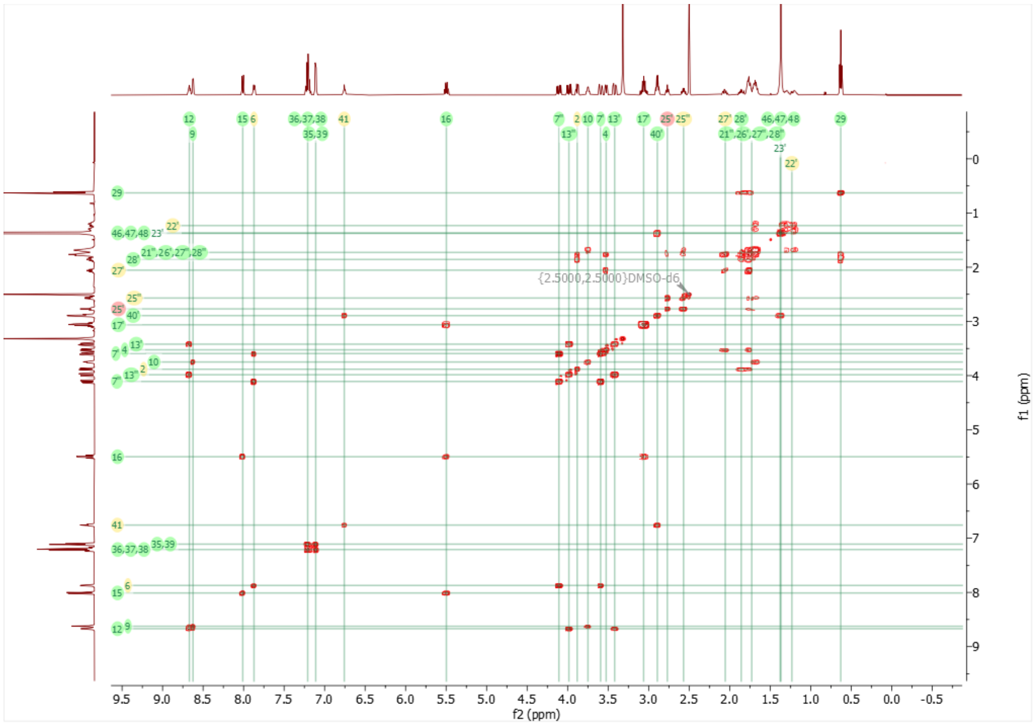

TOCSY at 25°C

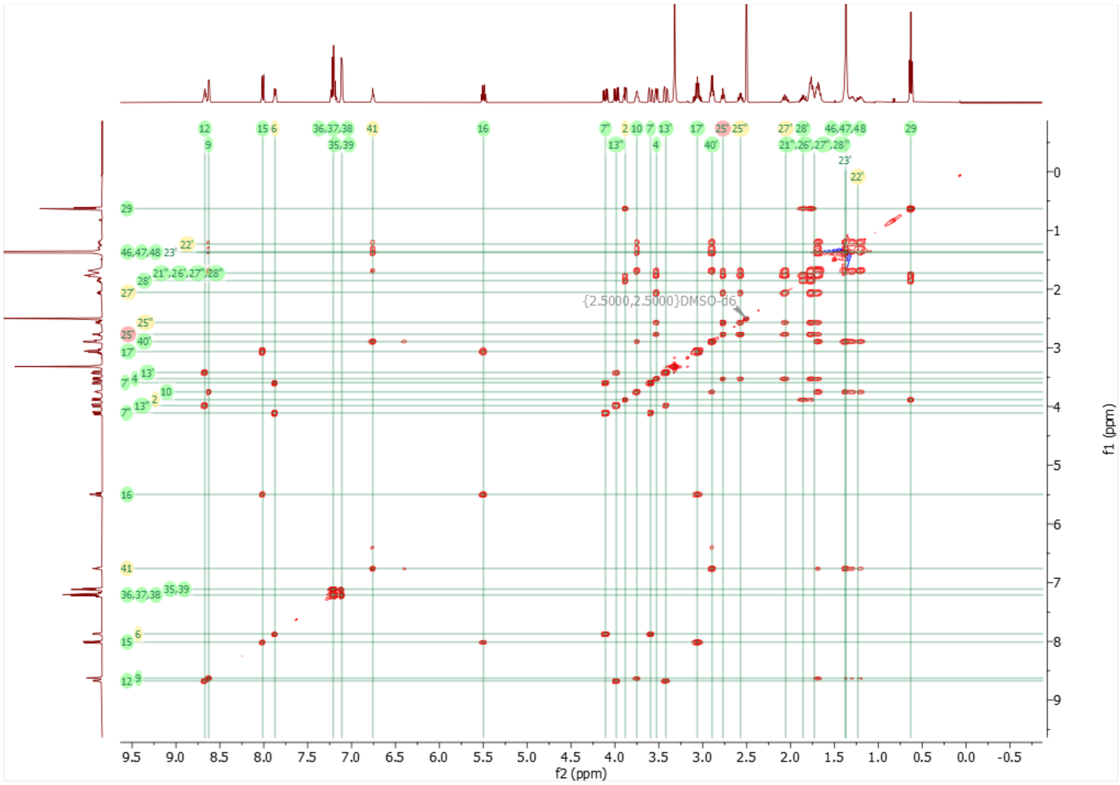

ROESY at 25°C

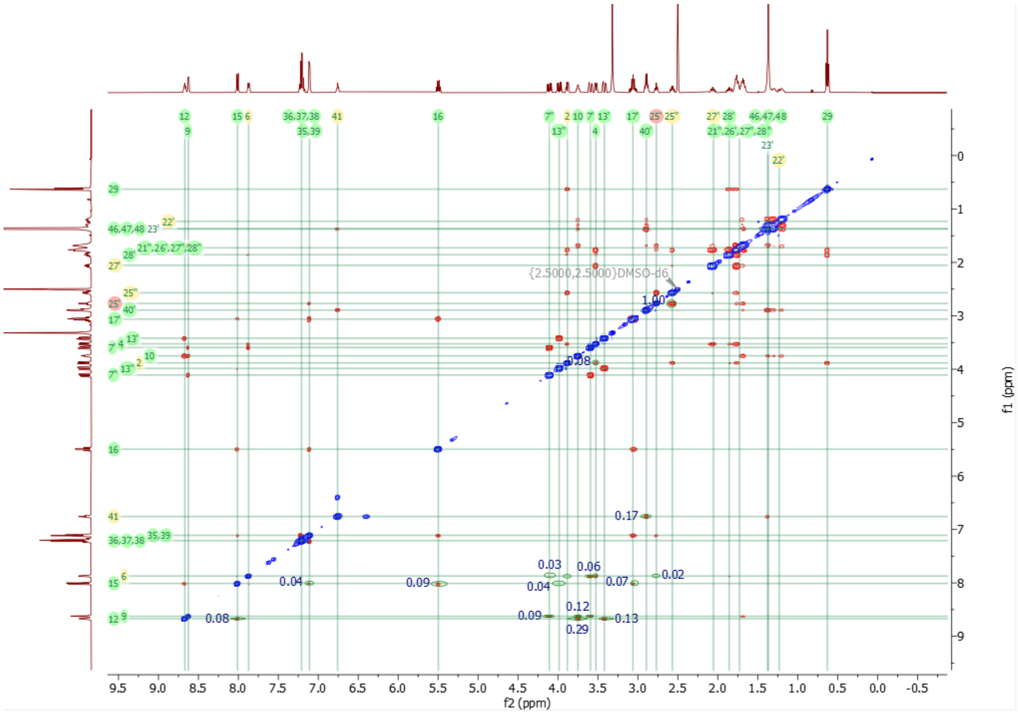

HSQC at 25°C

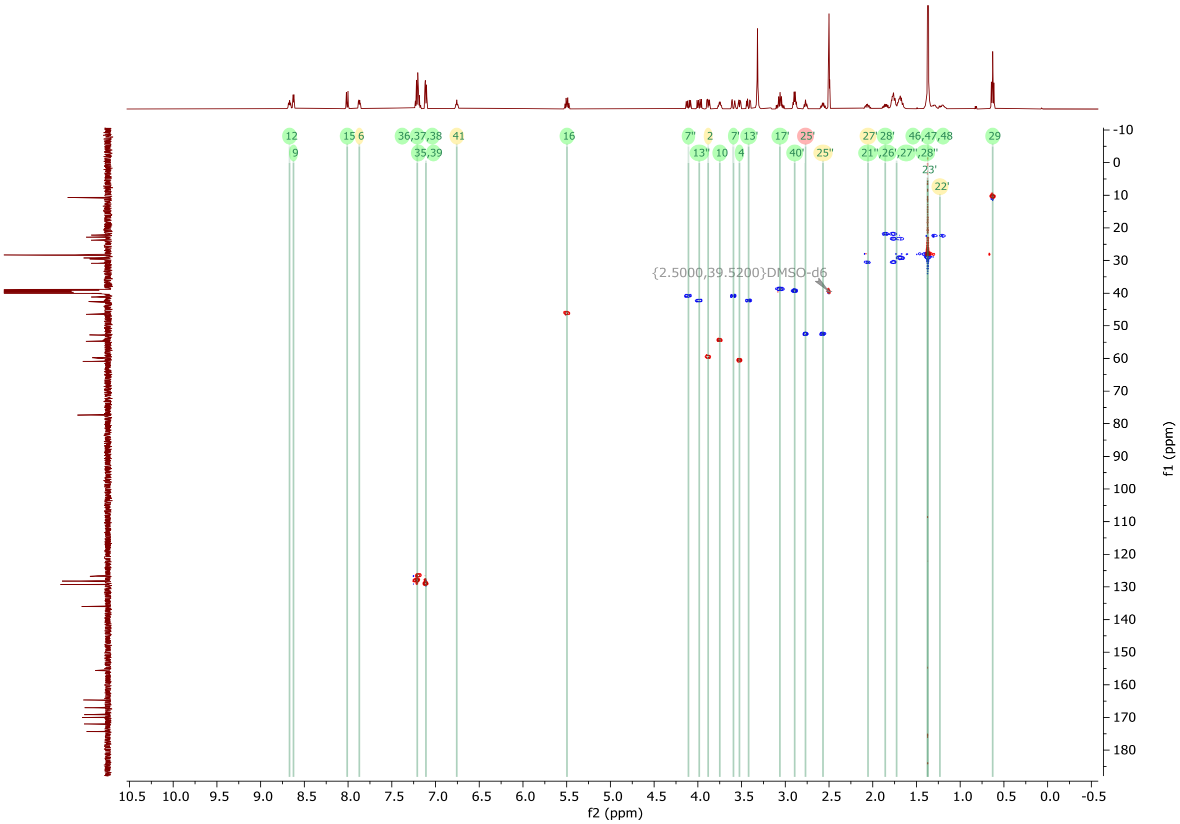

HMBC at 25°C

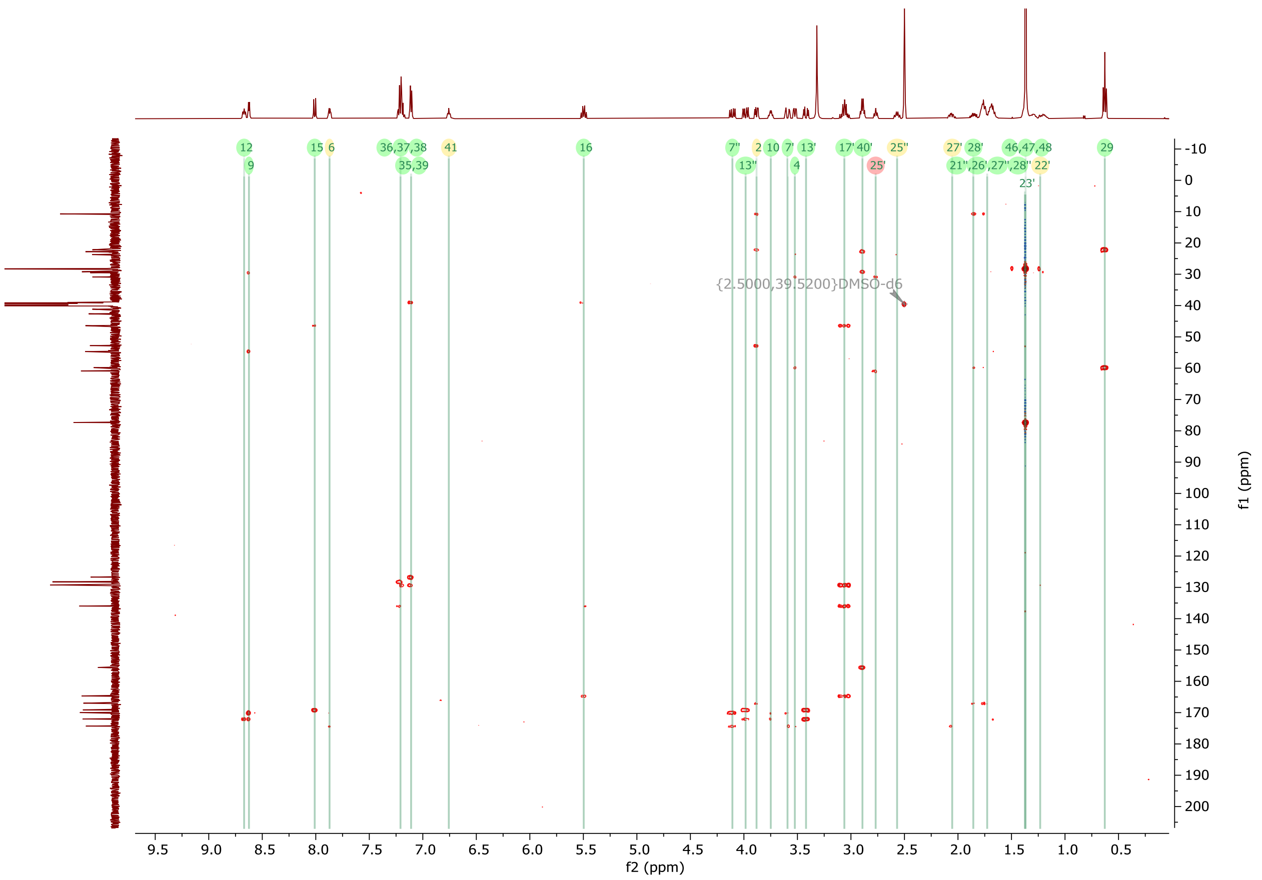

12
9
15
6
36,37,38
35,39
41
16
7''
13''
2
10
7'
4
13'
17'
40'
25'
25''
27'
21'',26',27'',28''
28'
46,47,48
22'
23'
29
{2.5000,39.5200}DMSO-d6
f2 (ppm)
f1 (ppm)

cyclo[PK(Boc)LGF] odz/ethyl (3a)

^{1}H NMR (500 MHz, DMSO-d_6) at 25°C

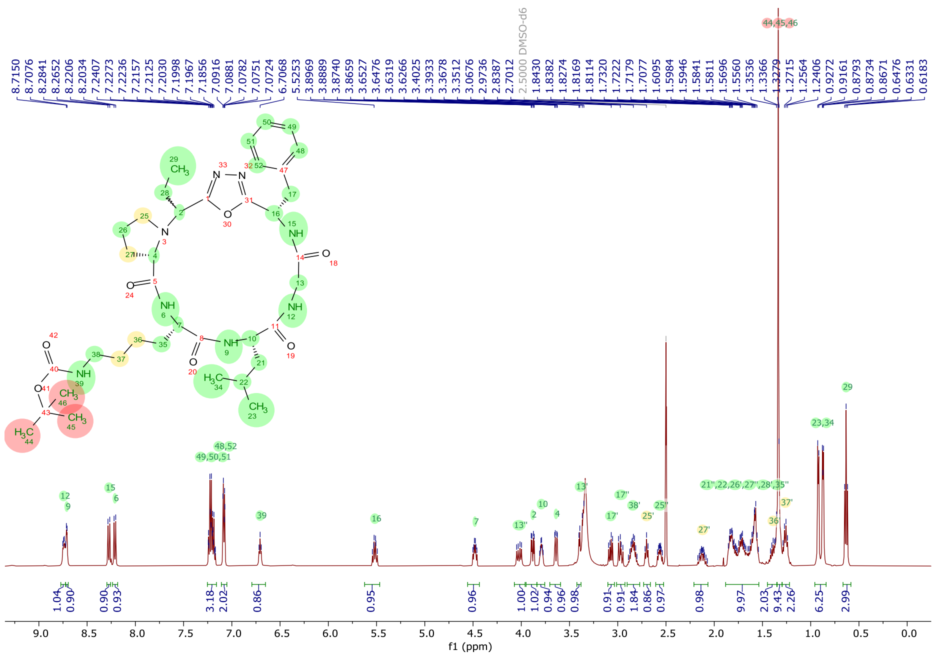

^{13}C NMR (126 MHz, DMSO-d_6) at 25°C

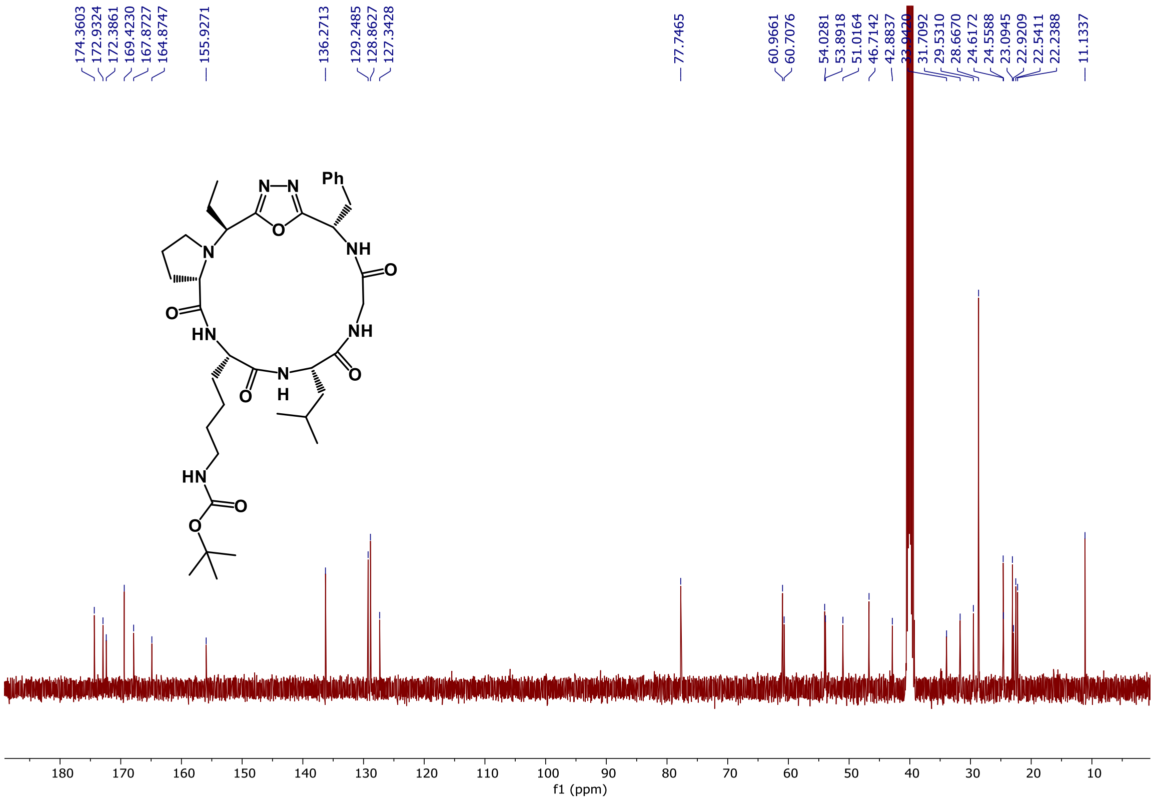

COSY at 25°C

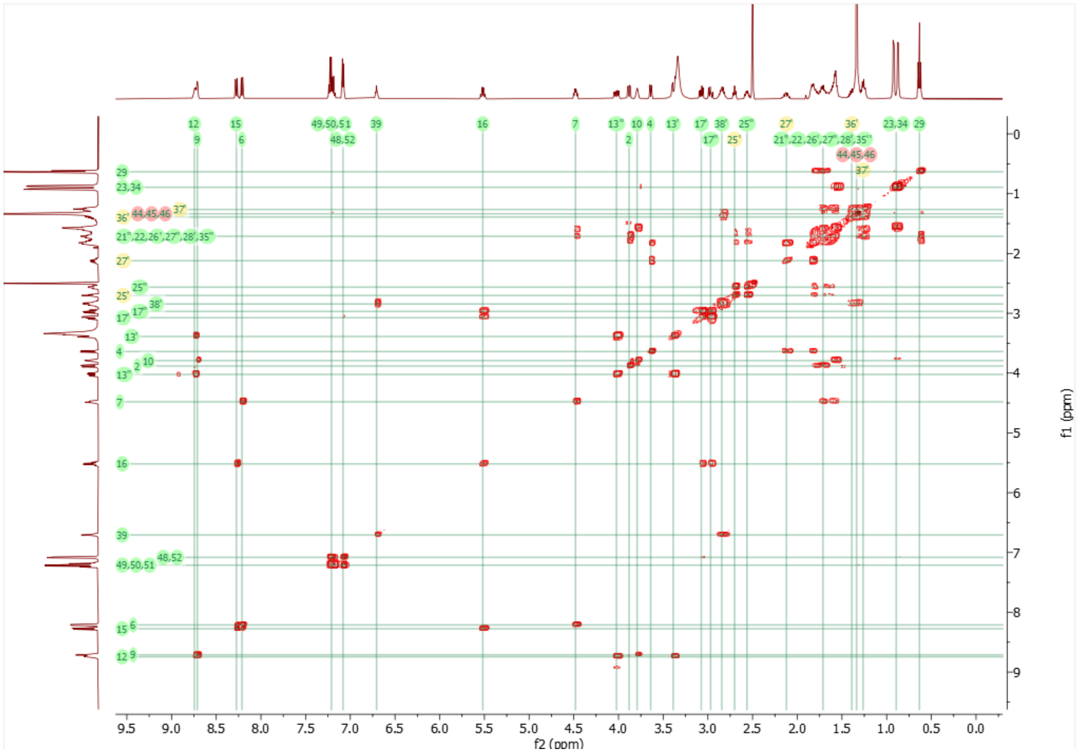

TOCSY at 25°C

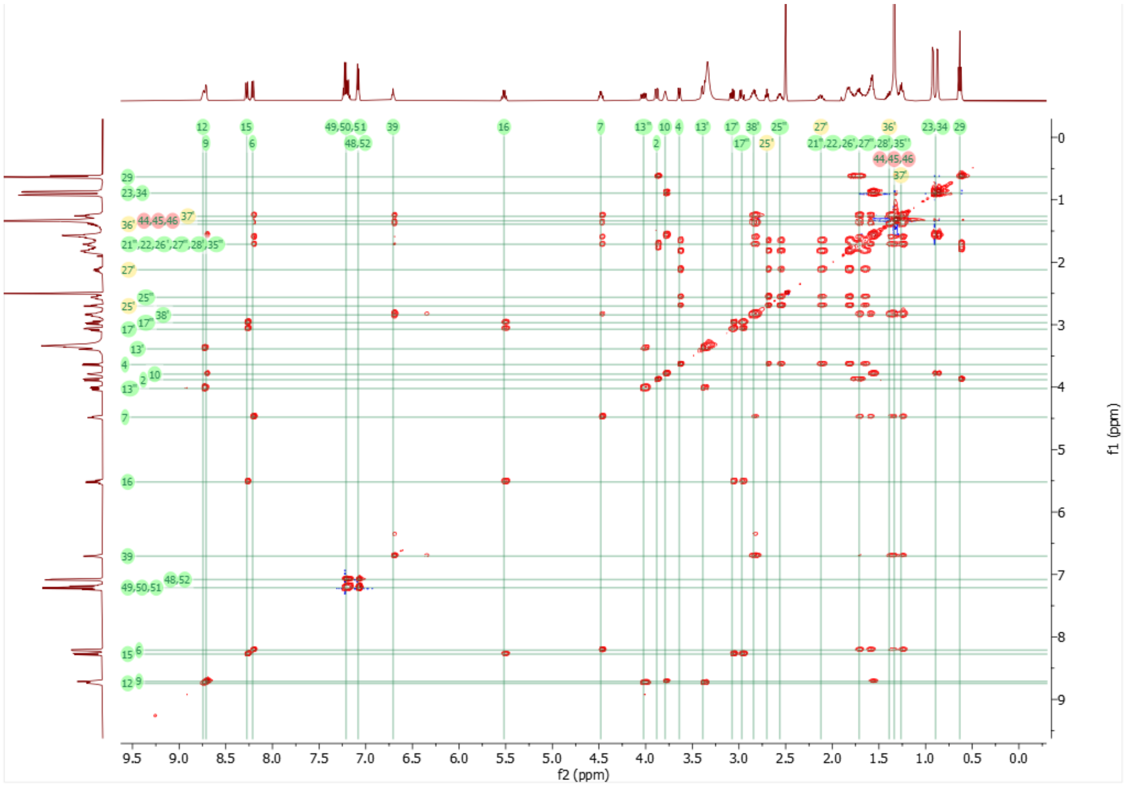

ROESY at 25°C

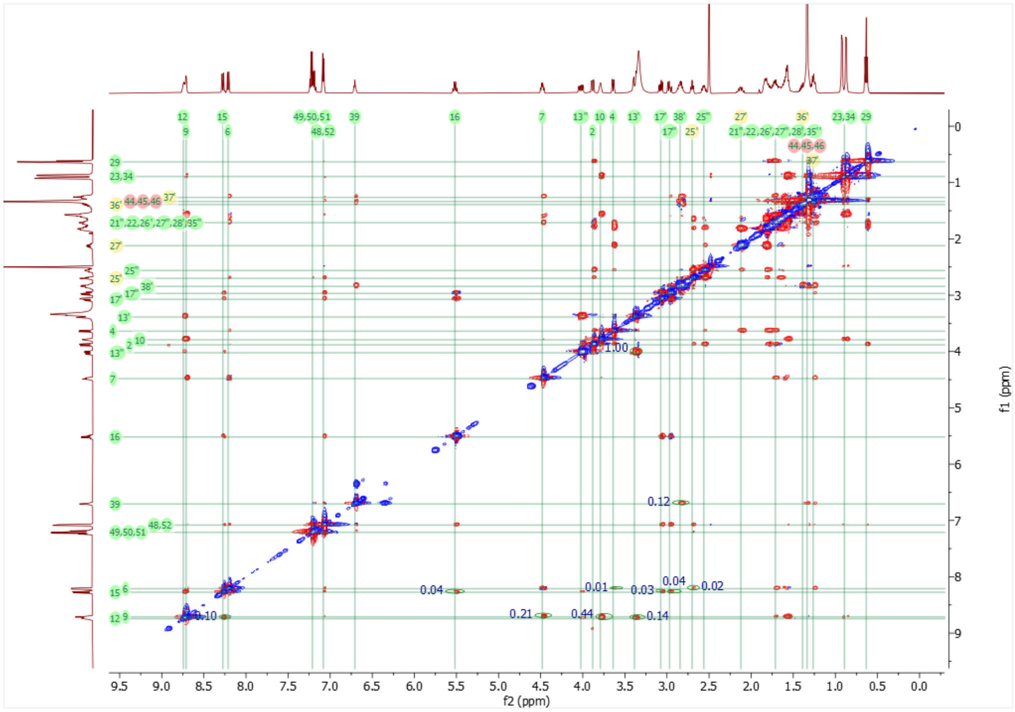

HSQC at 25°C

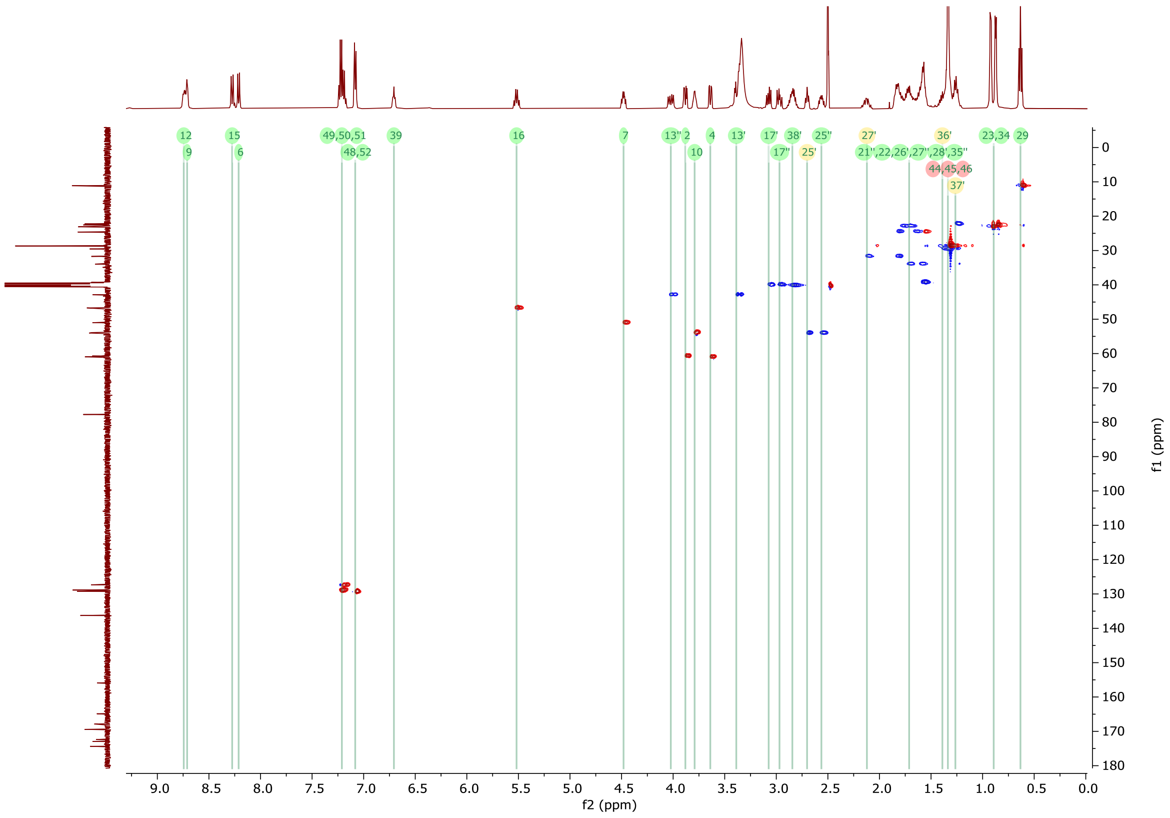

HMBC at 25°C

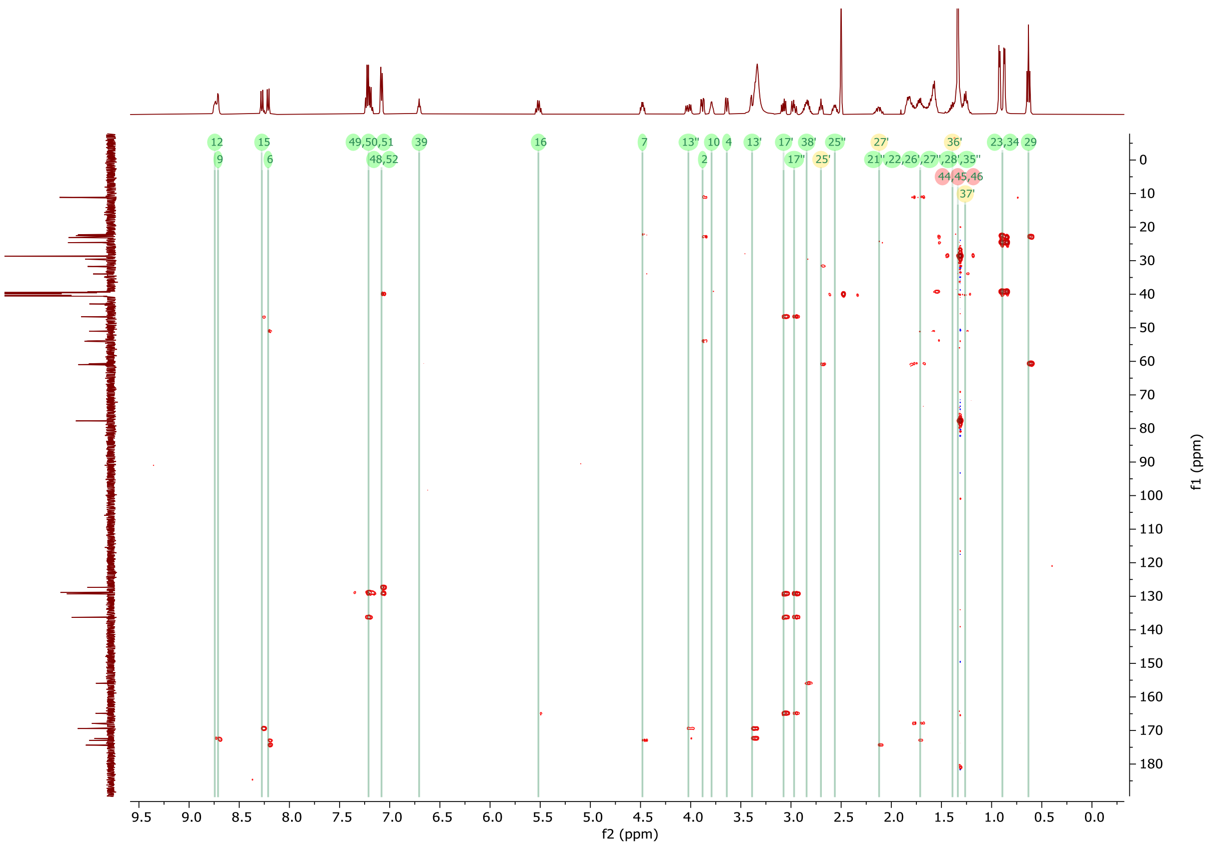

12
9
15
6
49,50,51
48,52
39
16
7
13''
2
10
4
13'
17'
17''
38'
25'
25''
27'
21'',22,26',27'',28',35''
36'
44,45,46
37'
23,34
29
f2 (ppm)
f1 (ppm)

cyclo[K(Boc)GLGF] odz/ethyl (4a)

^{1}H NMR (500 MHz, DMSO-d_6) at 25°C

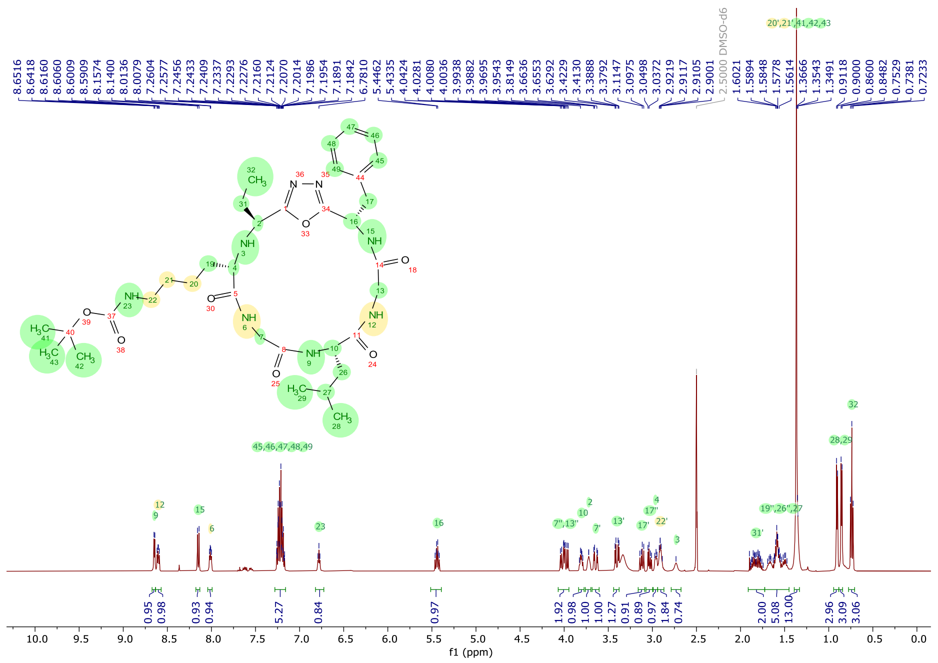

^{13}C NMR (126 MHz, DMSO-d_6) at 25°C

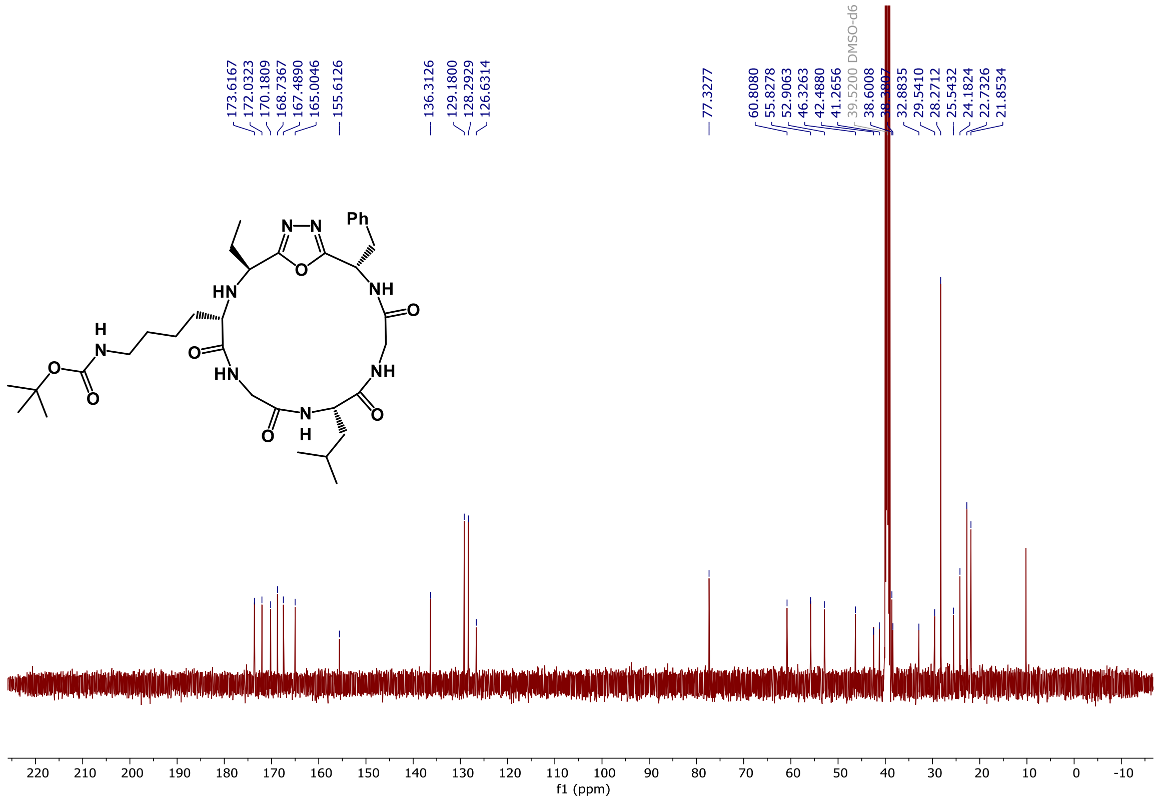

COSY at 25°C

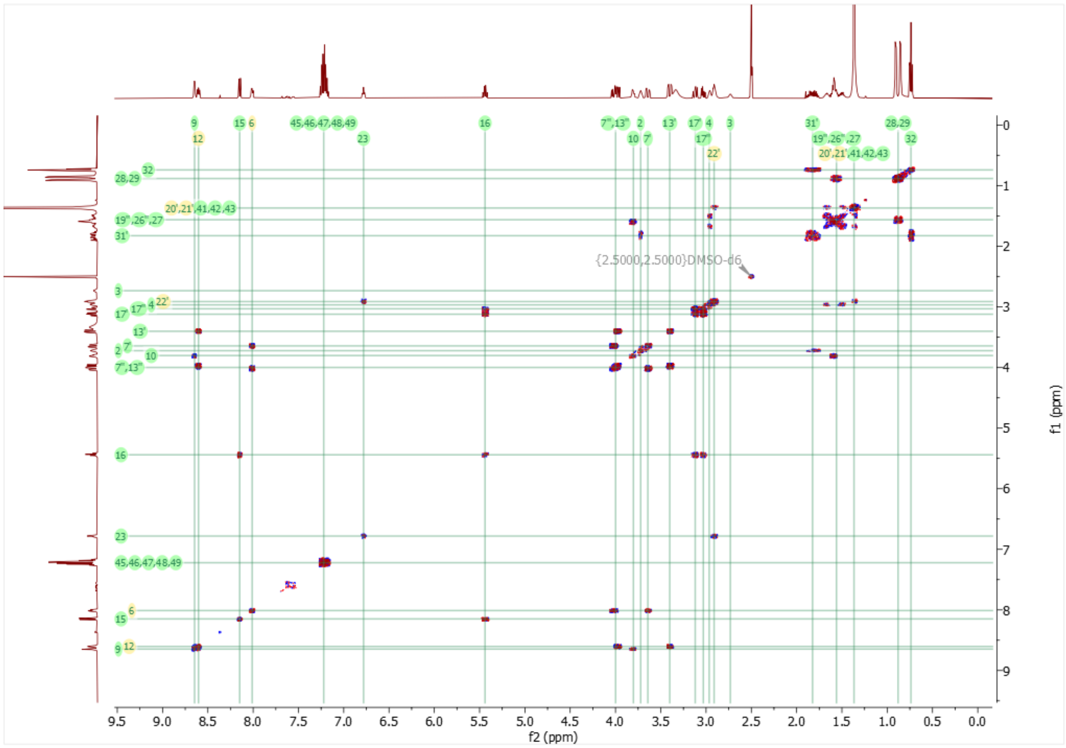

TOCSY at 25°C

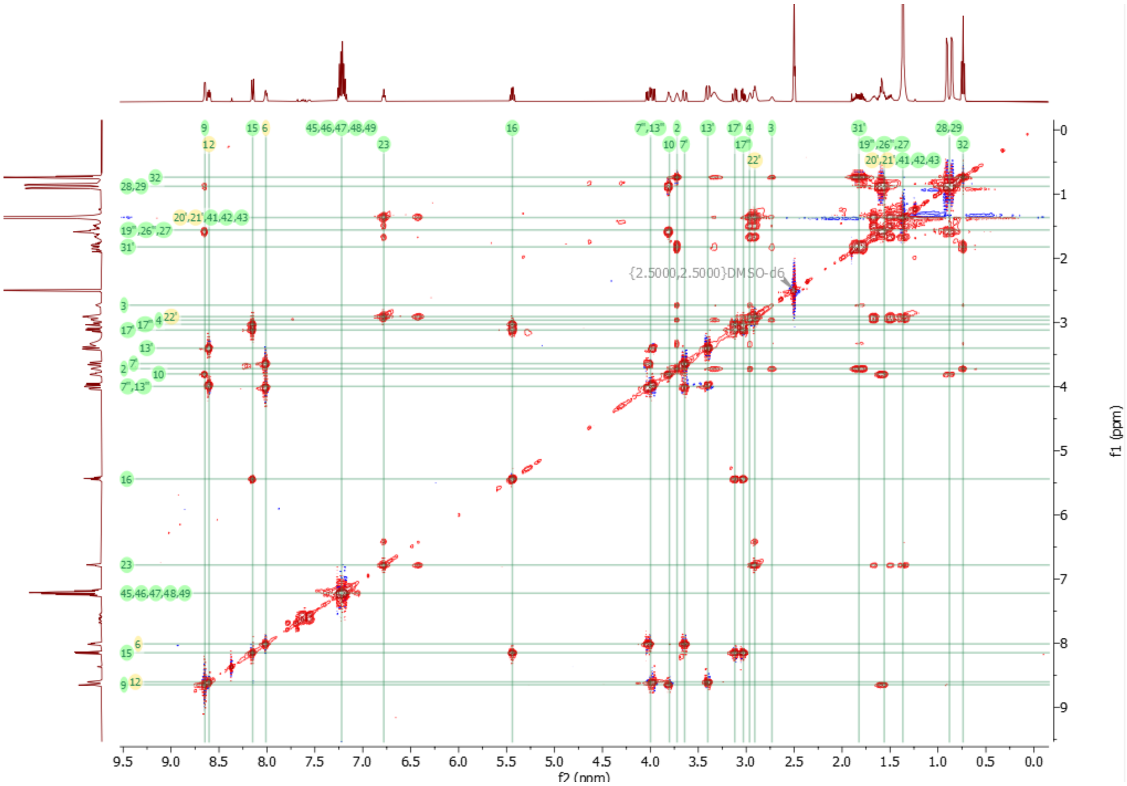

ROESY at 25°C

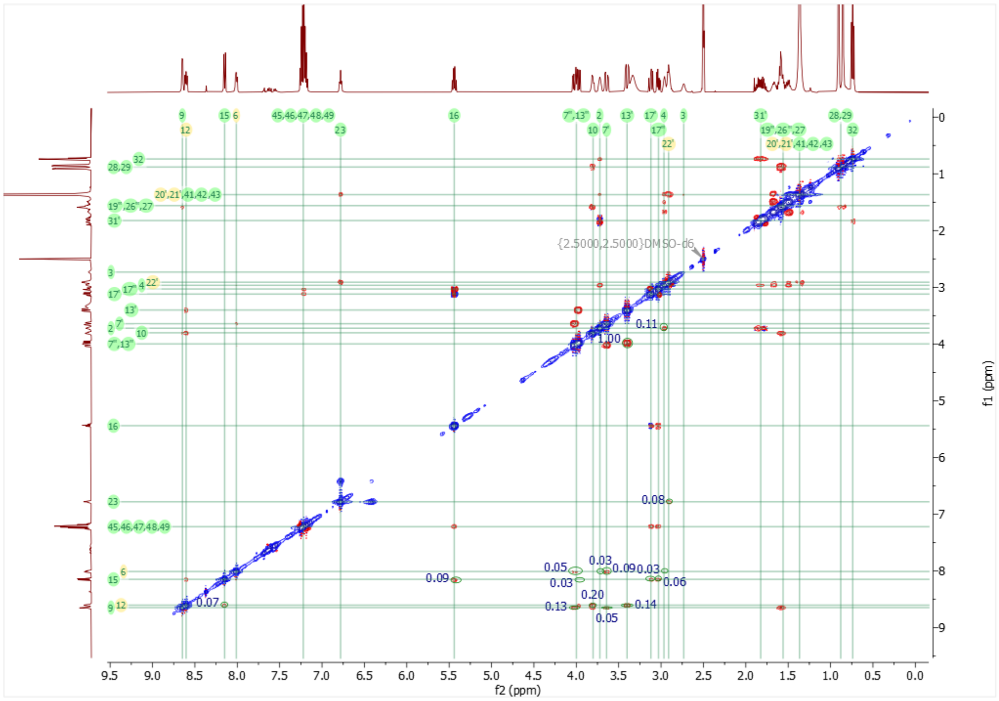

HSQC at 25°C

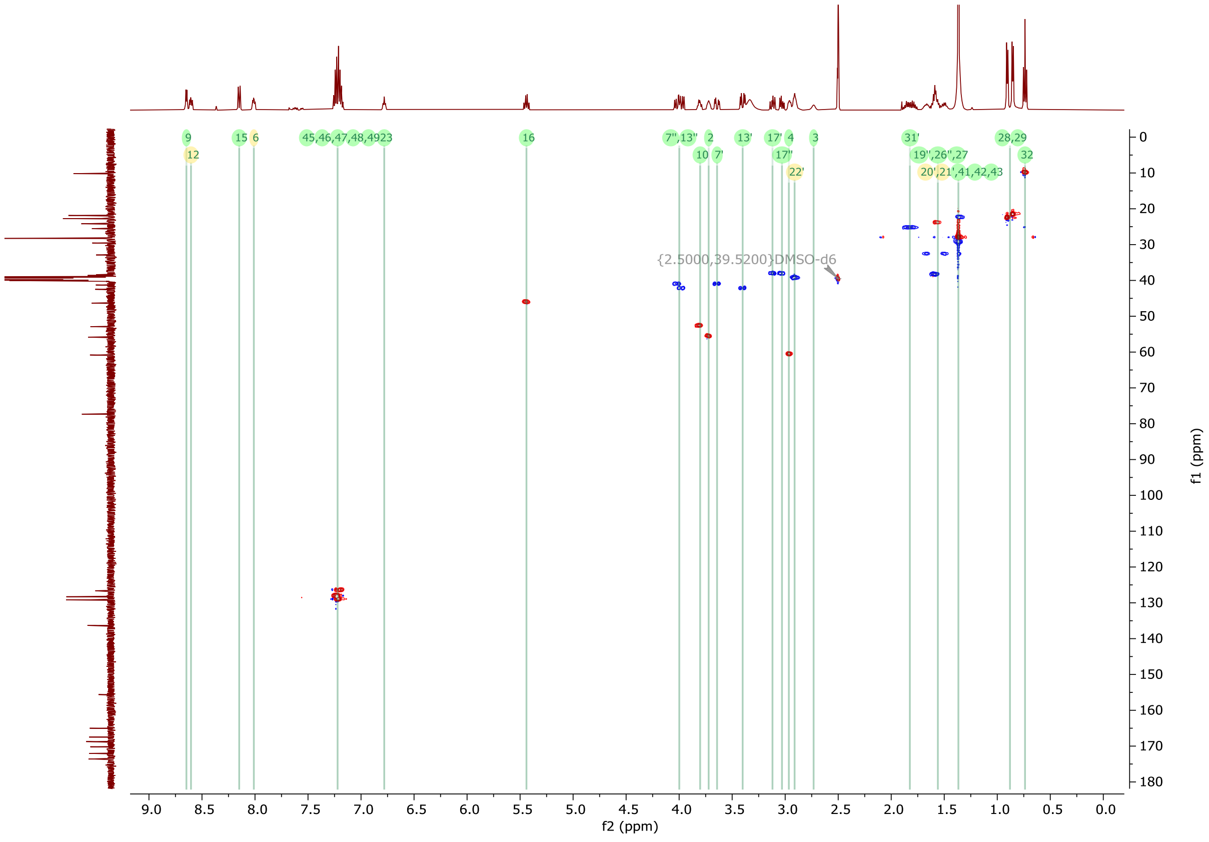

HMBC at 25°C

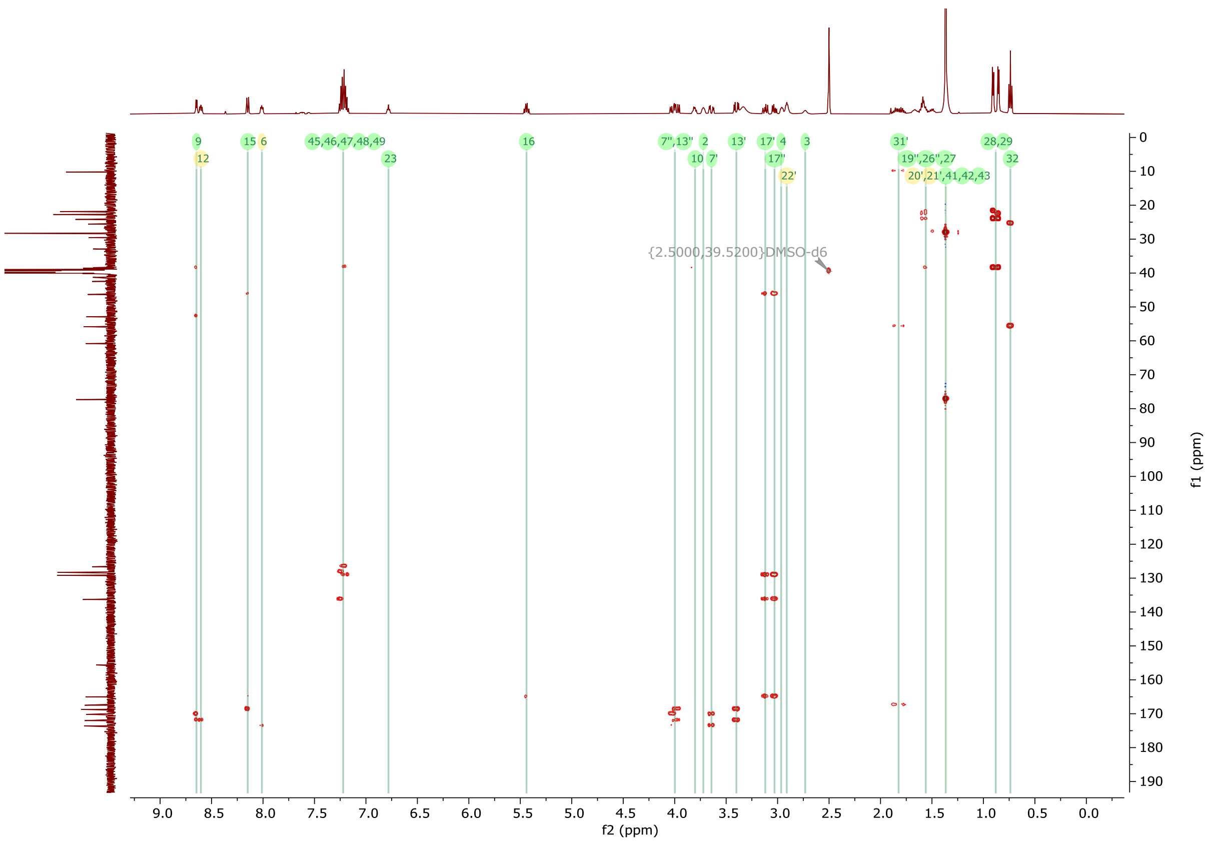

9
12
15
6
45,46,47,48,49
23
16
7'',13''
2
10
7'
13'
17'
4
17''
22'
3
31'
19'',26'',27
20',21',41,42,43
28,29
32
{2.5000,39.5200}DMSO-d6
f2 (ppm)
f1 (ppm)

cyclo[PGLGK(Boc)] odz/ethyl (5a)

^{1}H NMR (500 MHz, DMSO-d_6) at 25°C

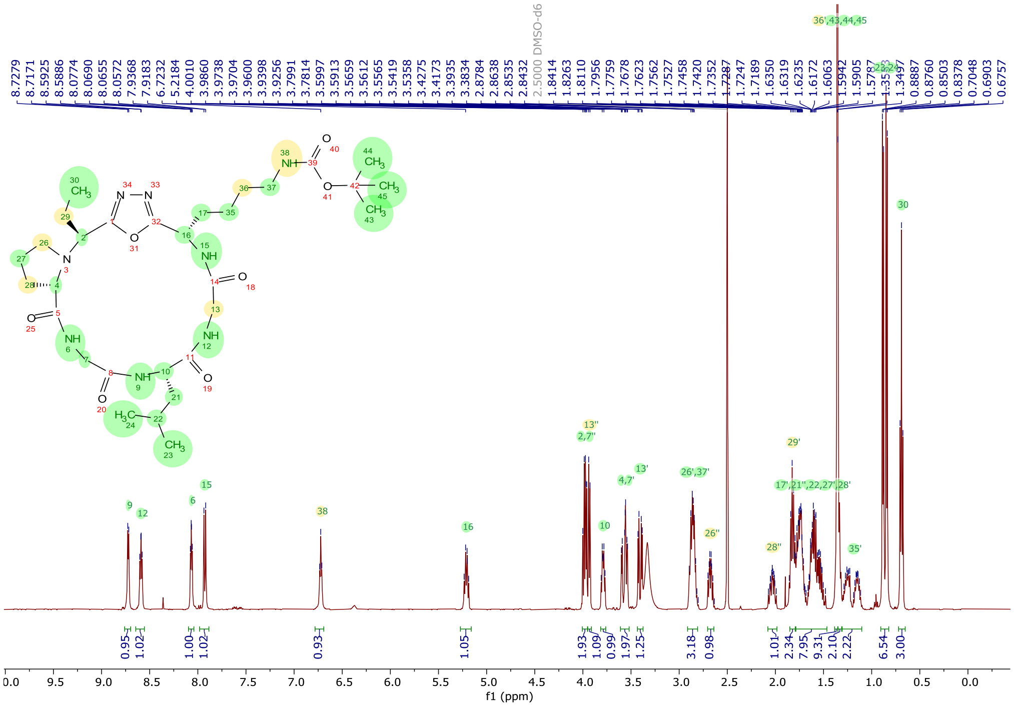

^{13}C NMR (126 MHz, DMSO-d_6) at 25°C

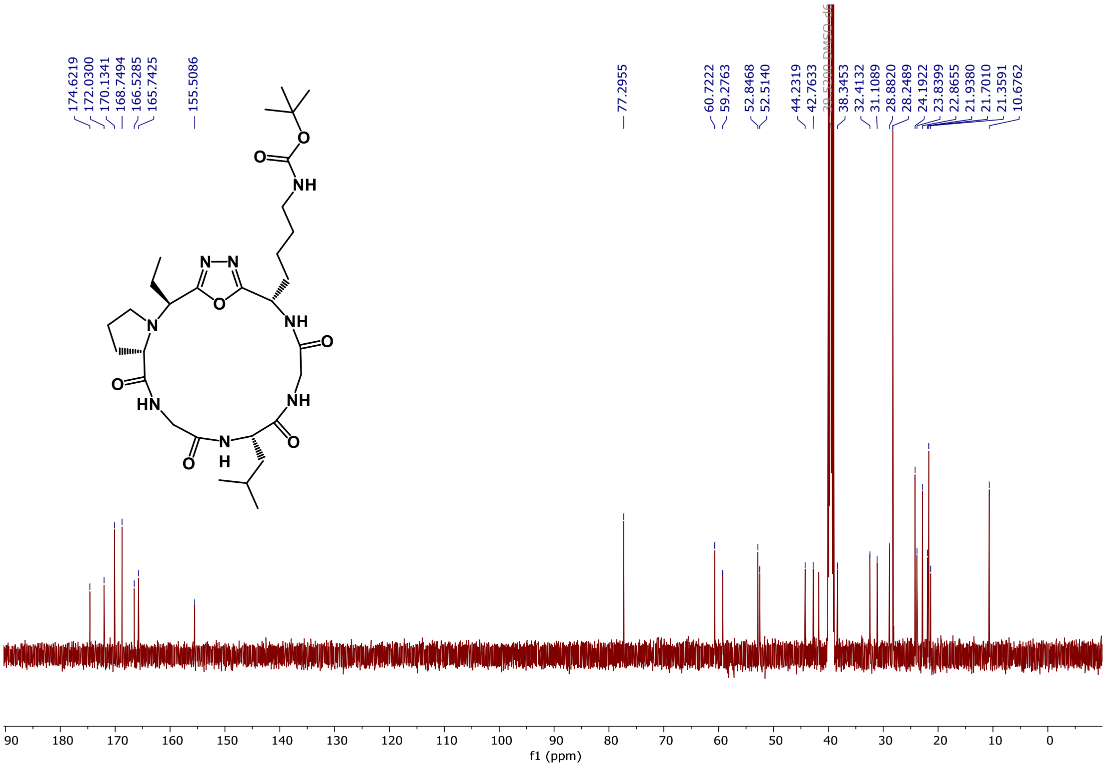

COSY at 25°C

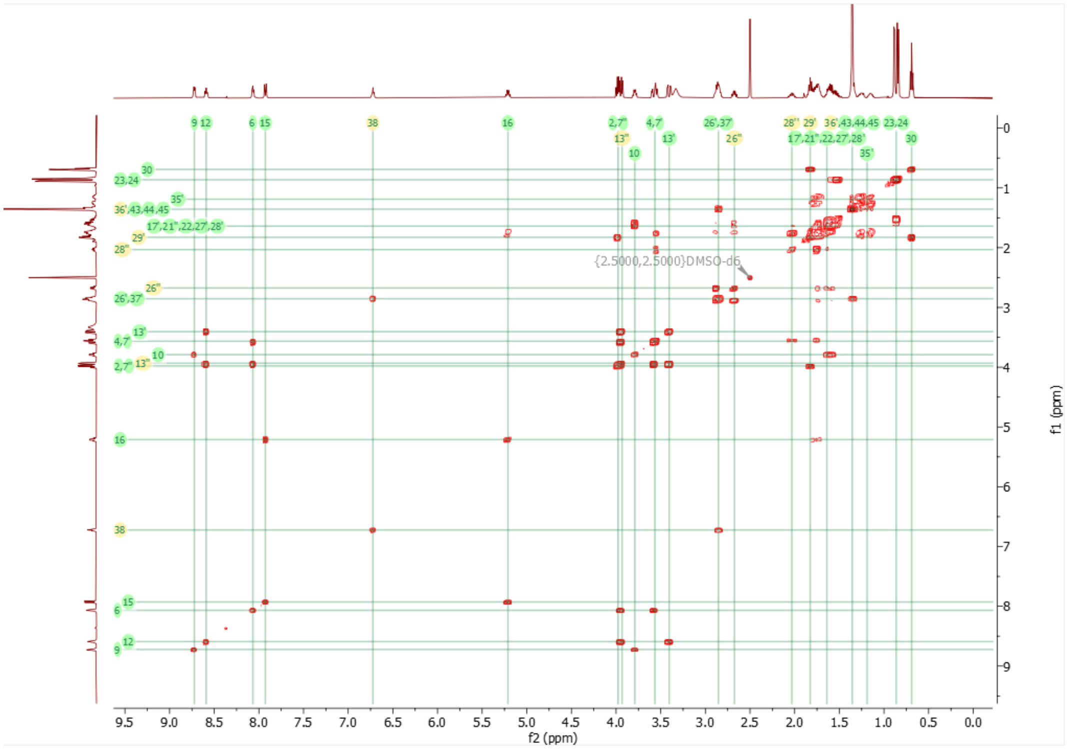

TOCSY at 25°C

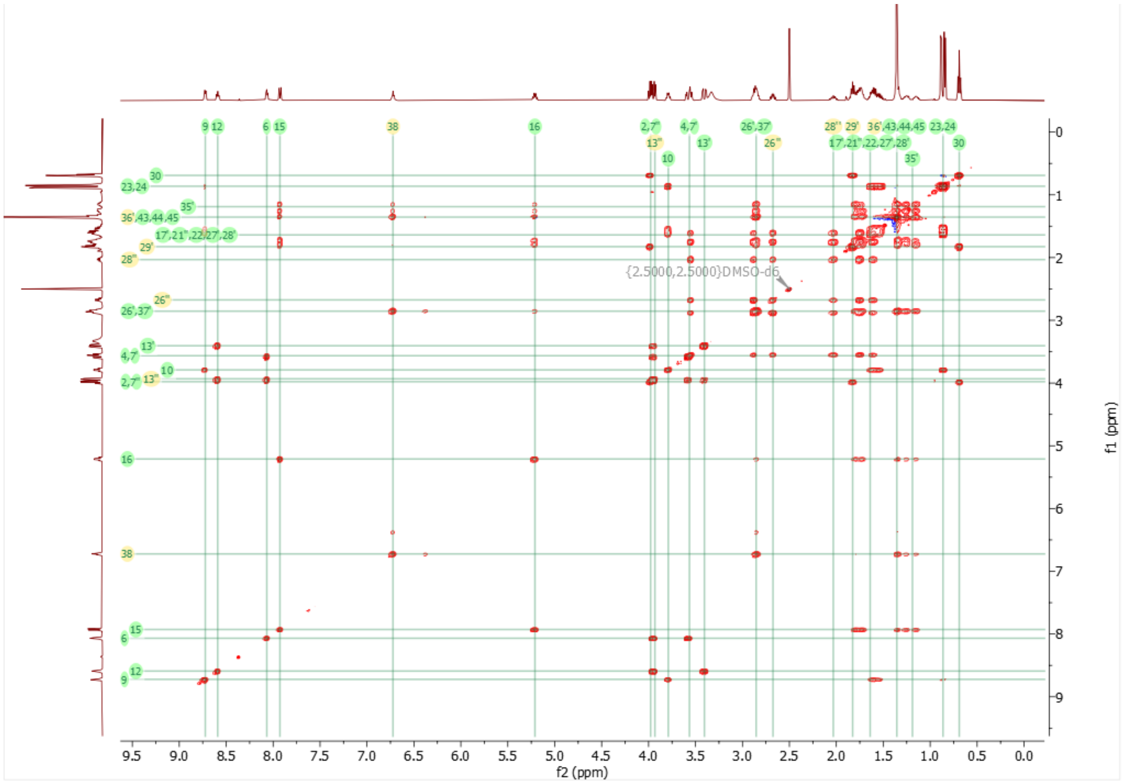

ROESY at 25°C

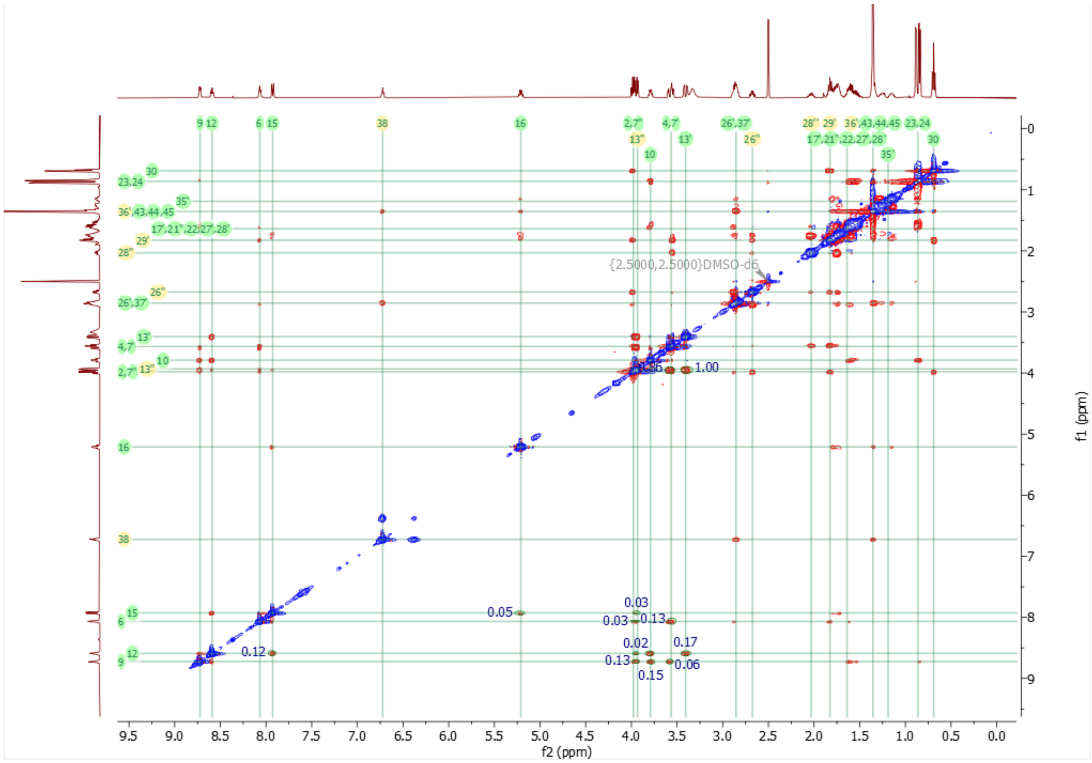

HSQC at 25°C

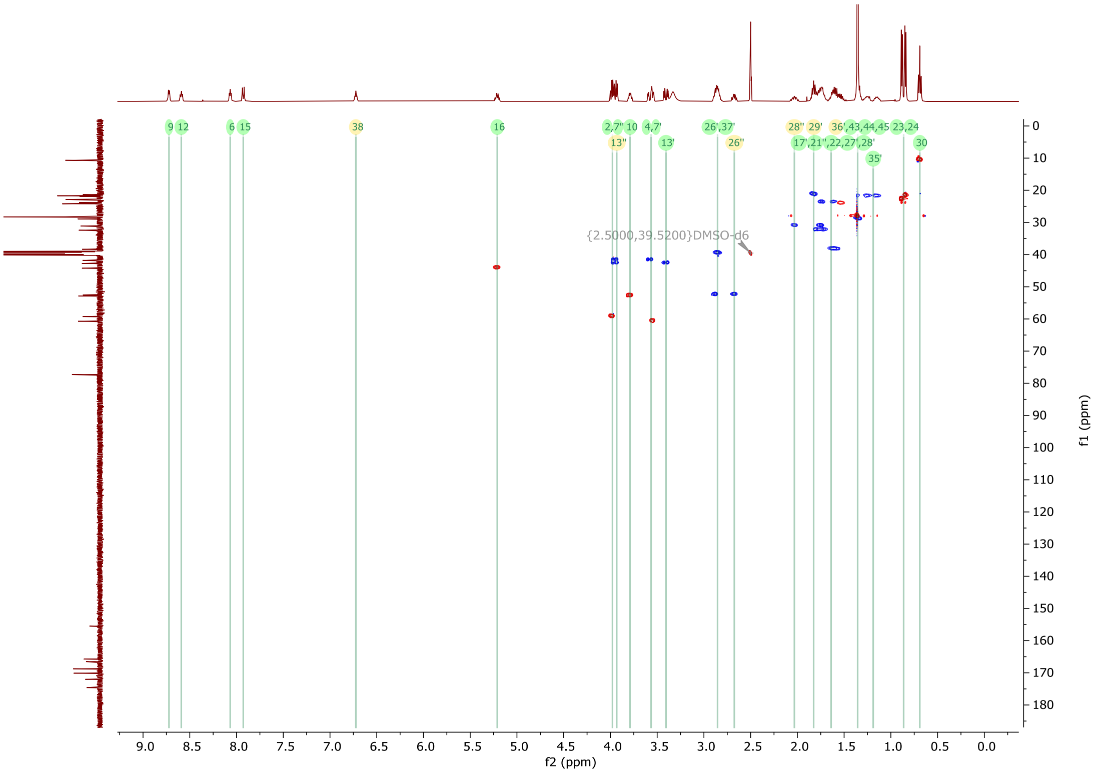

HMBC at 25°C

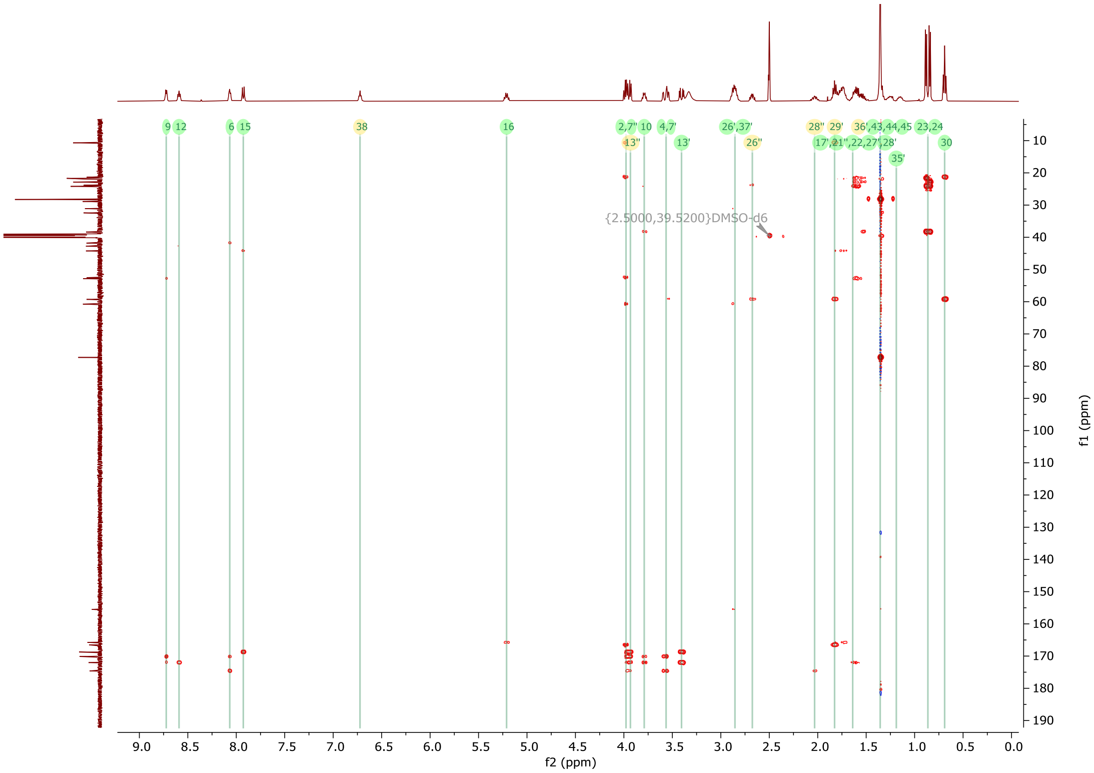

cyclo[PD(tBu)LK(Dde)F] odz/ethyl (6a)

^{1}H NMR (500 MHz, DMSO-d_6) at 25°C

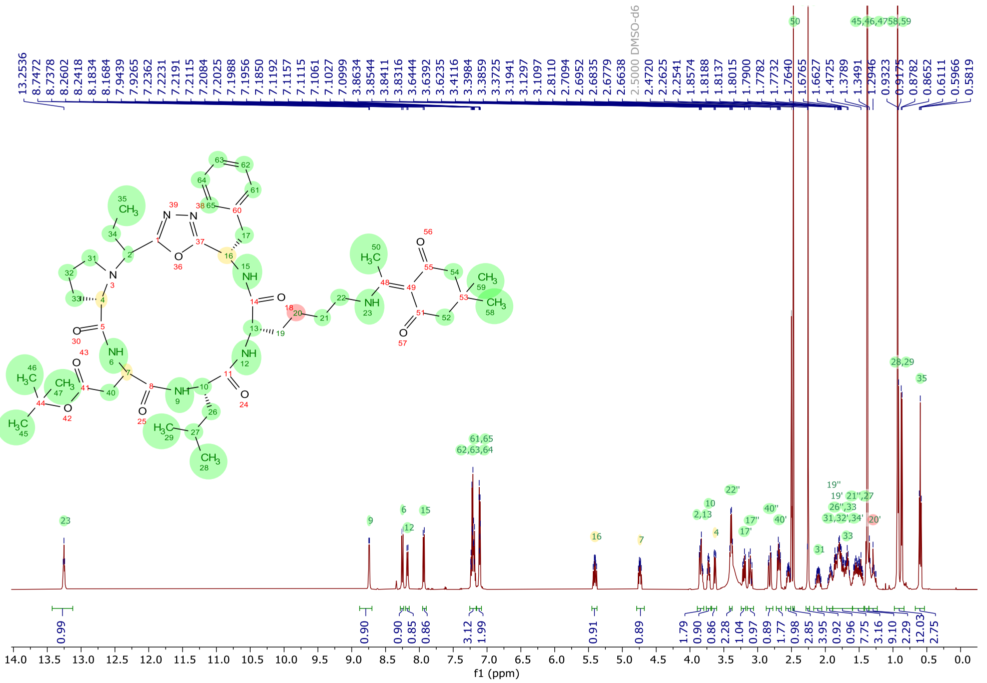

^{13}C NMR (126 MHz, DMSO-d_6) at 25°C

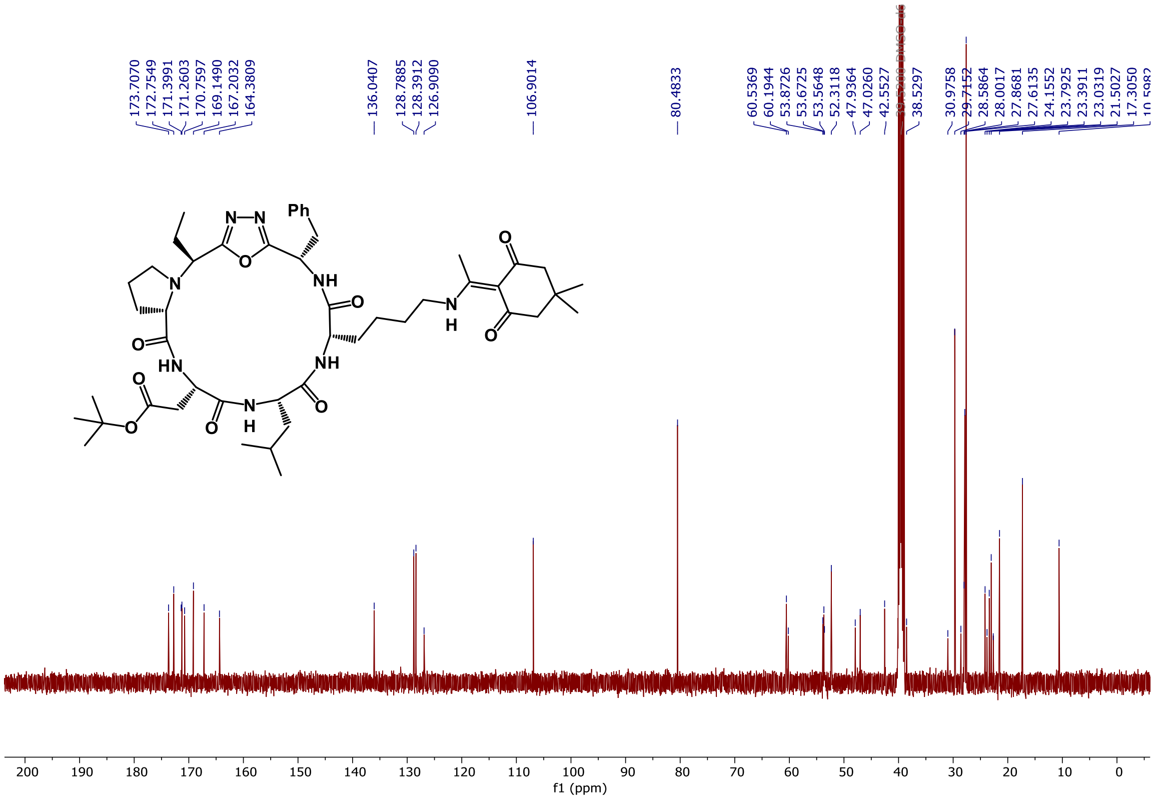

COSY at 25°C

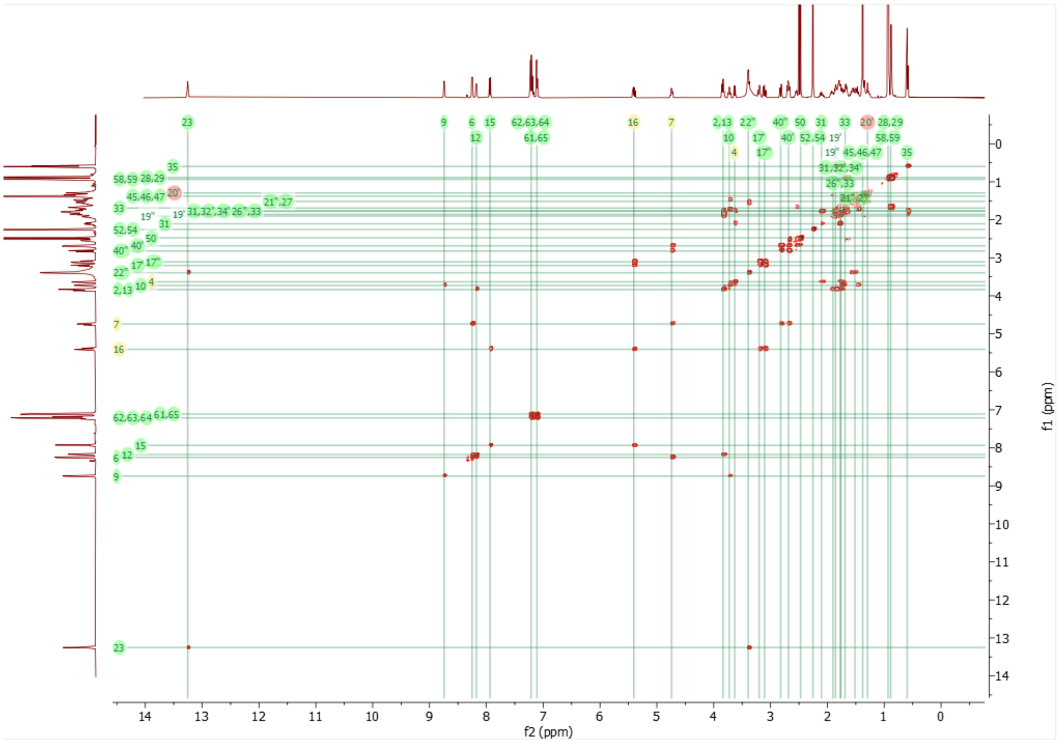

TOCSY at 25°C

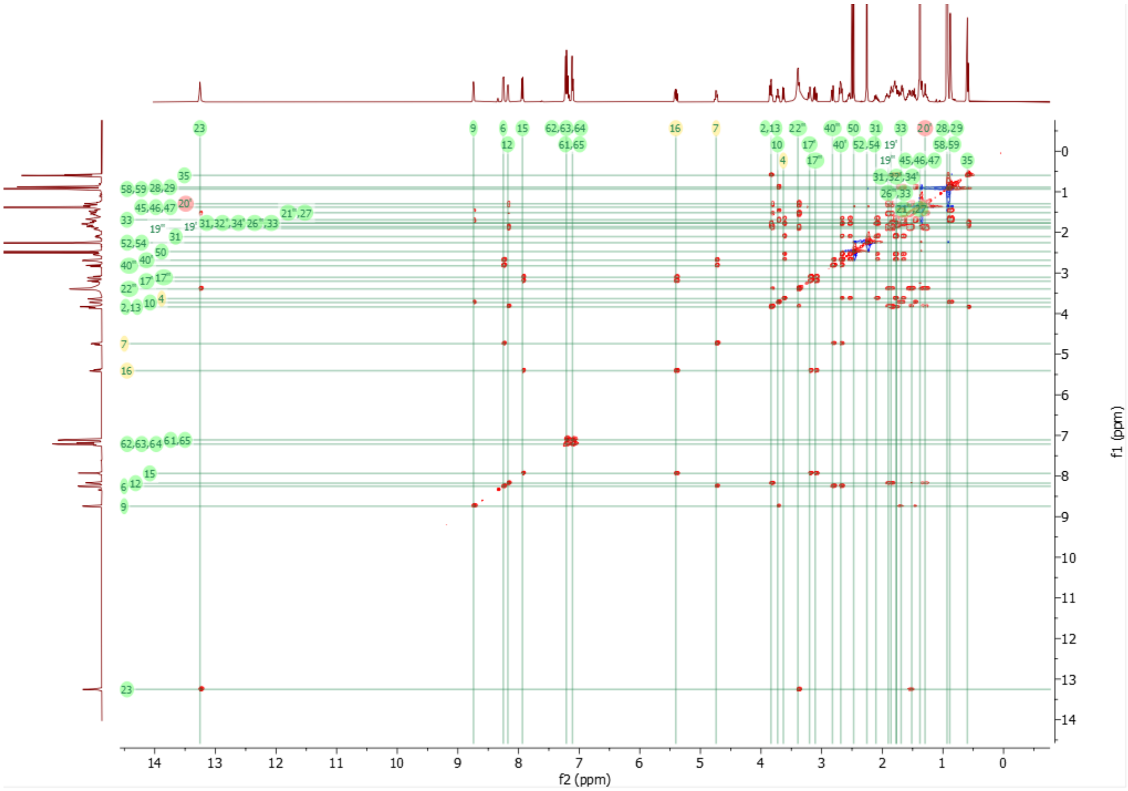

ROESY at 25°C

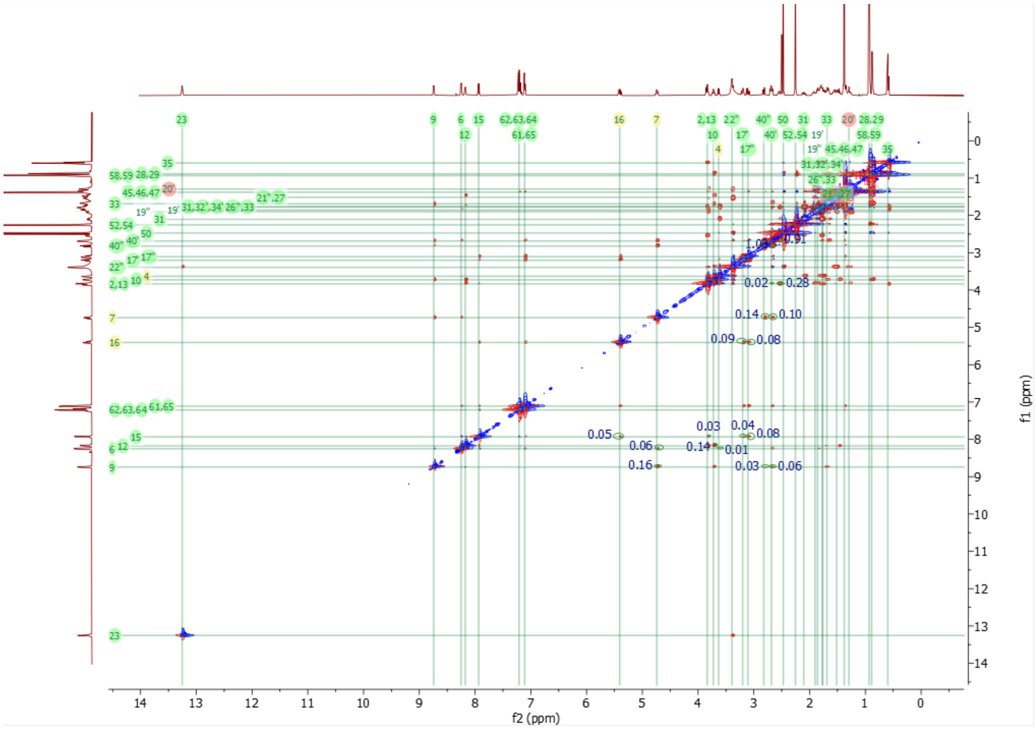

HSQC at 25°C

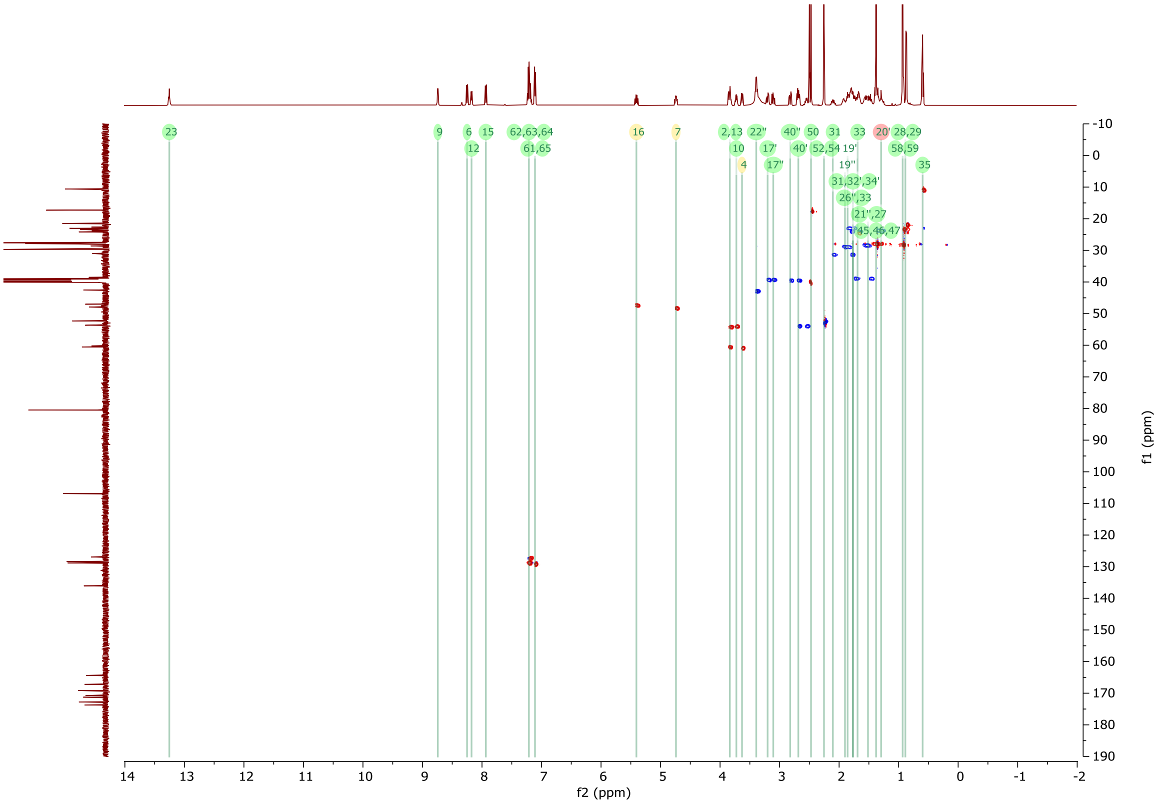

HMBC at 25°C

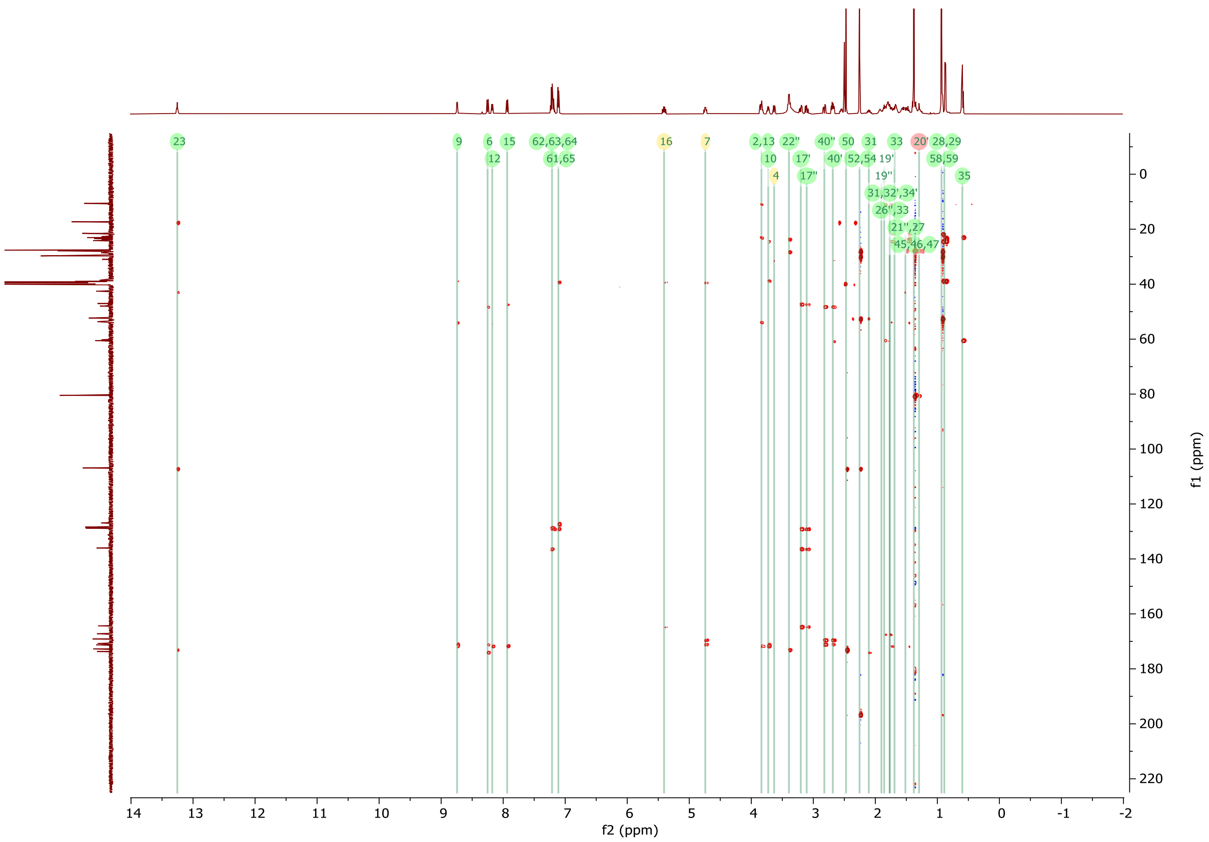

cyclo[PD(tBu)D(tBu)K(Dde)F] odz/ethyl (7a)

^{1}H NMR (500 MHz, DMSO-d_6) at 25°C

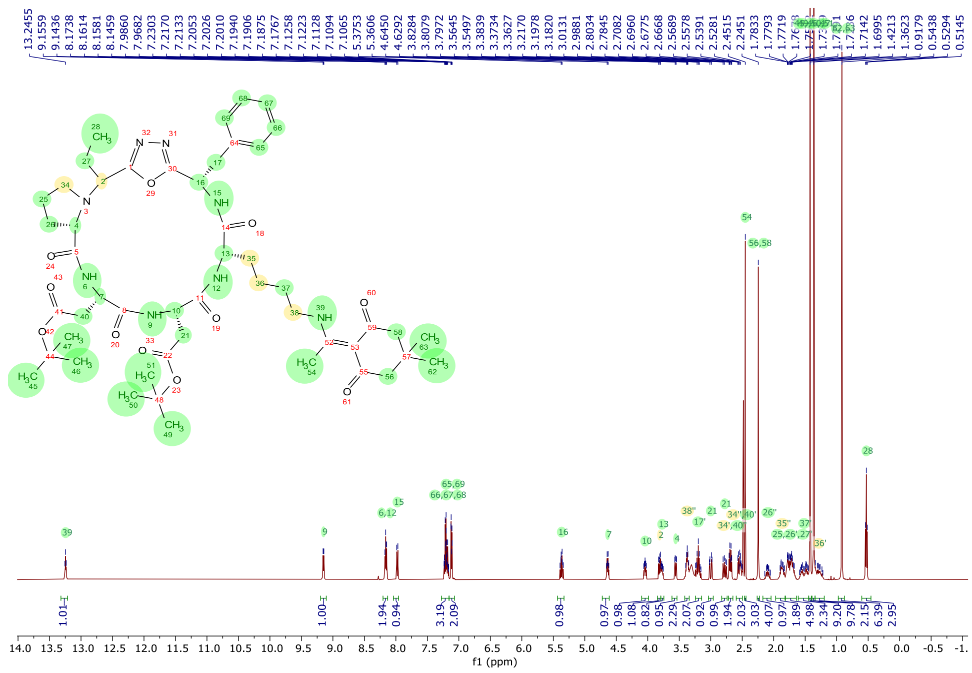

^{13}C NMR (126 MHz, DMSO-d_6) at 25°C

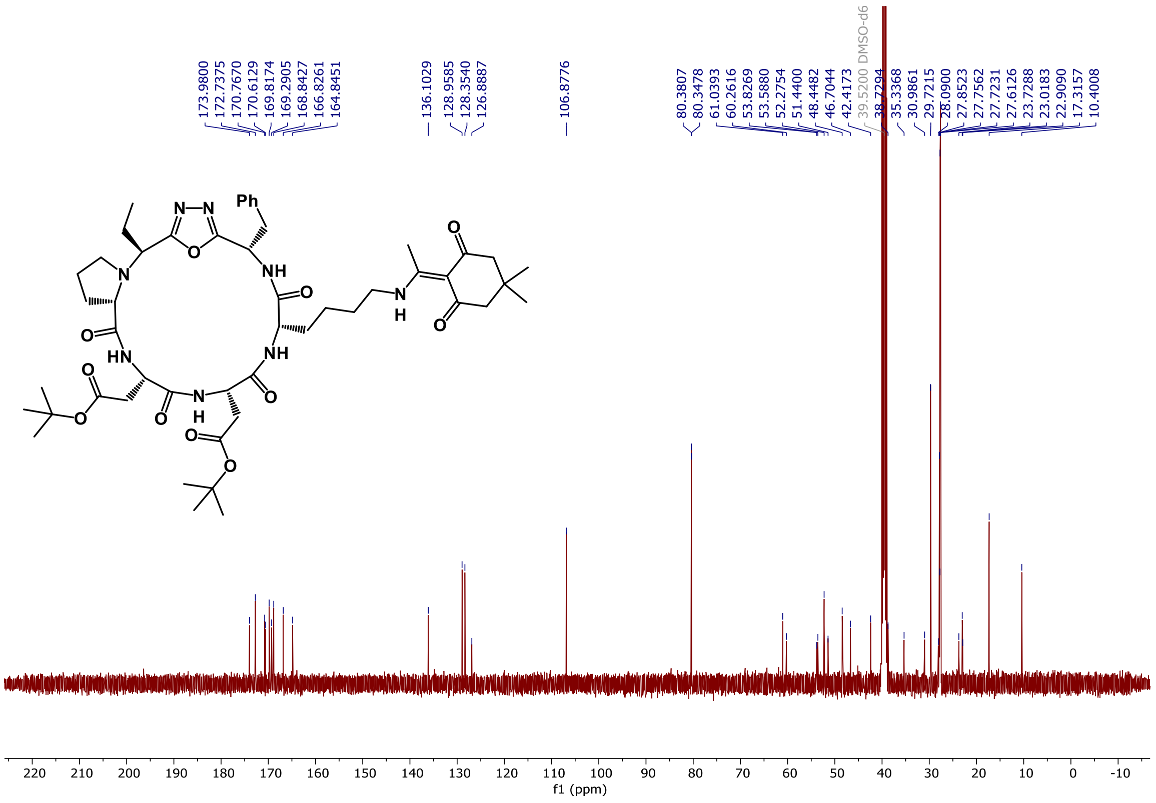

COSY at 25°C

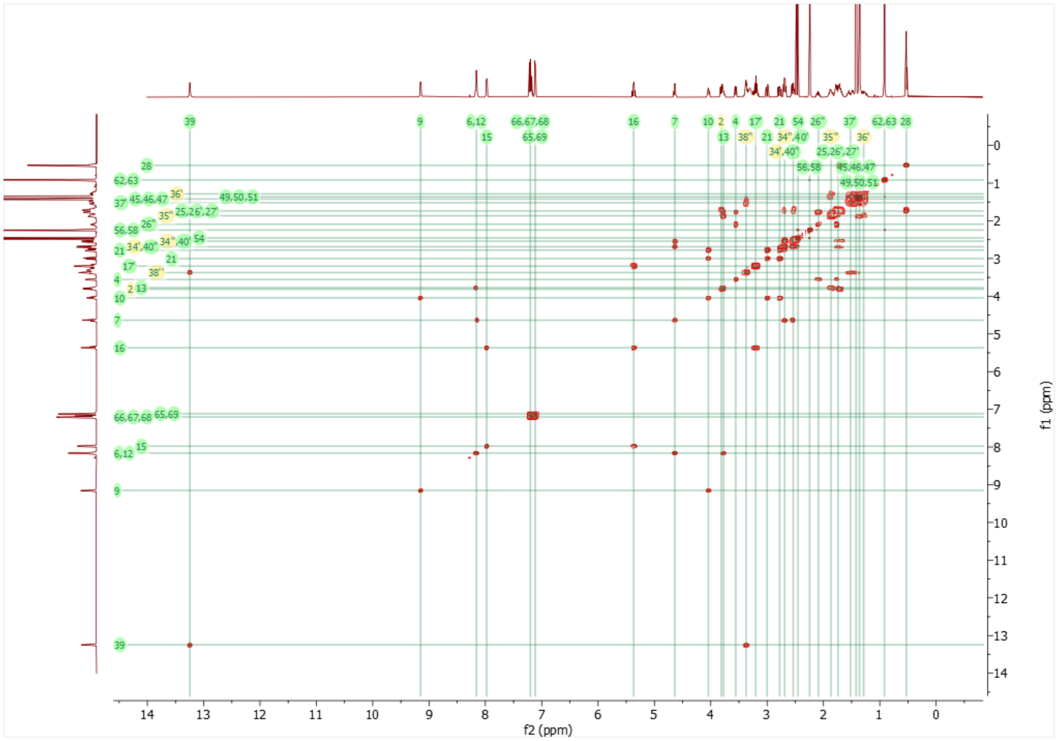

TOCSY at 25°C

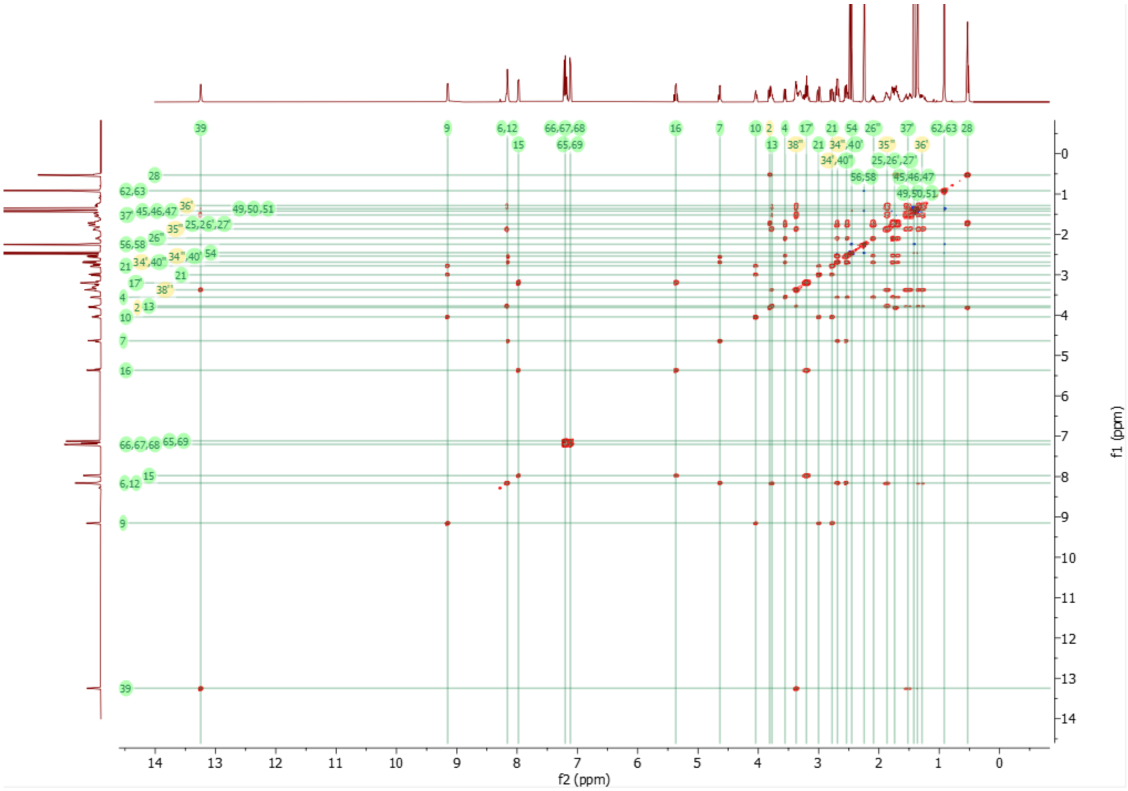

ROESY at 25°C

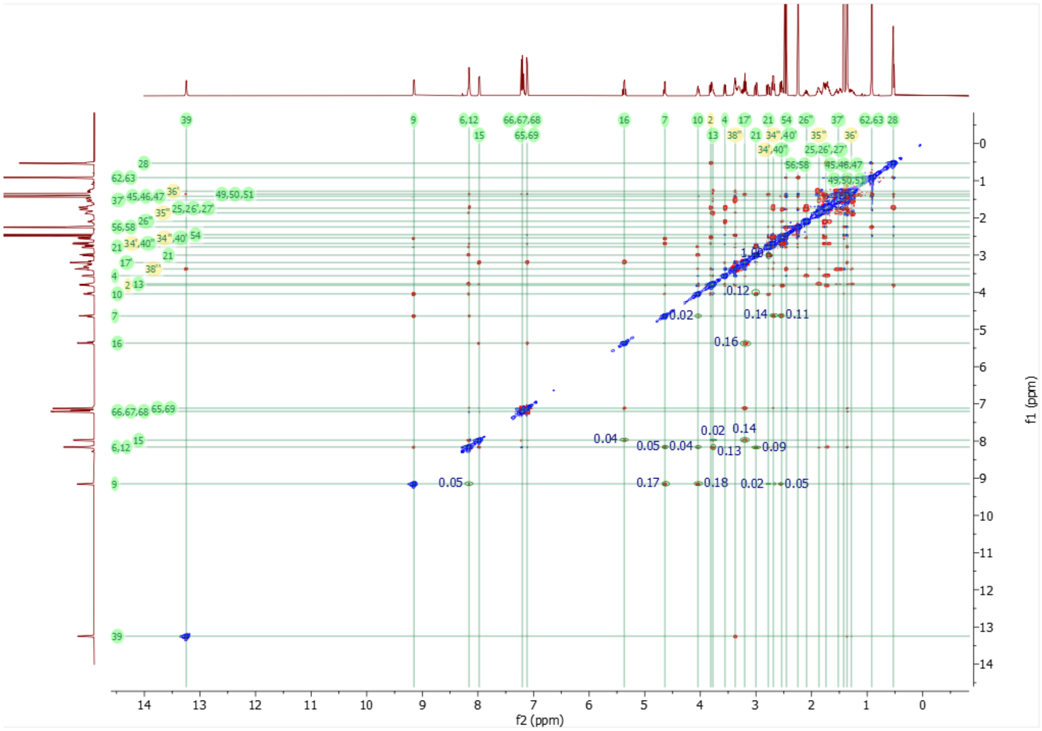

HSQC at 25°C

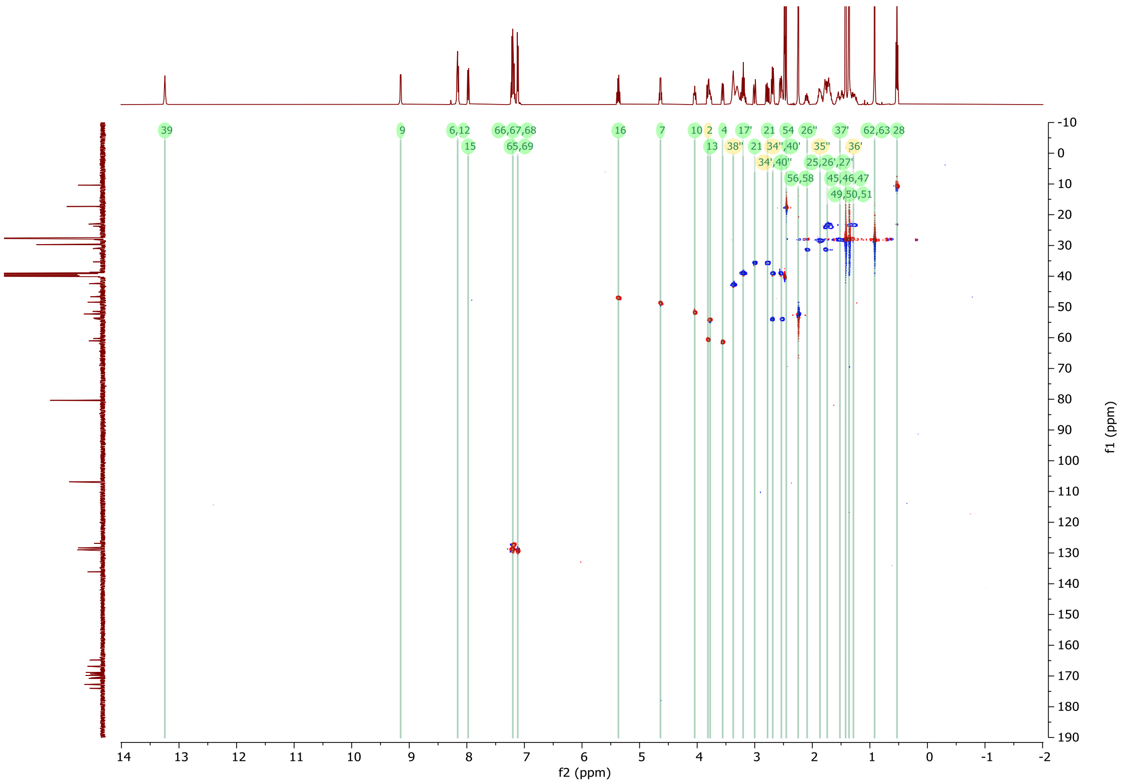

HMBC at 25°C

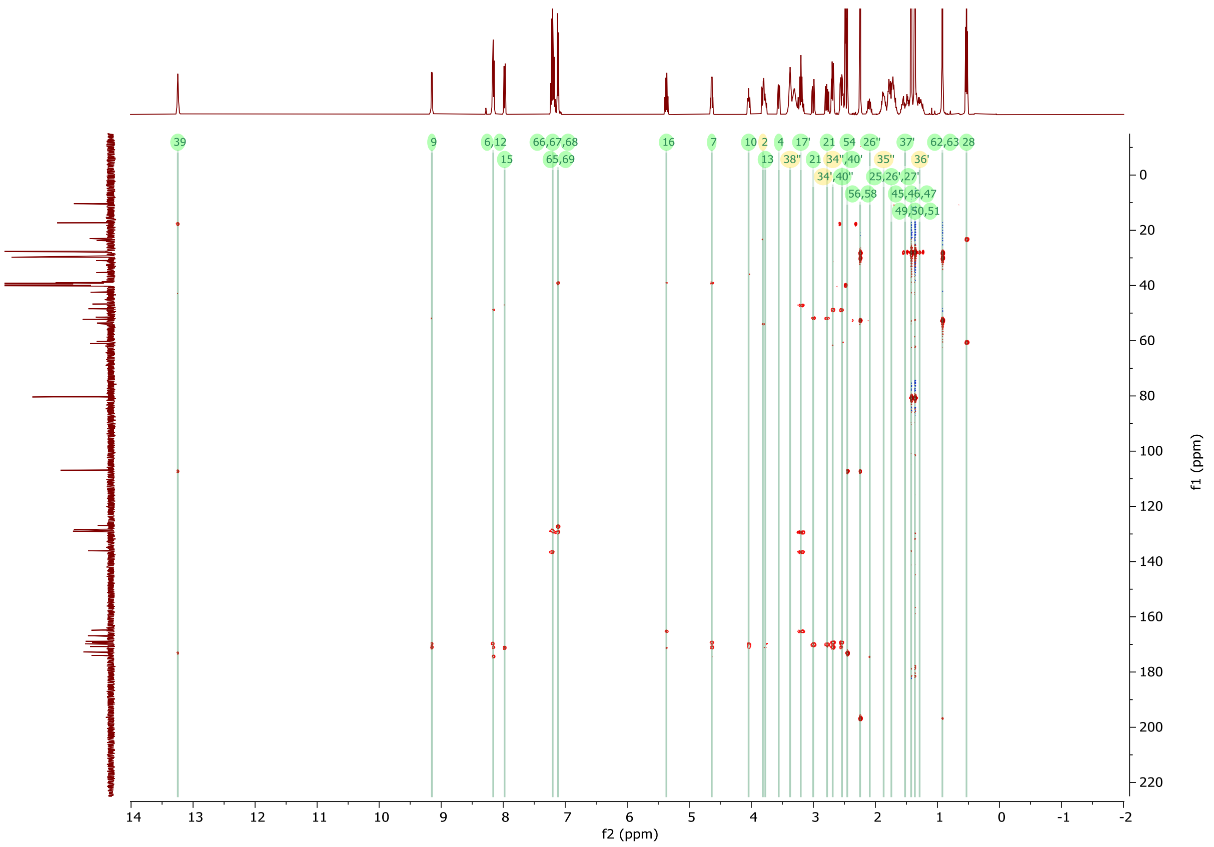

cyclo[PGLK(Boc)FA] odz/ethyl (8a)

^{1}H NMR (500 MHz, DMSO-d_6) at 25°C

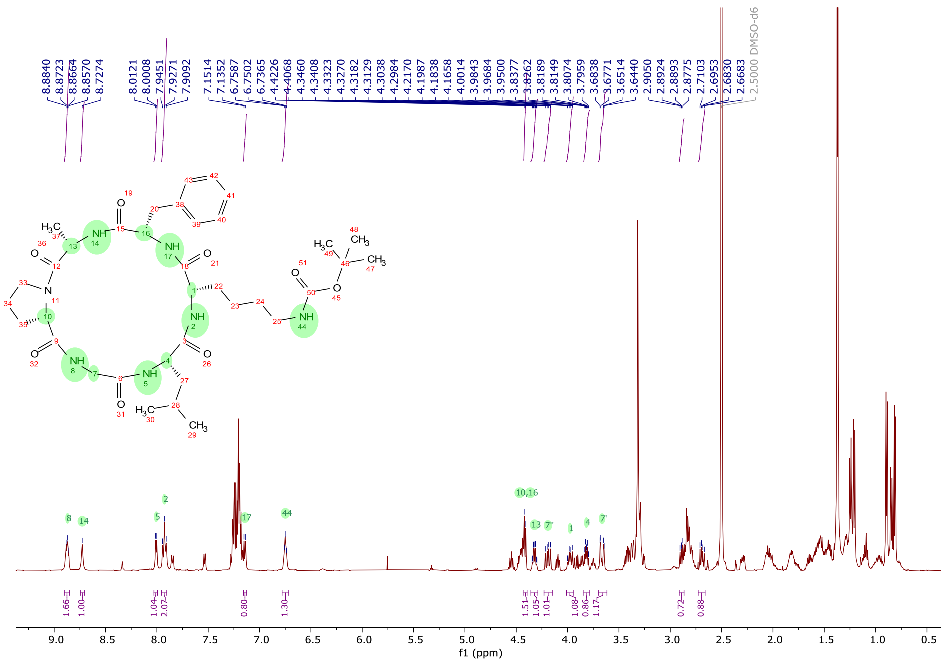

COSY at 25°C

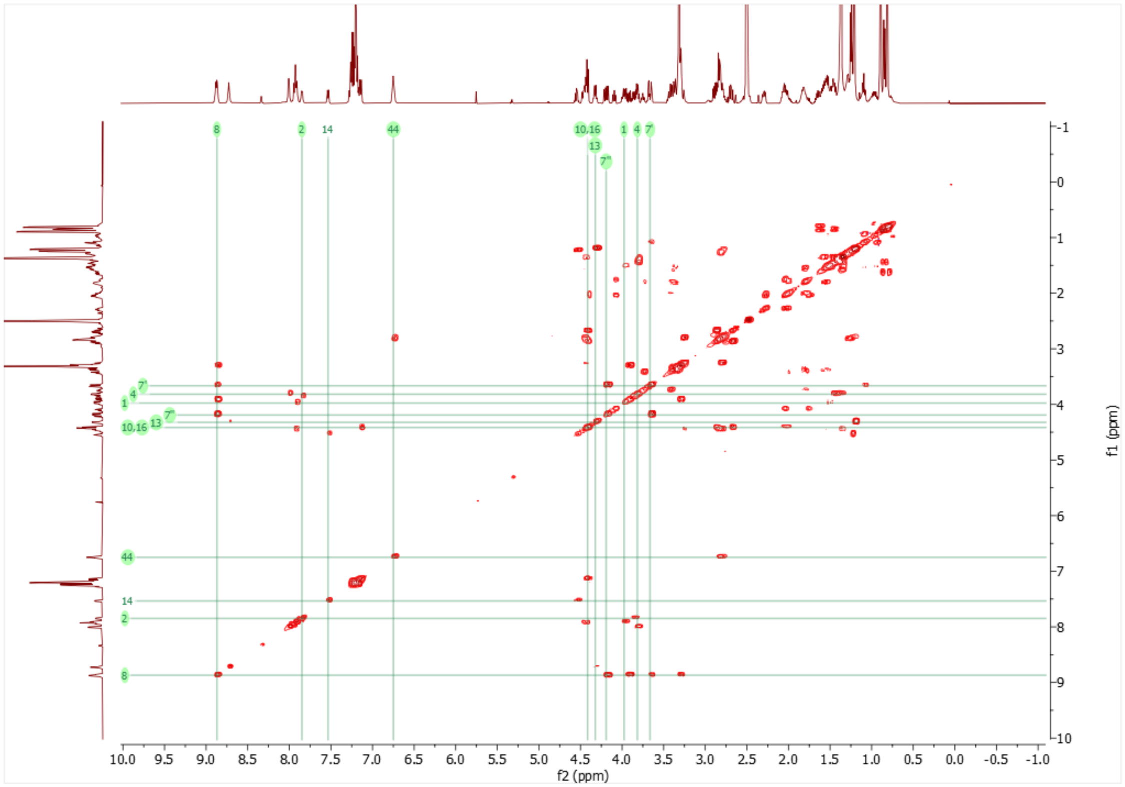

TOCSY at 25°C

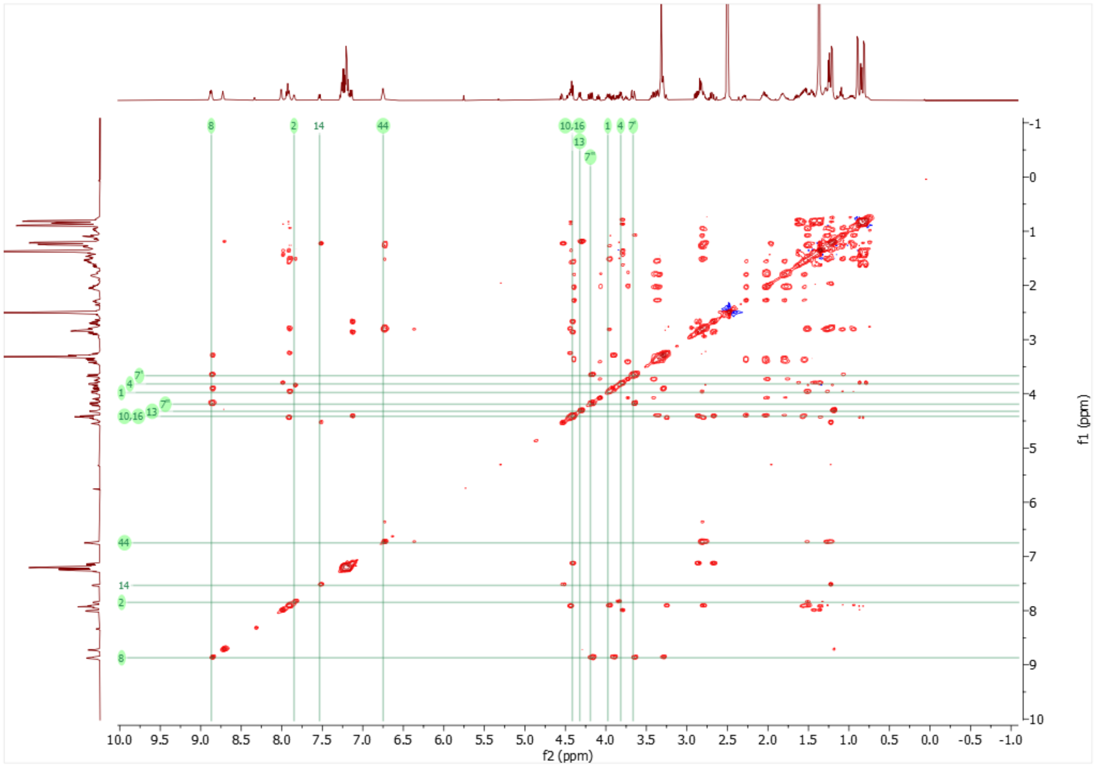

9 Variable NMR spectra

VT NMR of 1a

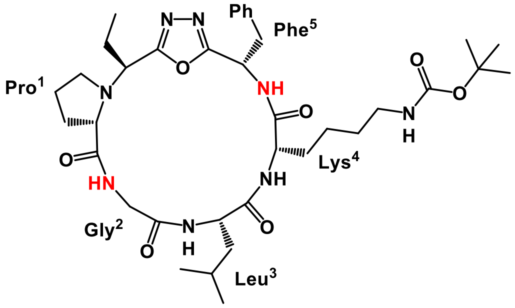

Temp (K)	Gly²-NH	Leu³-NH	Lys⁴-NH	Phe⁵-NH	Boc-NH
298	7.84	8.43	8.27	7.93	6.75
303	7.83	8.41	8.25	7.92	6.72
308	7.83	8.39	8.23	7.91	6.68
313	7.82	8.37	8.21	7.90	6.65
318	7.81	8.34	8.18	7.89	6.62
T_{coeff}	-1.78	-4.36	-4.41	-1.66	-6.39

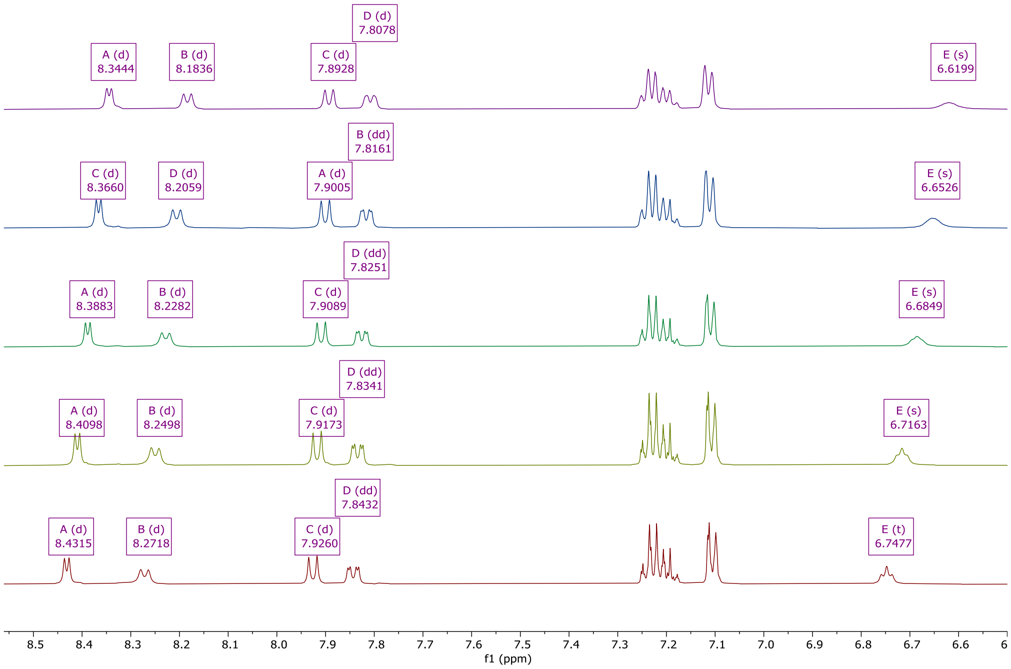

VT NMR of 2a

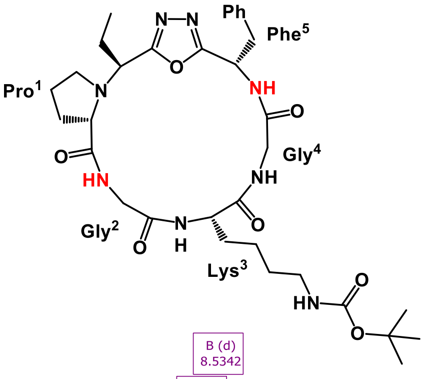

Temp (K)	Gly²-NH	Lys³-NH	Gly⁴-NH	Phe⁵-NH	Boc-NH
298	7.87	8.62	8.67	8.01	6.76
303	7.87	8.60	8.65	8.00	6.73
308	7.85	8.58	8.62	7.99	6.70
313	7.84	8.56	8.60	7.99	6.66
318	7.84	8.53	8.57	7.98	6.63
T_{coeff}	-1.92	-4.50	-4.91	-1.54	-6.19

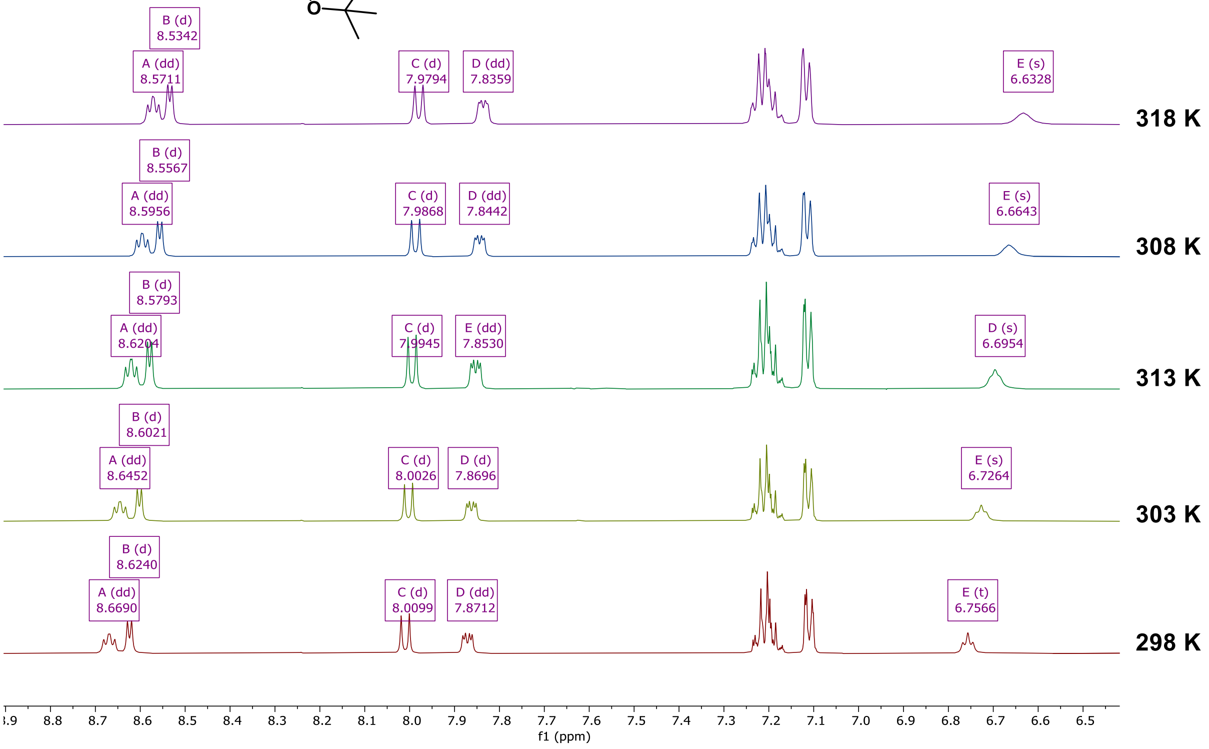

VT NMR of 3a

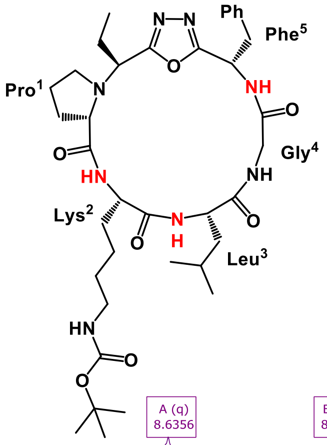

Temp (K)	Lys^{2}-NH	Leu^{3}-NH	Gly^{4}-NH	Phe^{5}-NH	Boc-NH
298	8.21	8.71	8.74	8.27	6.71
303	8.20	8.69	8.72	8.27	6.68
308	8.19	8.67	8.67	8.26	6.64
313	8.17	8.65	8.65	8.26	6.61
318	8.16	8.64	8.64	8.25	6.58
T_{coeff}	-2.56	-3.8	-5.45	-1.22	-6.22

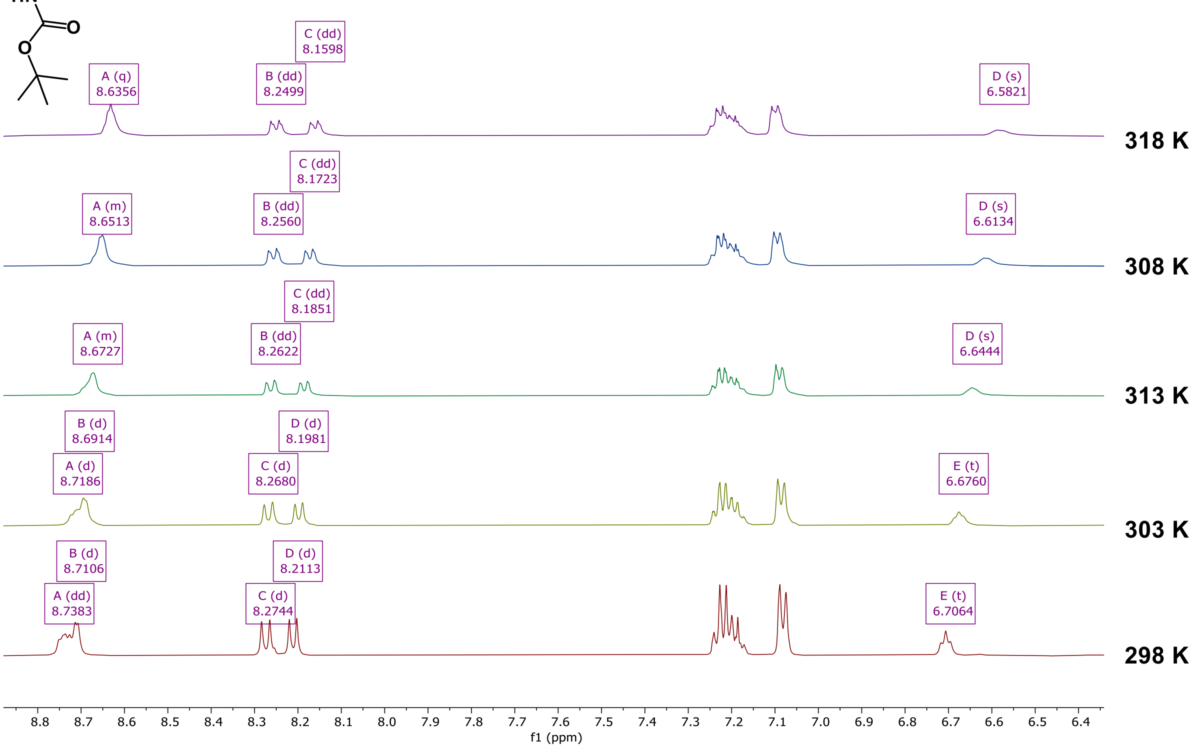

VT NMR of 4a

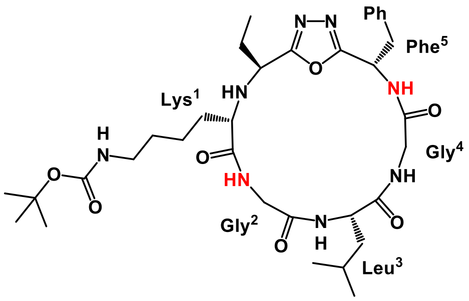

Temp (K)	Gly^2-NH	Leu^3-NH	Gly^4-NH	Phe^5-NH	Boc-NH
298	7.93	8.41	8.49	8.16	6.74
303	7.92	8.39	8.47	8.15	6.71
308	7.90	8.36	8.44	8.13	6.68
313	7.88	8.34	8.41	8.12	6.65
318	7.87	8.31	8.39	8.10	6.62
T_{coeff}	-3.36	-4.95	-5.15	-3.14	-6.31

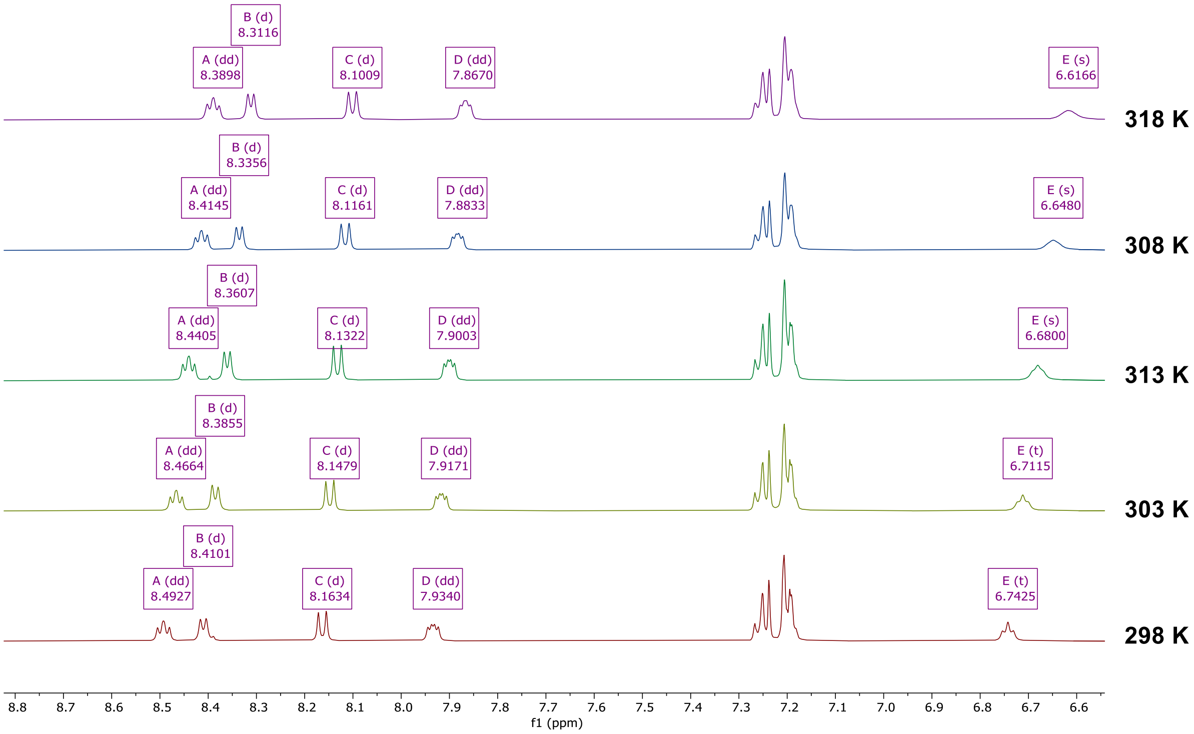

VT NMR of 5a

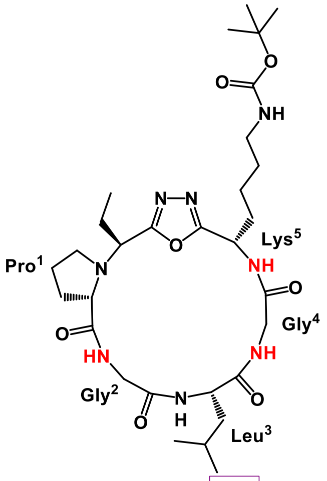

Temp (K)	Gly2-NH	Leu3-NH	Gly4-NH	Lys5-NH	Boc-NH
298	8.07	8.71	8.59	7.92	6.72
303	8.05	8.69	8.57	7.91	6.69
308	8.04	8.67	8.55	7.90	6.66
313	8.03	8.65	8.53	7.89	6.63
318	8.02	8.62	8.51	7.88	6.60
T_{coeff}	-2.15	-4.65	-3.92	-2.02	-6.12

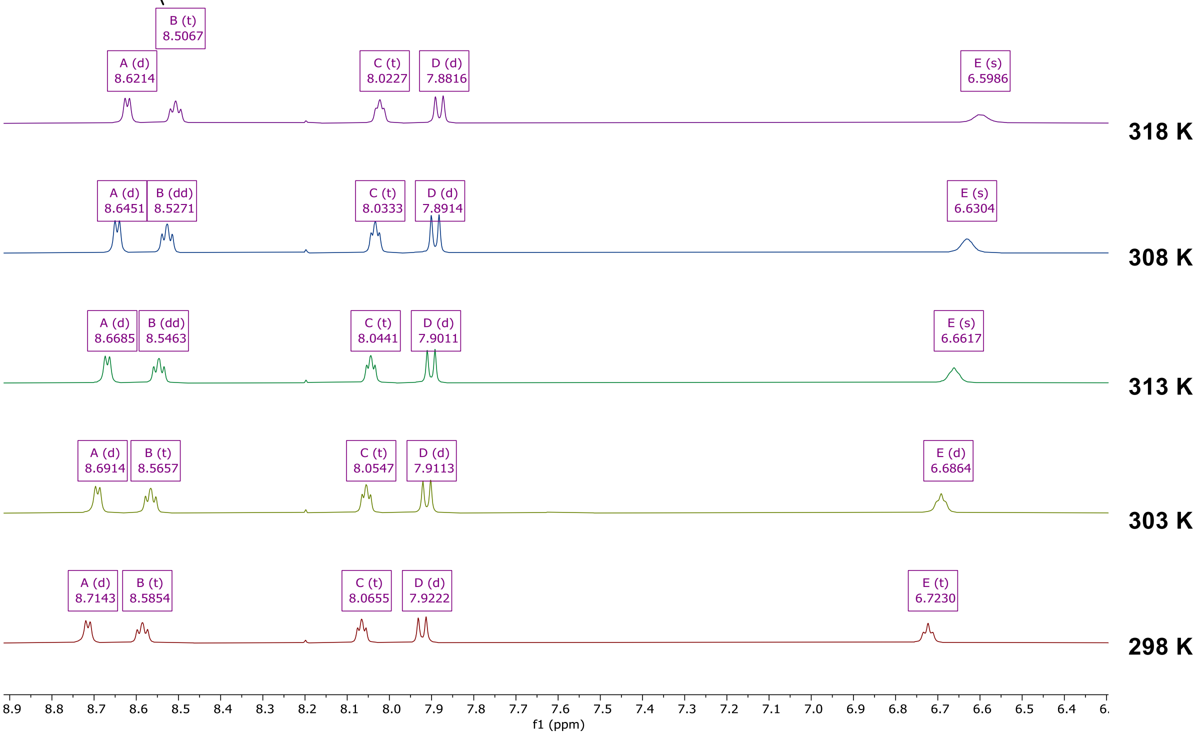

VT NMR of 6a

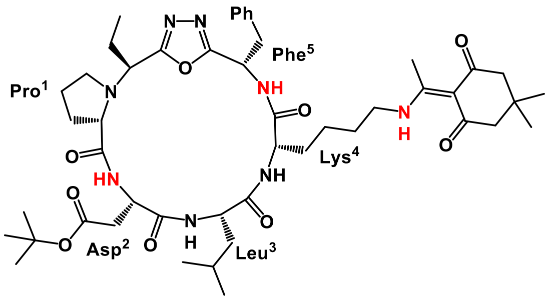

Temp (K)	Asp^2-NH	Leu^3-NH	Lys^4-NH	Phe^5-NH	Dde-NH
298	8.25	8.74	8.17	7.93	13.25
303	8.24	8.72	8.15	7.93	13.25
308	8.23	8.70	8.12	7.93	13.25
313	8.22	8.67	8.09	7.92	13.25
318	8.21	8.65	8.06	7.92	13.25
T_{coeff}	-2.09	-4.45	-5.44	-0.69	-0.14

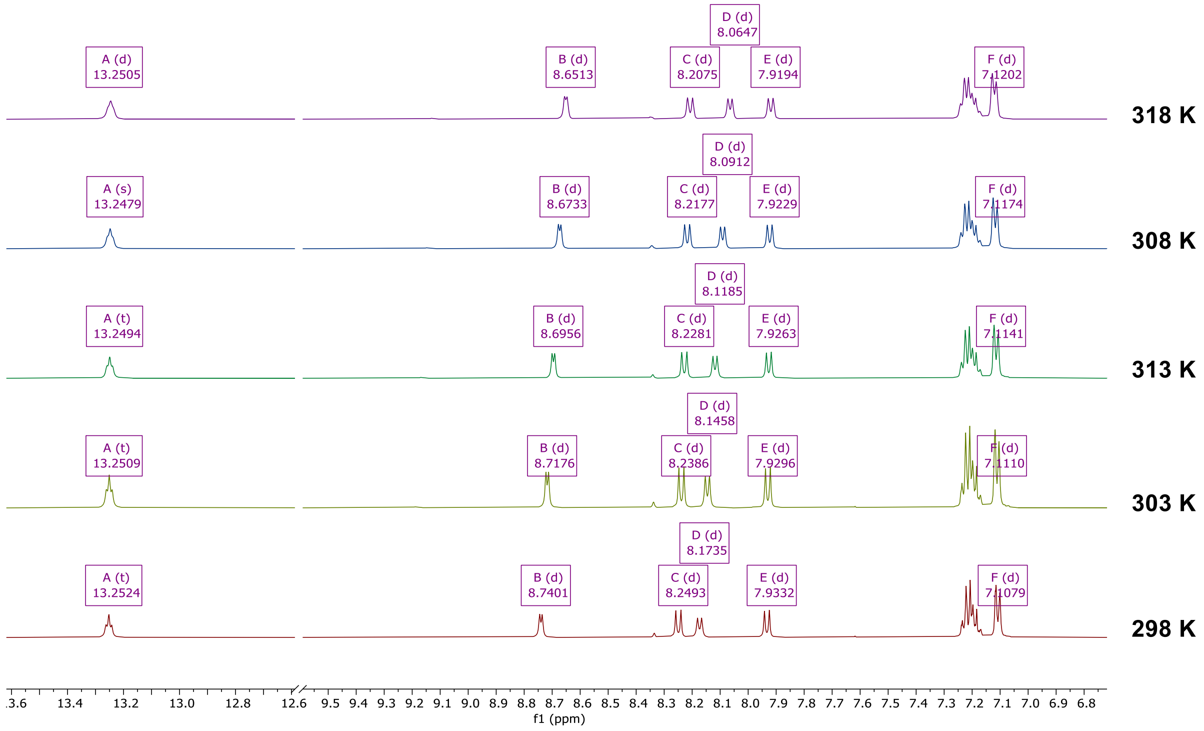

VT NMR of 7a

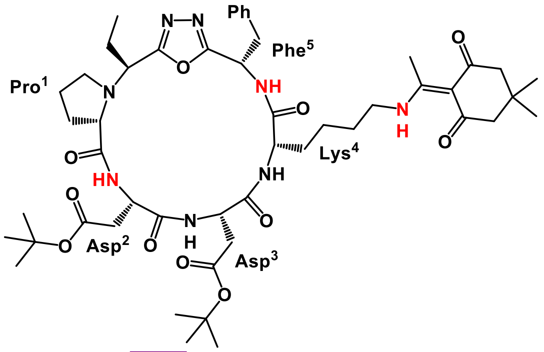

Temp (K)	Asp[2]-NH	Asp[3]-NH	Lys[4]-NH	Phe[5]-NH	Boc-NH
298	8.17	9.17	8.19	8.00	13.26
303	8.16	9.14	8.16	7.98	13.26
308	8.15	9.11	8.14	7.97	13.26
313	8.14	9.08	8.12	7.96	13.26
318	8.14	9.05	8.10	7.95	13.25
T_{coeff}	-1.85	-6.18	-4.59	-2.20	-0.65

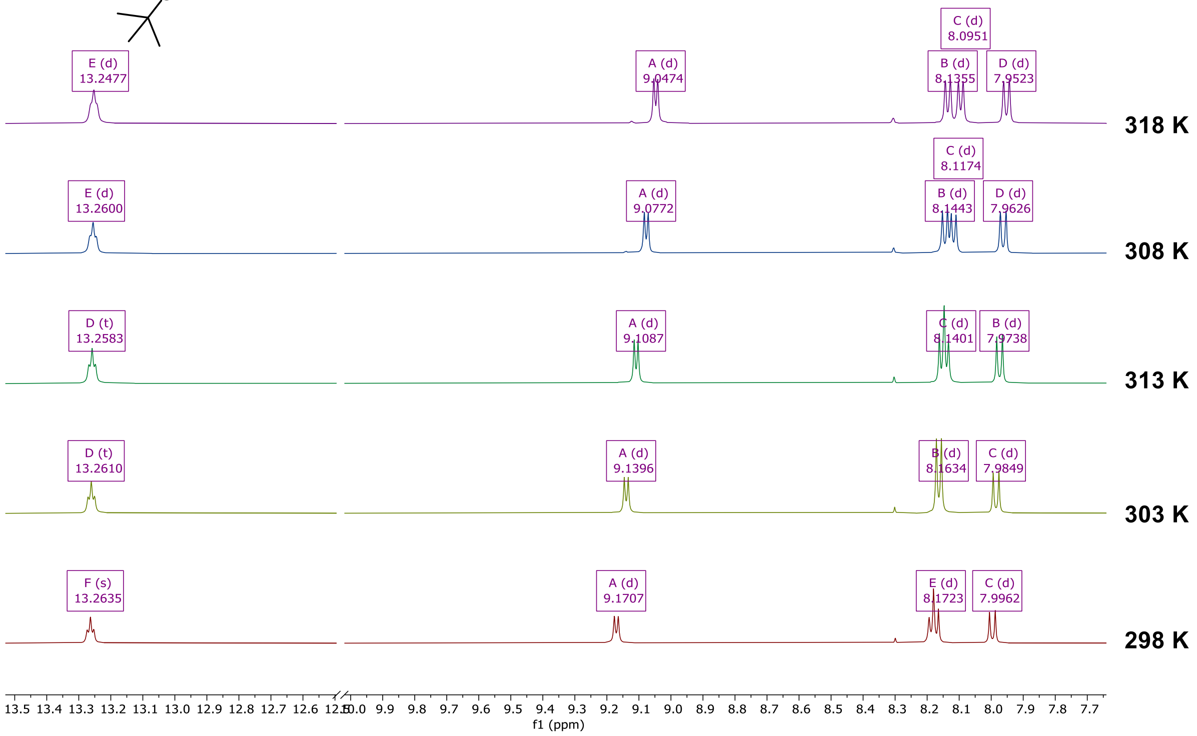

10 Reference

(1) Bio, M. M.; Javadi, G.; Song, Z. J. An Improved Synthesis of N-Isocyanoiminotriphenylphosphorane and Its Use in the Preparation of Diazoketones. *Synthesis (Stuttg)* **2005**, *2005* (01), 19–21. https://doi.org/10.1055/S-2004-834928.

(2) Bycroft, B. W.; Chan, W. C.; Chhabra, S. R.; Hone, N. D. A Novel Lysine-Protecting Procedure for Continuous Flow Solid Phase Synthesis of Branched Peptides. *J Chem Soc Chem Commun* **1993**, No. 9, 778–779. https://doi.org/10.1039/C39930000778.

(3) Peraro, L.; Deprey, K. L.; Moser, M. K.; Zou, Z.; Ball, H. L.; Levine, B.; Kritzer, J. A. Cell Penetration Profiling Using the Chloroalkane Penetration Assay. *J Am Chem Soc* **2018**, *140*

(36), 11360–11369. https://doi.org/10.1021/JACS.8B06144/ASSET/IMAGES/LARGE/JA-2018-06144Z_0005.JPEG.

(4) Spare, L. K.; Menti, M.; Harman, D. G.; Aldrich-Wright, J. R.; Gordon, C. P. A Continuous Flow Protocol to Generate, Regenerate, Load, and Recycle Chlorotrityl Functionalised Resins †. *Cite this: React. Chem. Eng* **2019**, *4*, 1309. https://doi.org/10.1039/c8re00318a.

(5) Peraro, L.; Deprey, K. L.; Moser, M. K.; Zou, Z.; Ball, H. L.; Levine, B.; Kritzer, J. A. Cell Penetration Profiling Using the Chloroalkane Penetration Assay. *J Am Chem Soc* **2018**, *140* (36), 11360–11369. https://doi.org/10.1021/jacs.8b06144.

(6) Deprey, K.; Kritzer, J. A. Quantitative Measurement of Cytosolic Penetration Using the Chloroalkane Penetration Assay. *Methods Enzymol* **2020**. https://doi.org/10.1017/CBO9781107415324.004.

(7) Ballister, E. R.; Aonbangkhen, C.; Mayo, A. M.; Lampson, M. A.; Chenoweth, D. M. Localized Light-Induced Protein Dimerization in Living Cells Using a Photocaged Dimerizer. *Nat Commun* **2014**, *5*, 5475. https://doi.org/10.1038/ncomms6475.

(8) Cerulli, R. A.; Shehaj, L.; Brown, H.; Pace, J.; Mei, Y.; Kritzer, J. A. Stapled Peptide Inhibitors of Autophagy Adapter LC3B. *ChemBioChem* **2020**, *21* (19), 2777–2785. https://doi.org/10.1002/cbic.202000212.

(9) Naylor, M. R.; Ly, A. M.; Handford, M. J.; Ramos, D. P.; Pye, C. R.; Furukawa, A.; Klein, V. G.; Noland, R. P.; Edmondson, Q.; Turmon, A. C.; Hewitt, W. M.; Schwochert, J.; Townsend, C. E.; Kelly, C. N.; Blanco, M. J.; Lokey, R. S. Lipophilic Permeability Efficiency Reconciles the Opposing Roles of Lipophilicity in Membrane Permeability and Aqueous Solubility. *J Med Chem* **2018**, *61* (24), 11169–11182. https://doi.org/10.1021/ACS.JMEDCHEM.8B01259/SUPPL_FILE/JM8B01259_SI_007.CSV.

(10) Frost, J. R.; Scully, C. C. G.; Yudin, A. K. Oxadiazole Grafts in Peptide Macrocycles. *Nature Chemistry 2016 8:12* **2016**, *8* (12), 1105–1111. https://doi.org/10.1038/nchem.2636.
